# Pro-maturational Effects of Human iPSC-Derived Cortical Astrocytes upon iPSC-Derived Cortical Neurons

**DOI:** 10.1016/j.stemcr.2020.05.003

**Published:** 2020-06-04

**Authors:** Anne Hedegaard, Jimena Monzón-Sandoval, Sarah E. Newey, Emma S. Whiteley, Caleb Webber, Colin J. Akerman

**Affiliations:** 1 Department of Pharmacology, University of Oxford, Mansfield Road, Oxford OX1 3QT, UK; 2 UK Dementia Research Institute, Cardiff University, Maindy Road, Cardiff CF24 4HQ, UK; 3 Department of Physiology, Anatomy and Genetics, University of Oxford, South Parks Road, Oxford OX1 3QX, UK

**Keywords:** induced pluripotent stem cells, astrocytes, co-culture, synaptic maturation, astrocyte-neuron interactions, optogenetics, gliotransmission

## Abstract

Astrocytes influence neuronal maturation and function by providing trophic support, regulating the extracellular environment, and modulating signaling at synapses. The emergence of induced pluripotent stem cell (iPSC) technology offers a human system with which to validate and re-evaluate insights from animal studies. Here, we set out to examine interactions between human astrocytes and neurons derived from a common cortical progenitor pool, thereby recapitulating aspects of *in vivo* cortical development. We show that the cortical iPSC-derived astrocytes exhibit many of the molecular and functional hallmarks of astrocytes. Furthermore, optogenetic and electrophysiological co-culture experiments reveal that the iPSC-astrocytes can actively modulate ongoing synaptic transmission and exert pro-maturational effects upon developing networks of iPSC-derived cortical neurons. Finally, transcriptomic analyses implicate synapse-associated extracellular signaling in the astrocytes' pro-maturational effects upon the iPSC-derived neurons. This work helps lay the foundation for future investigations into astrocyte-to-neuron interactions in human health and disease.

## Highlights

• Human astrocytes and neurons are generated from a common cortical progenitor pool
• Astrocyte-neuron signaling is demonstrated with neurotransmitters and optogenetics
• Astrocyte co-culture promotes cortical neuron and synaptic network maturation
• Transcriptomics reveal extracellular astrocytic proteins that interact at synapses

## Graphical Abstract

**Figure:**
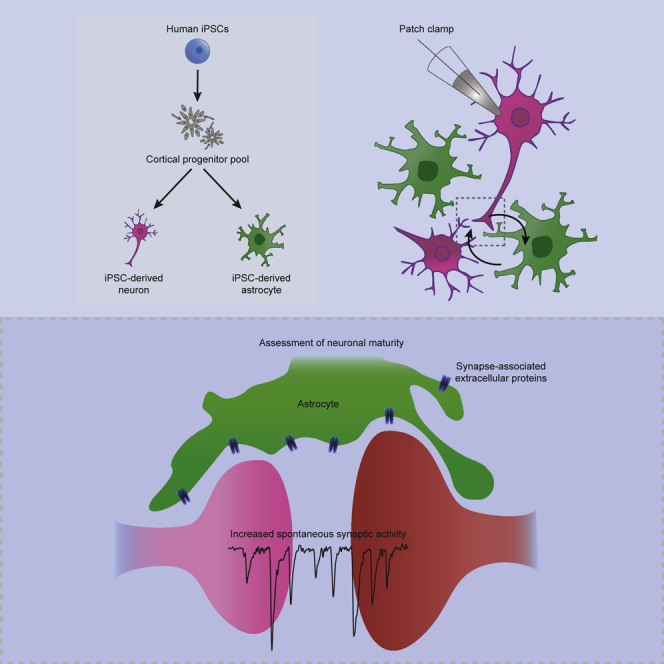

## Teaser

In this article, Akerman and colleagues provide a molecular and functional characterization of human iPSC-derived cortical astrocytes. The astrocytes can modulate ongoing excitatory synaptic transmission between iPSC-derived cortical neurons. When co-cultured, the astrocytes enhance the functional maturation of cortical synaptic networks. Finally, transcriptomic analyses indicate that the astrocytes engage in synapse-associated extracellular signaling to mediate their pro-maturational effects.

## Introduction

Most of our knowledge regarding astrocyte-neuron interactions has been gained from animal models, which have been particularly important in advancing understanding at a molecular and cellular level. From such studies, we know that during development and in the mature brain, astrocytes provide important trophic and homeostatic support to neurons (Verkhratsky and Nedergaard, 2018). Astrocyte-to-neuron signaling is thought to promote many aspects of neuronal development, including the emergence of electrical excitability and the formation and maturation of synapses (Allen and Eroglu, 2017). Through their close association with synapses, astrocytes also support ongoing neurotransmission by regulating concentration changes in extracellular ions and neurotransmitters (Perea and Araque, 2010, Verkhratsky and Nedergaard, 2014) and function as a glio-neuronal processing unit that can monitor and modulate ongoing synaptic transmission on rapid timescales (Fellin and Carmignoto, 2004, Panatier et al., 2011).

The development of induced pluripotent stem cell (iPSC) technology offers the potential to test established knowledge gained from rodent models and, importantly, provides opportunities to test hypotheses in a human disease context. By adapting culture conditions, iPSCs can give rise to specific cell types in a process that replicates key aspects of *in vivo* development. To generate human astrocytes, iPSC differentiation protocols have generally targeted the gliogenic JAK-STAT pathway via manipulations of culture media. This strategy enables re-capitulation of stages of *in vivo* development leading to astrocyte production over different timescales (Krencik et al., 2011, Serio et al., 2013, Shaltouki et al., 2013, Sloan et al., 2017), and with the potential for regional patterning during the preceding neurogenic stage (Liu and Zhang, 2011, Roybon et al., 2013). Another strategy has been to directly convert fibroblasts into astrocytes via overexpression of transcription factors associated with the JAK-STAT pathway (Canals et al., 2018, Tchieu et al., 2019). Such approaches have led to recent co-culture studies in which iPSC-derived astrocytes are reported to exhibit pro-maturational effects upon co-cultured neurons, such as enhancing the intrinsic excitability of the neurons (Kayama et al., 2018, Klapper et al., 2019, VanderWall et al., 2019). Meanwhile, co-culturing with iPSC-derived astrocytes has had mixed results in terms of influencing synaptic transmission and synaptic maturation. Some reports have shown that iPSC-derived astrocytes can enhance the synaptic signaling between iPSC-derived retinal ganglion cells (VanderWall et al., 2019) and so-called induced neurons (Canals et al., 2018, Tchieu et al., 2019), whereas others have not observed such effects, perhaps due to the level of astrocyte maturity (Lischka et al., 2018). Meanwhile, to our knowledge, rapid astrocyte-mediated modulation of ongoing synaptic transmission has not previously been demonstrated in an iPSC-derived human co-culture.

During *in vivo* cortical development, neurons and astrocytes are thought to derive from a common progenitor pool (Rowitch and Kriegstein, 2010). Radial glial cells, the principal progenitor cell type in embryonic cortex, initially undergo asymmetrical divisions to produce neurons or neurogenic intermediate progenitor cells. As the period of neurogenesis finishes, there is a switch to gliogenesis, and radial glial cells can give rise to astrocytes (Rowitch and Kriegstein, 2010). Here, we set out to study the interactions between human astrocytes and neurons independently generated from a common cortical progenitor pool. The molecular identity and functional properties of the astrocytes are determined by immunocytochemistry, transcriptomics, and targeted recordings. Co-culture studies then demonstrate that the cortically derived astrocytes exhibit key interactions with iPSC-derived cortical neurons, including the ability to rapidly modulate ongoing synaptic signaling and to exert pro-maturational effects on synaptic networks. Consistent with this, transcriptomic analyses identify astrocytic extracellular signaling at neuronal pre- and post-synaptic sites.

## Results

### Deriving Human Astrocytes and Neurons from a Common Cortical Progenitor Pool

**Figure 1:**
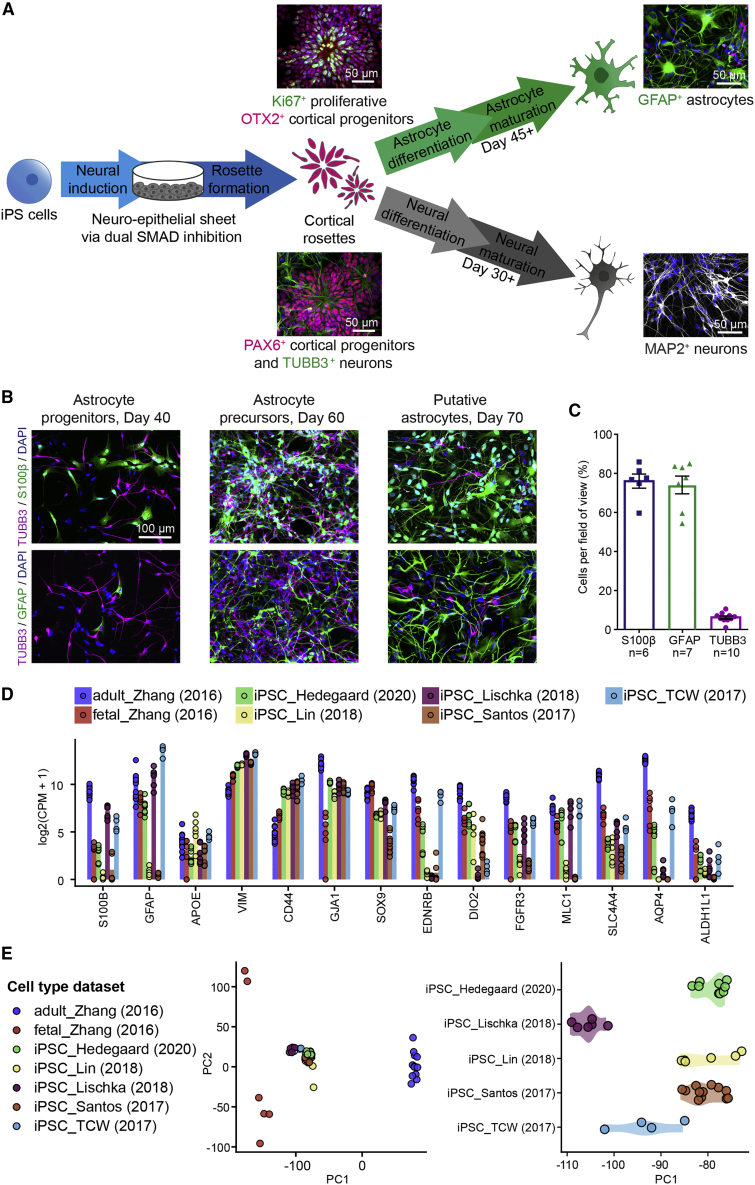
Human Cortically Derived iPSC-Astrocytes Express Canonical Markers and Are Comparable with Other iPSC-Astrocytes (A) Schematic of the differentiation from iPSCs toward a common pool of PAX6^+^ cortical progenitor cells, which can then be driven toward generating either GFAP^+^ astrocytes or MAP2^+^ cortical neurons. See also Figure S1. (B) Examples of increasing expression of astrocytic markers, S100β (top) and GFAP (bottom), tracked throughout the astrocyte differentiation and maturation process. (C) Proportion of S100β^+^, GFAP^+^, and TUBB3^+^ cells quantified from four cultures across three cell lines, at the mature stage of the astrocyte protocol (sample sizes represent fields of view per marker, 70 ±5.6 days at imaging). (D) Gene expression levels for astrocyte-specific markers in iPSC-astrocytes generated from the present study (iPSC_Hedegaard; n = 9 cultures, comprising three cultures from each of three cell lines, 96 ± 3.3 days). For comparison, transcript abundances of fetal and adult human cortical astrocytes (Zhang et al., 2016) and previously published profiles of iPSC-derived astrocytes (Lin et al., 2018, Lischka et al., 2018, Santos et al., 2017, Tcw et al., 2017) are included. Gene expression levels are logarithm scaled counts per million (log(CPM +1 )). See also Figure S2. (E) Principal component analysis separated fetal and adult astrocytes from Zhang et al. (2016) along the first component. Gene expression profiles from published iPSC-astrocytes datasets were projected onto the space created by the first two principal components (left). PC1 discriminates iPSC-astrocytes by dataset (right).

Since cortical neurons and astrocytes can originate from the same progenitors *in vivo* (Rowitch and Kriegstein, 2010), we set out to generate iPSC-derived neurons and astrocytes from a common cortical progenitor pool. The protocol involves initiation of the cortical differentiation pathway via dual SMAD inhibition (Chambers et al., 2009), as part of a well-established cortical neuron induction protocol (Shi et al., 2012). This approach produced self-organizing rosette structures composed of PAX6^+^ radial glia progenitors, the cortical identity of which was verified by widespread OTX2 expression. These radial glia progenitors were subsequently directed down one of two differentiation pathways to generate either MAP2^+^ neurons or cells of an astrocytic fate (Figure 1A, further details in Figure S1). We refer to these as cortical iPSC-neurons and cortical iPSC-astrocytes, respectively.

We first tracked the commitment to astrocytic fate by immunofluorescence staining (described in Supplemental Experimental Procedures) for the classic astrocyte markers, S100β and GFAP (Ludwin et al., 1976). At the putative astrocyte progenitor stage (<40 days), S100β and GFAP were sparsely expressed (Figure 1B). By ∼60 days, staining was consistent with an astrocyte precursor stage at which S100β is more abundant than GFAP (Barnabé-Heider et al., 2005). After a further 10–20 days, all cell lines were enriched for putative astrocytes. Quantification showed that as a proportion of all DAPI^+^ cells, 76% were S100β^+^ and 74% were GFAP^+^, while only 6% were expressing the neuronal marker TUBB3^+^ (Figure 1C).

We next used RNA sequencing (RNA-seq) analysis to gain greater insight into the expression profile of our iPSC-astrocytes. We compared our data with other published transcriptomic datasets on human iPSC-derived astrocytes, as well as RNA-seq profiles of primary human fetal and adult cortical astrocytes (Zhang et al., 2016; Figure 1D). Our iPSC-astrocytes expressed a set of well-known astrocyte-specific genes (Zhang et al., 2016) at either similar levels (e.g., *APOE, VIM*, *CD44*, and *GJA1*) or higher levels (e.g., *AQP4*, *EDNRB*, and *DIO2*), compared with other iPSC-astrocyte differentiation protocols (Figure 1D). All iPSC-astrocyte populations were also similar in terms of the expression of reactive markers, consistent with the idea that *in vitro* cell culture tends to lead to a more reactive astrocytic state (Cahoy et al., 2008, Liddelow and Barres, 2017) (Figure S2). To assess the relative maturity of our iPSC-astrocytes, we projected the RNA-seq profiles of iPSC-astrocytes onto the principal component (PC) space created from the gene expression profiles of the primary fetal and adult astrocytes (Figure 1E). iPSC-derived astrocytes generally clustered together and were closer to primary fetal astrocytes than to adult astrocytes, although modest differences along PC1 were apparent between the different iPSC-astrocyte populations (Figure 1E). These molecular marker and gene expression data suggest that astrocytes derived from a cortical progenitor population are comparable with other iPSC-derived astrocytes.

### Functional Characterization of Cortically Derived Human iPSC-Astrocytes

**Figure 2:**
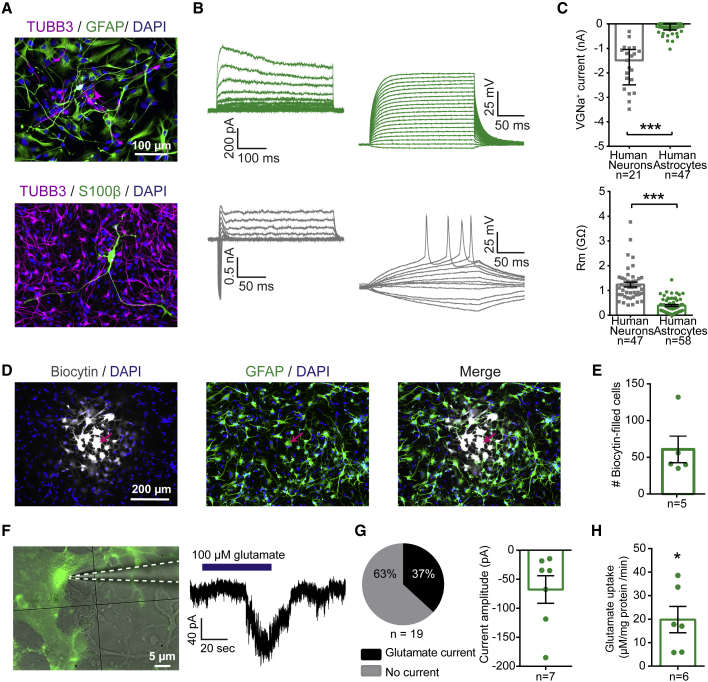
Functional Characteristics of Cortically Derived iPSC-Astrocytes (A) Example GFAP^+^ astrocyte culture (55 days; top) and TUBB3^+^ neuronal culture (100 days; bottom). Cultures were highly enriched for their respective cell type, but low numbers of TUBB3^+^ cells were observed in astrocyte cultures, and low numbers of S100β^+^ astrocytes were observed in long-term neuronal cultures. (B) Example leak-subtracted current responses (left) and voltage responses (right) from an iPSC-astrocyte (99 days; top) and an iPSC-neuron (91 days; bottom). (C) Comparison of peak voltage-gated sodium (VGNa^+^) currents (top) and membrane resistance (Rm; bottom) between astrocytes and neurons. Sample sizes represent individual cells recorded from 14 cultures across four cell lines (astrocytes 97 ± 3.5 days) and 13 cultures across three cell lines (neurons 92 ± 3.3 days). (D) Example gap-junction connected iPSC-astrocytes revealed by filling a single astrocyte with biocytin (arrow; 103 days). (E) Number of gap-junction connected cells quantified from immunolabeling with streptavidin (n = 5 patched astrocytes from a 103-day-old culture). (F) Example whole-cell patch-clamp recording from an iPSC-astrocyte (123 days; left) showing an inward current in response to bath application of glutamate (right), consistent with uptake (Dallas et al., 2007). (G) Proportion of iPSC-astrocytes that showed a significant glutamate-uptake current (left; n = 19 astrocytes recorded from eight cultures across four cell lines, 95 ± 5.3 days; see Supplemental Experimental Procedures). Current amplitudes are shown for the astrocytes that exhibited glutamate uptake (right). (H) Glutamate uptake capacity of iPSC-astrocytes assessed by an enzymatic absorbance assay (n = 6 cultures comprising two cultures from each of three cell lines, 73 ± 6.7 days; see Supplemental Experimental Procedures). ^∗^p < 0.05, ^∗∗∗^p < 0.001.

In contrast to neurons, astrocytes are regarded as non-excitable under physiological conditions. In keeping with this, none of our iPSC-astrocytes could generate action potentials (Figures 2A and 2B), and while all neurons displayed large voltage-gated Na^+^ (VGNa^+^) currents, the iPSC-astrocytes lacked Na^+^ currents of comparable size (astrocytes, −0.18 ± 0.03 nA; neurons, −1.7 ± 0.2 nA; p < 0.0001, unpaired Mann-Whitney test; Figures 2B and 2C). The iPSC-astrocytes also exhibited lower membrane resistances, consistent with more permeable membranes (astrocytes, 0.39 ± 0.04 GΩ; neurons, 1.2 ± 0.1 GΩ; p < 0.0001, unpaired Mann-Whitney test; Figure 2C). *In vivo*, syncytia of gap-junction-coupled astrocytes are considered vital for regulating the levels of substances in the extracellular space, with a primary function to clear and disperse excess glutamate and K^+^ (Theis et al., 2005, Verkhratsky and Nedergaard, 2018). Firstly, the presence of gap junctions was revealed by patching and filling individual iPSC-astrocytes with biocytin (Figure 2D), which spread to an average of 61 ± 18 cells (Figure 2E) and overlapped with GFAP staining. Secondly, the activity of astrocytic glutamate transporters was detected both as a net inward current in individual astrocytes following glutamate application (37% of recorded cells; Figures 2F and 2G) and at a population level using an enzymatic glutamate uptake assay (astrocytic uptake, 19.8 ± 5.6 μM/mg protein/min; p = 0.031, one-sample Wilcoxon signed-rank test; Figure 2H; see details on both glutamate uptake assays in Supplemental Experimental Procedures). Thirdly, a subset of the iPSC-astrocytes displayed inward rectifying K^+^ currents (Figure S3), consistent with a contribution of K_ir_4.1 channels, which are implicated in K^+^ and glutamate buffering (Djukic et al., 2007). Together these results demonstrate that the cortically derived iPSC-astrocytes exhibit non-excitable biophysical properties, gap-junction coupling, plus the capacity for K^+^ and glutamate buffering, which are key functional properties associated with astrocytes.

### Cortically Derived iPSC-Astrocytes Respond to Neurotransmitters and Engage in Rapid Astrocyte-to-Neuron Signaling

**Figure 3:**
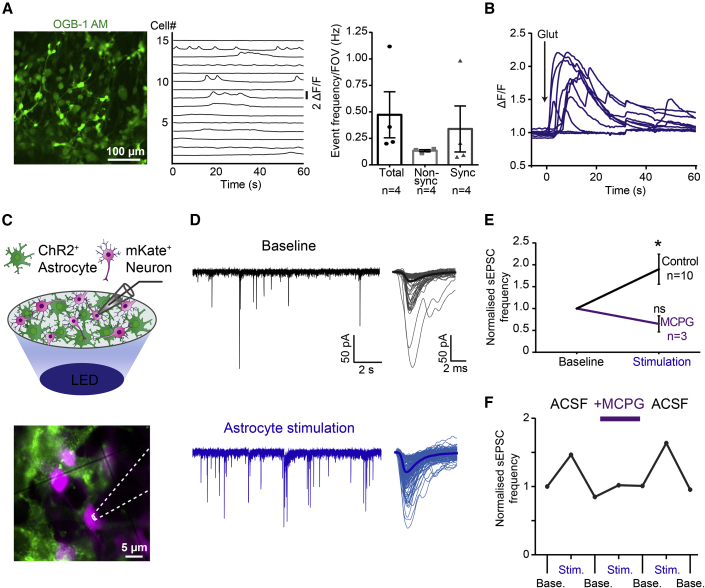
Cortically Derived iPSC-Astrocytes Engage in Ca^2+^ Signaling and Astrocyte-to-Neuron Gliotransmission (A) Human iPSC-astrocyte culture loaded with OGB-1 AM dye (left; 96 days) and ΔF/F traces from 15 astrocytes in the same culture (middle). Population data on spontaneous Ca^2+^ event frequency (right) for either all Ca^2+^ events in a recording (Total), Ca^2+^ events occurring in individual astrocytes (Non-sync), or Ca^2+^ events occurring simultaneously across multiple astrocytes (Sync) (n = 4 fields of view [FOV] from four cultures across three cell lines, 101 ± 3.9 days). (B) Time-locked Ca^2+^ events were elicited by focal delivery of 100 μM glutamate via a patch pipette (n = 10 astrocytes from a 96-day-old culture). (C) Optogenetic experimental design for assessing astrocyte-to-neuron gliotransmission (top). mKate^+^ iPSC-neurons (magenta) were targeted for recordings (bottom), and co-cultured ChR2^+^ iPSC-astrocytes (green) were optically stimulated via a blue LED. (D) Example spontaneous excitatory synaptic currents (sEPSCs) recorded from an iPSC-neuron (neurons 60 days at recording, astrocytes 89 days, co-cultured for 20 days) under baseline conditions (top) and during astrocyte stimulation (450 nm LED; 250 ms pulse duration at 2–4 Hz; bottom). An individual trace (left) and overlays of detected sEPSCs (right) are shown, with the average sEPSC in bold. (E) Optical stimulation of astrocytes generates a significant increase in sEPSC frequency under control conditions, which is blocked by the mGluR antagonist, MCPG (0.8 mM). All values normalized to baseline frequency. Sample sizes represent individual neurons recorded from two cultures, one cell line (neurons 58 ± 2 days at recording, astrocytes 103 ± 0.5 days, co-cultured for 21 ± 2.5 days). (F) Individual experiment in which astrocyte stimulation increased neuronal sEPSCs before application of MCPG, and after washout of MCPG, but not in the presence of MCPG. ^∗^p < 0.05.

Astrocytes are fine-tuned to sense local neuronal activity and respond by exhibiting both spontaneous and neurotransmitter-evoked intracellular Ca^2+^ increases (Aguado et al., 2002, Porter and McCarthy, 1996). To investigate Ca^2+^ signaling in the cortically derived iPSC-astrocytes, cultures were loaded with a membrane-permeable Ca^2+^ dye (OGB-1; Figure 3A; see details on Ca^2+^ imaging in Supplemental Experimental Procedures). In the absence of external stimuli, spontaneous Ca^2+^ events occurred in all astrocyte cultures, with an average of 41.5% of cells exhibiting events. These Ca^2+^ events could be classified as occurring either in an individual astrocyte (non-synchronous events, with a frequency of 0.13 ± 0.01 Hz) or simultaneously across multiple astrocytes (synchronous events, 0.34 ± 0.2 Hz; Figures 3A and S4). Furthermore, astrocytic Ca^2+^ events could be evoked by local application of glutamate (Figure 3B) or ATP (Figure S4) delivered via a patch pipette. Both neurotransmitters elicited robust and rapid Ca^2+^ elevations in the astrocytes.

Such neurotransmitter-evoked Ca^2+^ responses are often viewed as part of a reciprocal communication system between astrocytes and neurons, commonly referred to as gliotransmission (Di Castro et al., 2011, Navarrete et al., 2013, Panatier et al., 2011). We therefore investigated the potential of our cortically derived iPSC-astrocytes to signal in the opposite direction, from astrocyte to neuron. We performed co-culture experiments using iPSC-derived cortical neurons and adopted an optogenetic strategy used in rodent cortex, where astrocytic Ca^2+^ events have been shown to initiate glutamatergic gliotransmission that enhances synaptic transmission between nearby neurons (Perea et al., 2014, Sasaki et al., 2012). To achieve this, we recorded spontaneous excitatory post-synaptic currents (sEPSCs) from the iPSC-derived neurons, while selectively stimulating nearby iPSC-astrocytes expressing the depolarizing and Ca^2+^-permeable opsin, Channelrhodopsin-2 (ChR2; Nagel et al., 2003) (Figures 3C and 3D). Upon stimulation of the ChR2^+^ astrocytes with blue light pulses, a significant increase in the frequency of sEPSCs was observed in the neurons (1.9 ± 0.3-fold increase compared with baseline; p = 0.02, Wilcoxon signed-rank test; Figures 3D and 3E). Furthermore, this astrocyte-mediated enhancement in sEPSC frequency was dependent on metabotropic glutamate receptor (mGluR) activation, as has also been reported for rodent cortical astrocytes (Perea et al., 2014). In a subset of neurons confirmed to exhibit astrocyte-enhanced sEPSCs, the general mGluR blocker, α-methyl-4-carboxyphenylglycine (MCPG), abolished the effects of astrocyte stimulation (p = 0.5, Wilcoxon signed-rank test; Figures 3E and 3F). Taken together, these experiments demonstrate that the cortically derived iPSC-astrocytes are capable of engaging in bidirectional signaling with neurons, as they can both respond to neurotransmitters and influence ongoing synaptic transmission.

### Co-culture with Cortically Derived iPSC-Astrocytes Enhances Neuronal and Synaptic Network Maturation

**Figure 4:**
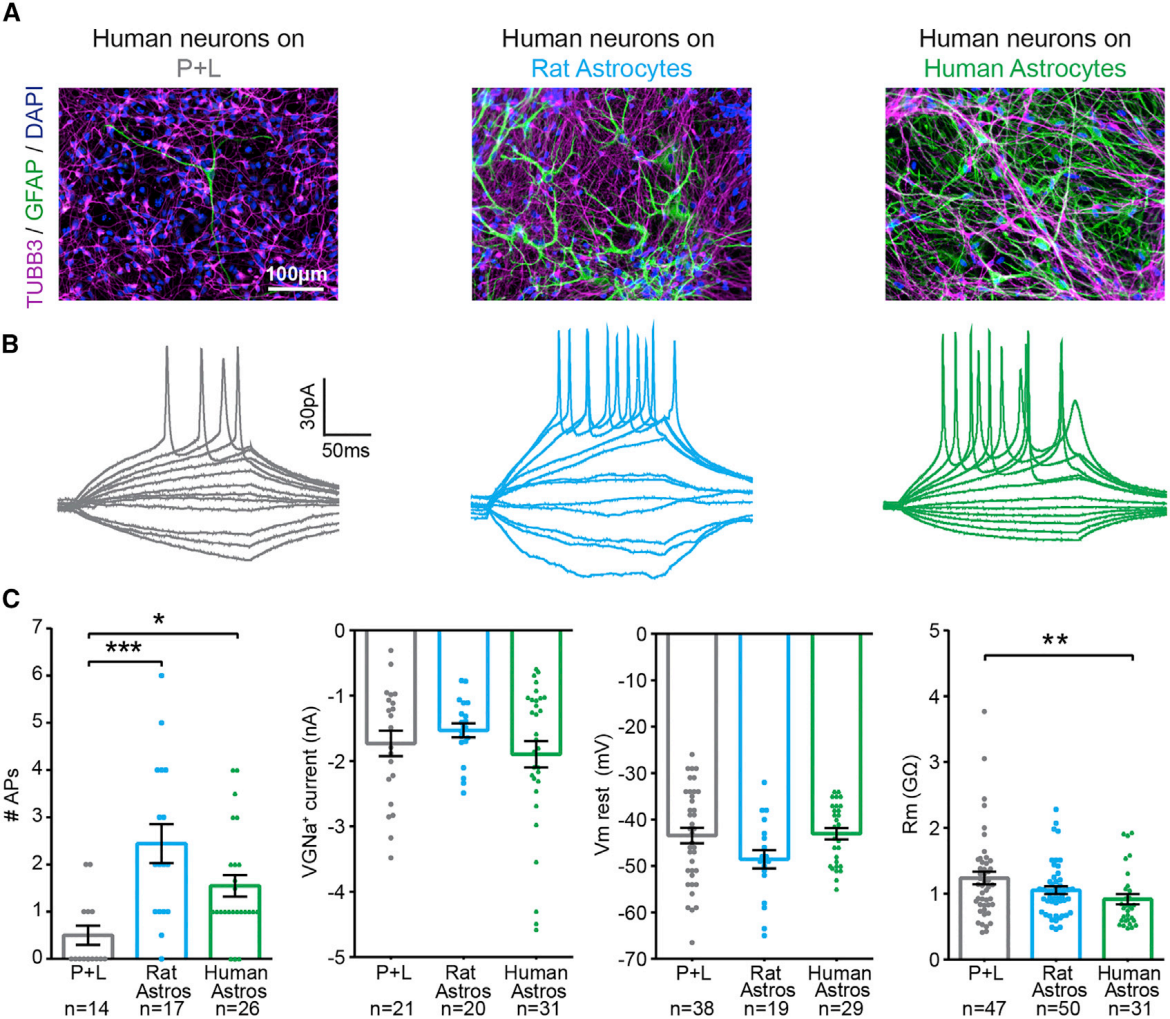
Co-culture with iPSC-Astrocytes Enhances Cortical iPSC-Neuron Excitability (A) Example immunofluorescence images of human iPSC-neurons grown in three different culture conditions: on polyornithine and laminin-coated coverslips (P + L, neurons 127 days; left), as co-cultures with rat astrocytes (neurons 79 days, co-cultured for 36 days; middle), or as co-cultures with human iPSC-astrocytes (neurons 88 days, astrocytes 117 days, co-cultured for 47 days; right). (B) Example action potentials evoked in response to square current steps across the three culture conditions. (C) Population data on the number of action potentials, amplitude of peak VGNa^+^ currents, resting membrane potential (Vm rest), and membrane resistance (Rm) across the three culture conditions. Sample sizes represent individual neurons recorded from 13 cultures across three cell lines (P + L neurons: 92 ± 3.3 days), 12 cultures across two cell lines (on rat astrocytes: neurons 93 ± 4.9 days, co-cultured for 51 ± 2.9 days), and five cultures across two cell lines (on human astrocytes: neurons 84 ± 7.6 days, astrocytes 120 ± 9.5 days, co-cultured for 44 ± 7.7 days). ^∗^p < 0.05, ^∗∗^p < 0.01, ^∗∗∗^p < 0.001.

In addition to regulating ongoing synaptic transmission, astrocytes also influence neuronal and synaptic maturity during development. When co-cultured with immature iPSC-neurons, human iPSC-astrocytes have been shown to enhance neuronal intrinsic excitability (Kayama et al., 2018, Klapper et al., 2019, VanderWall et al., 2019) and increase the number of synaptic puncta (Canals et al., 2018, Klapper et al., 2019, Krencik et al., 2011, Lischka et al., 2018, Serio et al., 2013, Shaltouki et al., 2013, Sloan et al., 2017, Tchieu et al., 2019, VanderWall et al., 2019). However, a limited number of studies have demonstrated functional maturation of synaptic networks (Canals et al., 2018, Tchieu et al., 2019, VanderWall et al., 2019), with one study reporting no maturational effects upon neuronal excitability or synaptic networks (Lischka et al., 2018). To examine whether our iPSC-astrocytes promote neuronal excitability and synaptic maturation, mKate^+^ iPSC-neurons were grown under one of the following three conditions: (1) on polyornithine and laminin (P + L), (2) as co-cultures on rat cortical astrocytes, or (3) as co-cultures on human iPSC-derived cortical astrocytes of the same donor origin (Figure 4A). Whole-cell patch-clamp recordings revealed that co-culture with either rat astrocytes or human iPSC-astrocytes significantly enhanced the propensity of the neurons to fire action potentials (0.5 ± 0.2 action potentials on P + L, 2.4 ± 0.4 action potentials on rat astrocytes; p = 0.0002, and 1.5 ± 0.2 action potentials on human astrocytes; p = 0.013, unpaired Kruskal-Wallis with Dunn's multiple comparisons test; Figures 4B and 4C). This increase in neuronal excitability was not associated with differences in the amplitude of VGNa^+^ currents (p = 0.877, unpaired Kruskall-Wallis test, Figure 4C), or resting membrane potentials (p = 0.121, unpaired Kruskall-Wallis test), between the three conditions. However, a decrease in membrane resistance was observed between the control (P + L) and the human iPSC-astrocyte co-culture condition (1.2 ± 0.1 GΩ on P + L, 1.1 ± 0.06 GΩ on rat astrocytes, and 0.92 ± 0.08 GΩ on human iPSC-astrocytes; p = 0.008, unpaired Kruskall-Wallis with Dunn's multiple comparisons test; Figure 4C), consistent with enhanced iPSC-neuron maturity (Bardy et al., 2016).

**Figure 5:**
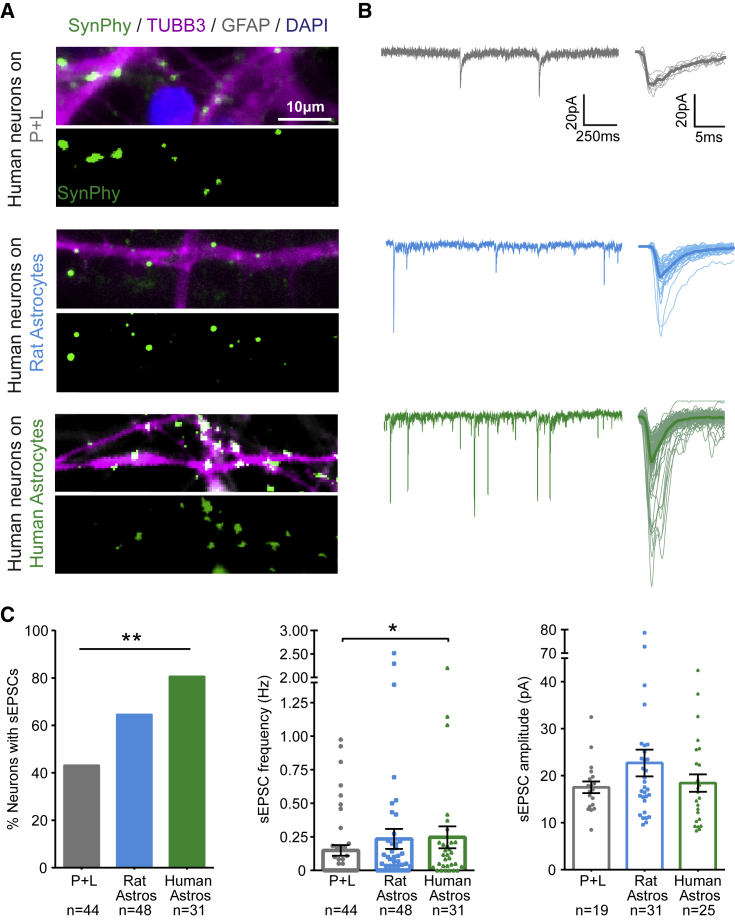
Co-culture with iPSC-Astrocytes Enhances Synaptic Networks among Cortical iPSC-Neurons (A) Example immunofluorescence images showing putative synaptophysin-positive (SynPhy^+^) pre-synaptic connections overlapping with TUBB3^+^ neuronal processes in the three culture conditions: on polyornithine and laminin-coated coverslips (P + L, neurons 73 days; top), as co-cultures with rat astrocytes (neurons 62 days, co-cultured for 22 days; middle), or as co-cultures with human iPSC-astrocytes (neurons 80 days, astrocytes 104 days, co-cultured for 37 days; bottom). (B) Example whole-cell patch-clamp recordings of sEPSCs across the three culture conditions. An individual trace is shown (left) and overlays of detected sEPSCs (right), with the average sEPSC in bold. (C) Population data showing the proportion of neurons receiving sEPSCs (left), the frequency of sEPSCs (middle), and amplitude of sEPSCs (right) across the three culture conditions. Sample sizes represent individual neurons recorded from 13 cultures across three cell lines (P + L neurons: 92 ± 3.3 days), 12 cultures across two cell lines (on rat astrocytes: neurons 93 ± 4.9 days, co-cultured for 51 ± 2.9 days), and five cultures across two cell lines (on human astrocytes: neurons 84 ± 7.6 days, astrocytes 120 ± 9.5 days, co-cultured for 44 ± 7.7 days). ^∗^p < 0.05, ^∗∗^p < 0.01.

To evaluate whether the iPSC-astrocytes could also influence synaptic network maturation, the presence of structural pre-synaptic sites was confirmed in each of the three culture conditions (Figure 5A), whereupon functional synaptic transmission was assessed by recording sEPSCs (Figure 5B). The proportion of neurons receiving synaptic inputs was significantly enhanced by co-culture with either rat astrocytes or human iPSC-astrocytes (43% on P + L, 65% on rat astrocytes, and 81% on human astrocytes; p = 0.004, chi-square contingency test; Figure 5C). The frequency of sEPSCs was enhanced by co-culture with human astrocytes (0.15 ± 0.04 Hz on P + L, 0.23 ± 0.07 Hz on rat astrocytes, and 0.25 ± 0.08 Hz on human astrocytes; p = 0.050, unpaired Kruskal-Wallis with Dunn's multiple comparisons test; Figure 5C). The amplitude of sEPSCs was similar between conditions (p = 0.470, unpaired Kruskall-Wallis test). Together, these data demonstrate that our cortically derived iPSC-astrocytes are capable of exhibiting pro-maturational effects upon cortically derived iPSC-neurons.

### Pro-maturational iPSC-Astrocytes Express Extracellular Proteins that Interact with Neuronal Synaptic Proteins

**Figure 6:**
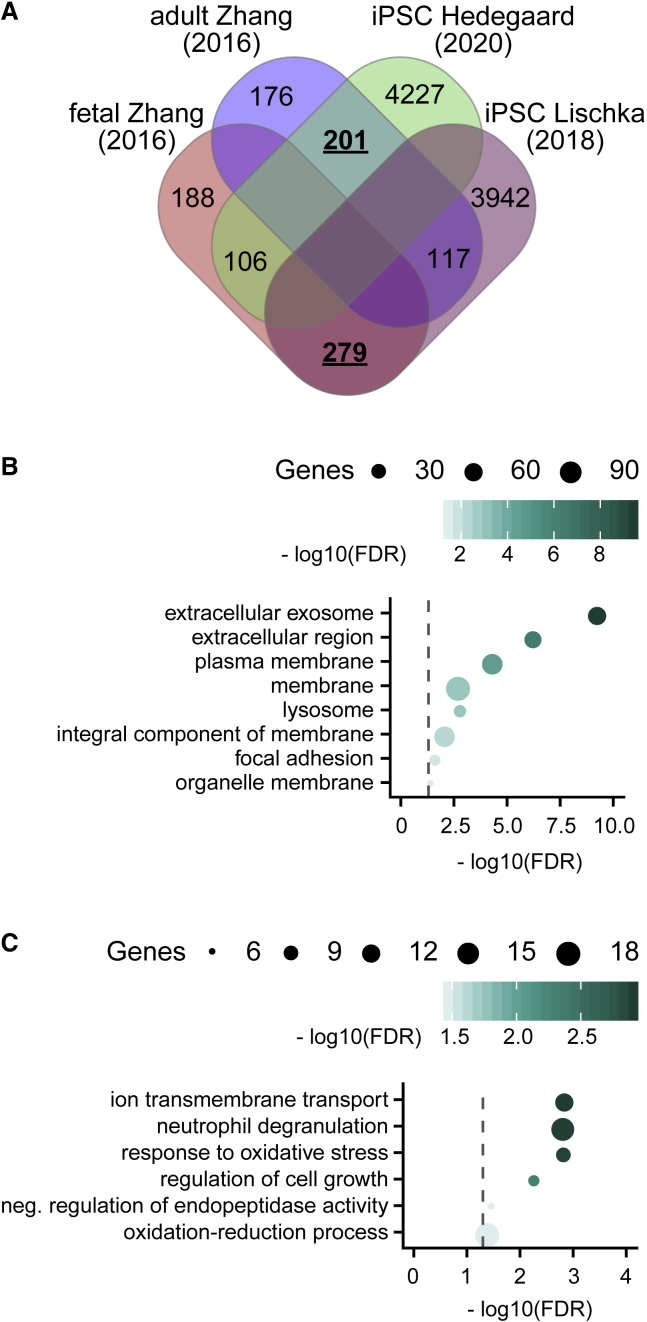
Transcriptomic Features of iPSC-Astrocytes that Mediate Pro-maturational Effects upon Cortical Neurons (A) Venn diagram showing overlap between genes that are highly expressed in either fetal and adult human astrocytes, identified in Zhang et al. (2016) and genes that were differentially expressed in the iPSC-astrocytes presented in this study (iPSC Hedegaard) or iPSC-astrocytes known to not promote neuronal maturation (iPSC Lischka). Overlapping gene sets that are larger than expected by chance are underlined and in bold (p < 0.05). (B and C) Overrepresented cellular components (B) and biological processes (C) among the set of 201 genes with higher expression in both primary adult astrocytes (compared with fetal) and iPSC Hedegaard astrocytes (compared with iPSC Lischka). Circle size indicates the number of genes annotated to each Gene Ontology (GO) term; color reflects the log10 transformed false discovery rate (FDR) and dashed line indicates a FDR of 0.05.

To investigate the biological processes underlying these pro-maturational effects upon neurons, we compared the differences in gene expression between our iPSC-astrocytes and iPSC-astrocytes previously shown to be unable to promote synaptic network maturation (Lischka et al., 2018) with those genes differently expressed between adult and fetal astrocytes from human brain tissue (Zhang et al., 2016; Figure 6A; DESeq2, false discovery rate [FDR] <0.05). Consistent with their pro-maturational effects, a statistically significant concordance was only found between (1) genes whose expression was higher in both the primary adult astrocytes and higher in our iPSC-astrocytes (n = 201 genes, p = 1.28 × 10^−9^, hypergeometric test), and (2) genes whose expression was higher in both the fetal astrocytes and higher in the Lischka et al. (2018) iPSC-astrocytes (n = 279 genes, p = 6.85 × 10^−30^, hypergeometric test). We did not find more differentially expressed genes than expected by chance in any other gene set overlaps (p > 0.05; Figure 6A). Among the 201 genes that were higher in both the primary adult astrocytes and our iPSC-astrocytes, we found an overrepresentation of proteins located extracellularly, in membranes and/or in lysosomal compartments (Figure 6B), with overrepresented roles including ion transmembrane transport, neutrophil degranulation, and response to oxidative stress (Figure 6C, hypergeometric test, FDR <0.05). Meanwhile, among the 279 genes higher in both the primary fetal astrocytes and the Lischka et al. (2018) iPSC-astrocytes, we found an overrepresentation of proteins associated with chromosomes, mitotic spindles, and the nucleoplasm, with overrepresented roles including cell cycle and cell proliferation processes (Figure S5, hypergeometric test, FDR <0.05).

**Figure 7:**
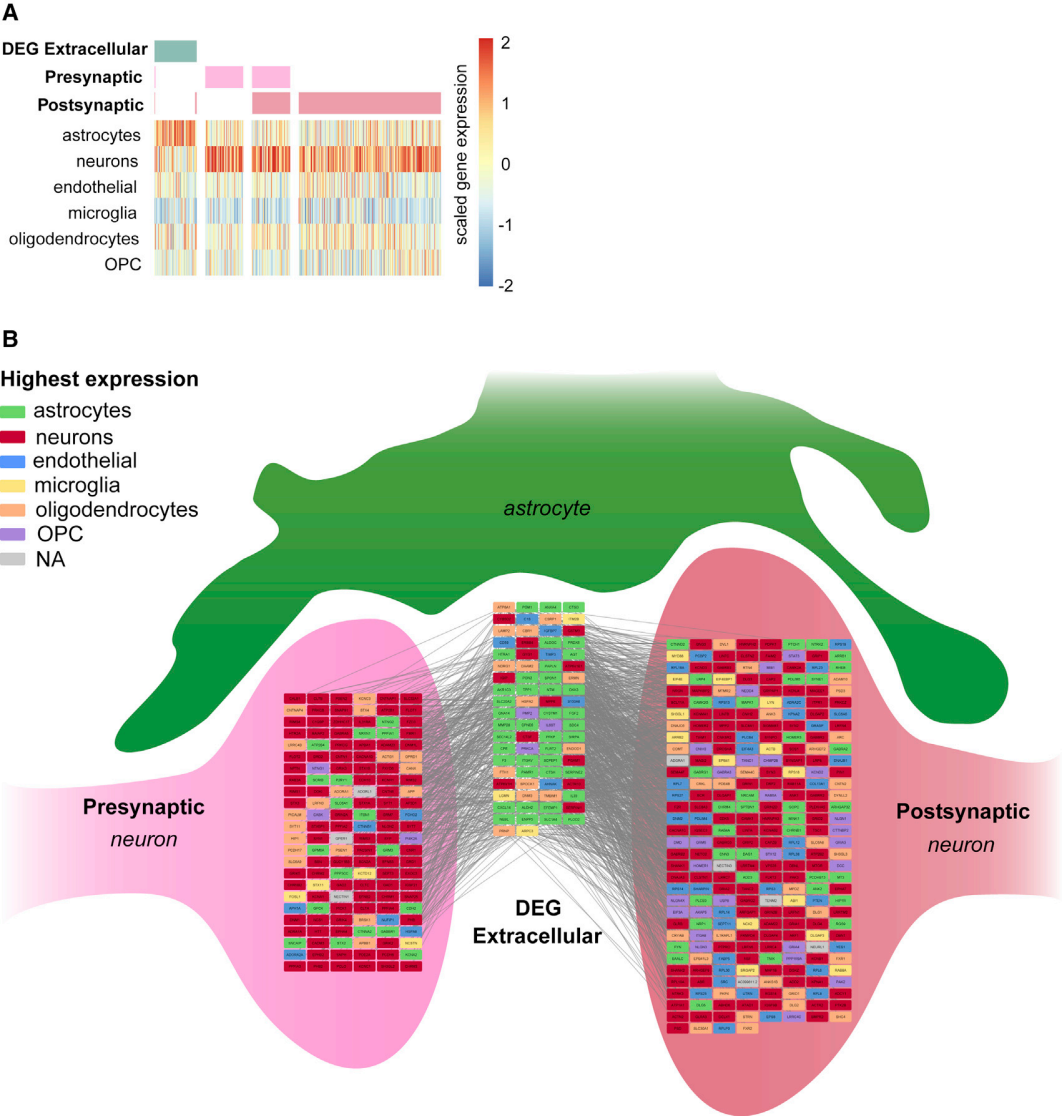
Pro-maturational Effects of iPSC-Astrocytes Are Associated with the Expression of Synapse-Interacting Extracellular Proteins (A) Of the 201 differentially expressed genes that overlap between primary mature astrocytes and the iPSC-astrocytes generated in this study, 84 encode extracellular proteins (annotated to GO:0070062, GO:0005576, or GO:0005615, shown in green). Genes encoding pre- and post-synaptic proteins extracted from annotated datasets (shown in light and dark pink, respectively). The expression patterns of extracellular differentially expressed genes (DEGs) and pre- and post-synaptic genes were corroborated across different brain cell types according to Darmanis et al. (2015). (B) Protein-protein interaction network for the differentially expressed extracellular astrocyte genes, and pre- and post-synaptic genes. Nodes represent genes and are color-coded according to the cell type with highest expression. Each line denotes a known protein-protein interaction between the protein products of these genes. See Figure S6 for a more detailed representation of the network. OPC, oligodendrocyte precursor cells.

Given the overrepresentation of genes associated with membrane and extracellular components in both the primary adult astrocytes and our iPSC-astrocytes, we hypothesized that these genes might be important for interactions with neurons and synapses. To explore their potential role, we extracted the subset of the 201 genes annotated as encoding extracellular proteins (n = 84 genes) and confirmed, using a human atlas of gene expression patterns, that these exhibit highest expression in mature astrocytes (Darmanis et al., 2015; Figure 7A). Using Gene Ontology (GO) annotations, we then identified all genes whose proteins are annotated as “pre-synaptic” and “post-synaptic” and corroborated their overall neuron-specific expression (Darmanis et al., 2015; Figure 7A). Finally, we asked if the identified subset of astrocytic extracellular proteins are predicted to engage in protein-protein interactions with pre- and post-synaptic neuronal proteins (see Supplemental Experimental Procedures). This revealed a statistical overrepresentation of interactions between the astrocytic extracellular proteins and both the neuronal pre-synaptic and post-synaptic proteins (p = 0.0027 and p = 0.0055, respectively; via randomizations; Figure 7B; expanded subset of the protein-protein interaction network is shown in Figure S6). Taken together, these transcriptomic analyses support the idea that the iPSC-astrocytes can engage in synapse-associated extracellular signaling to mediate pro-maturational effects upon neurons.

## Discussion

Here, we establish a human cortical iPSC system for investigating astrocyte-to-neuron interactions. Our approach uses dual SMAD inhibition to generate a common pool of OTX2^+^ cortical radial glia progenitors, from which both astrocytes and neurons could then be independently generated. This strategy reflects *in vivo* cortical development, where neurons and astrocytes derive from a common progenitor pool (Rowitch and Kriegstein, 2010), and aligns with the idea that the regional identity of a progenitor influences the identity of its progeny (Nadadhur et al., 2018). The resulting method afforded the opportunity to examine astrocyte-neuron interactions in cells from a defined, common lineage. As with iPSC-astrocytes generated via other protocols, transcriptomic analyses indicated that our cortical iPSC-astrocytes are relatively immature compared with adult cortical astrocytes from human brain (Lin et al., 2018, Lischka et al., 2018, Santos et al., 2017, Tcw et al., 2017, Zhang et al., 2016). Nevertheless, at the stages examined, the cortical iPSC-astrocytes exhibited key molecular and functional features, including the expression of well-known astrocyte-specific genes, relevant intrinsic membrane properties, gap-junction coupling, and mechanisms for regulating extracellular molecules.

A major focus was to examine the capacity of the iPSC-astrocytes to engage in intercellular signaling with iPSC-derived neurons, because the importance of these interactions is being increasingly recognized in development and in disorders such as epilepsy and Alzheimer's disease (Dossi et al., 2018, Wetherington et al., 2008). Our cortically derived iPSC-astrocytes exhibited rapid increases in intracellular Ca^2+^ in response to neurotransmitters such as glutamate and ATP, as has been shown for other populations of iPSC-astrocytes (Canals et al., 2018, Krencik et al., 2011, Santos et al., 2017, Tchieu et al., 2019, Tcw et al., 2017). We have demonstrated that the iPSC-astrocytes are also able to rapidly signal back to neurons. Brief and selective optogenetic stimulation of the iPSC-astrocytes resulted in the modulation of ongoing synaptic transmission between nearby neurons, in a mGluR-dependent manner. This is analogous to forms of activity-dependent gliotransmission described in rodent cortex and in biopsies from human cortex (Navarrete et al., 2013, Perea et al., 2014, Sasaki et al., 2012). Although it is widely accepted that astrocytes engage in activity-dependent signaling, there is debate regarding the conditions under which gliotransmission occurs and the underlying cellular mechanisms (Fiacco and McCarthy, 2018, Savtchouk and Volterra, 2018). The current study therefore provides a human system in which to examine these processes, plus the opportunity to explore this in a disease context. Our data derived primarily from three healthy donors, but also included a small number of astrocyte cultures from a cell line derived from a donor diagnosed with sporadic Alzheimer's disease (see Supplemental Experimental Procedures). In the future, it would be interesting to examine how astrocyte-to-neuron signaling relates to the state of the human astrocyte, the developmental stage of the network, and to neurological disorders such as Alzheimer's disease.

Astrocyte-to-neuron interactions were also captured over longer timescales. Our long-term co-culture experiments revealed pro-maturational effects of cortical iPSC-astrocytes upon cortical iPSC-neurons. The neurons exhibited enhanced electrical excitability and synaptic network activity, equivalent to the effects observed following co-culture with rodent cortical astrocytes. These findings extend recent reports that iPSC-astrocytes of different origin can exhibit certain pro-maturational effects upon co-cultured neurons (Canals et al., 2018, Kayama et al., 2018, Klapper et al., 2019, Tchieu et al., 2019, VanderWall et al., 2019), but can fail to exhibit such effects (Lischka et al., 2018). By combining our observations with transcriptomic analyses, we linked the pro-maturational effects on neuron to astrocyte maturity and the expression of astrocytic genes encoding extracellular proteins. More specifically, our approach identified candidate genes and pathways relevant to extracellular interactions with neuronal pre- and post-synaptic proteins. These included: ITGAV, an essential protein for neuron-glial attachment during cortical development, which influences synapse structure and maturation (Anton et al., 1999, Park and Goda, 2016); SLC1A4, a Na^+^-dependent amino acid transporter that plays a role in modulating glutamatergic transmission (Kaplan et al., 2018); NTM, ENPP5, EFEMP1, and SIRPA, all glycoproteins involved in cell adhesion, cell-to-cell recognition, and EFEMP1 and SIRPA have been associated with neurite outgrowth and synaptic development (Vukovic et al., 2009, Wang and Pfenninger, 2006). Future work could use targeted manipulations to investigate the relevance of these astrocytic genes in terms of the formation and maintenance of human cortical neuronal networks.

In conclusion, we provide a detailed description of human cortical iPSC-derived astrocytes, which can reproduce many of the key astrocyte-to-neuron signaling processes observed in animal systems. The iPSC-astrocytes are able to signal bidirectionally with iPSC-neurons, by rapidly responding to neurotransmitters and actively modulating ongoing neuronal activity. Interactions over longer timescales result in pro-maturational effects upon cortical neuronal networks, which are associated with synapse-related signaling between the astrocytes and neurons. This work provides a foundation for further investigations into astrocyte-neuron interactions in human health and disease.

## Experimental Procedures

### Human iPSC Lines

The iPSC lines were derived from human skin biopsy fibroblasts following signed informed consent, with approval from the UK NHS Research Ethics Committee (REC, 13/SC/0179) and were derived as part of the IMI-EU sponsored StemBANCC consortium. Further information in Supplemental Experimental Procedures.

### Differentiation of iPSCs to Cortical Neurons and Astrocytes

iPSCs were differentiated into cortical neurons using the protocol detailed in Volpato et al. (2018) (“Standard operating procedure for cortical differentiation of hiPSCs”) and based on Shi et al. (2012). Human astrocytes were differentiated from cortical progenitors using the Astrocyte Differentiation and Maturation kits available from STEMCELL Technologies (nos. 08540 and 08550, respectively). In brief, cortical rosettes at day 20–25 of neural induction were treated with the Astrocyte Differentiation medium for 20 days (until day 40–45), during which cells were expanded three times using Accutase (Sigma), and re-plated at a density of 1 × 10^5^ cells/cm^2^ on Matrigel-coated six-well plates. Subsequently the medium was switched to the Astrocyte Maturation medium for a further 15 days (until day 55–60), and cells were expanded three times as described above. At the transition stage between different media, cultures were cryopreserved in their current medium + 10% DMSO. To maintain astrocytes beyond day 60, cells were cultured in an astrocyte maintenance medium, which was replaced every 2–3 days. Further details in Supplemental Experimental Procedures.

### Establishment of Co-cultures

For co-cultures of human iPSC-neurons with either human or rat astrocytes, the astrocytes were seeded onto Matrigel-coated (Scientific Lab Supplies) glass coverslips and allowed to reach confluence, before 100,000–150,000 cortical neurons/coverslip were seeded on top. To facilitate selective patching of neurons in co-cultures, iPSC-neurons were transduced with a lentivirus expressing mKate2 under the control of the CamKIIα promoter (see Supplemental Experimental Procedures) prior to co-culture. At the time of co-culture, human astrocytes were typically ∼70–80 days old and neurons were ∼35–45 days old, and co-cultures were always generated with cells from the same donor. For optogenetic experiments, astrocytes were transduced with a lentivirus expressing humanized ChR2 (hChR2) under the control of a CAG promoter (see Supplemental Experimental Procedures) before the addition of neurons. Co-cultures were maintained in a modified neuronal maintenance medium, containing (50% vol/vol Neurobasal-A, 50% vol/vol DMEM/F12 Glutamax medium with 1× N2, 1× B27 + vitamin A, 2.5 μg/mL insulin, 1 mM L-glutamine, 0.5× non-essential amino acids, 0.5 mM sodium pyruvate, 55 μM β-mercaptoethanol, 50 U/mL penicillin, and 50 mg/mL streptomycin), with medium changes every 2–3 days and supplemented with 2% fetal bovine serum and laminin at 10 μg/mL once a week.

### Electrophysiological Recordings

Whole-cell patch-clamp recordings were performed with thin-wall borosilicate glass pipettes (resistances of 5–8 MΩ for neurons and 7–12 MΩ for astrocytes), back-filled with intracellular solution (140 mM K^+^ gluconate, 6 mM NaCl, 1 mM EGTA, 10 mM HEPES, 4 mM MgATP, and 0.4 mM Na_3_GTP). For biocytin filling, 5 μg/mL biocytin (Sigma) was dissolved in the internal solution and left to disperse for at least 15 min. During recordings, the cultures were constantly perfused with external solution (140 mM NaCl, 5 mM KCl, 2 mM CaCl_2_, 10 mM HEPES, and 10 mM glucose) at a rate of 2 mL/min, heated to 33–35°C. Voltage-gated currents were elicited from cells clamped at −70 mV in voltage-clamp mode, using 10 mV voltage step protocols from −90 to +20 mV (neurons) or −140 to +30 mV (astrocytes). Action potentials were elicited in current-clamp mode by the injection of 200 ms square current pulses (5 or 10 pA steps), from a baseline Vm of −70 mV and were counted if their peak was greater than −10 mV. sEPSCs were recorded in voltage-clamp mode at the reversal potential of GABA_A_ receptors (E_GABA_; −70 mV after +14mV junction potential correction). For further details of electrophysiological data analysis, see Supplemental Experimental Procedures.

### Optogenetic Stimulation

Stimulation of ChR2-expressing astrocytes and simultaneous patch-clamp recordings of sEPSCs from co-cultured neurons were performed using a Rebel Star Royal Blue LED (447.5 nm wavelength, Luxeon). These neurons were clamped at −70 mV in the presence of picrotoxin (PTX). A 2 min baseline was recorded, then a 50 s train of 245 ms LED pulses was delivered at 2–4 Hz from beneath the culture. LED intensities ranged from 0.3 to 2.7 mW/mm^2^.

### RNA Sequencing and Quantification

Nine samples of iPSC-astrocytes (three cultures from each of three cell lines; mean age, 96 ± 3.3 days) were prepared for sequencing using the HiSeq 3000/4000 SBS Kit. Approximately 30 million 75 bp paired-end reads were obtained per sample. For initial quality control, FASTQC (Andrews, 2010) and MultiQC were used to summarize the results (Ewels et al., 2016). Kallisto for Linux version 0.43.1 (Bray et al., 2016) was used to quantify transcript abundances. For further details on differential expression analysis, GO analysis and protein-protein interaction analysis, see Supplemental Experimental Procedures.

### Experimental Design and Statistical Analysis

All data were collected and analyzed by the same person (A.H.). All statistical analysis was performed using Prism software, version 6 (GraphPad). Bars on graphs represent means and error bars indicate ± standard error of the mean. Non-parametric statistical tests were used on all datasets, because ranks and medians are more robust to outliers. The number of cells, fields of view, and regions of interest evaluated in each experiment are reported in the figures and the legends. Datasets were regarded as unpaired, and two-tailed tests were performed. The relevant statistical tests, as well as corrections for multiple comparisons, are indicated after each reported p value. Statistical significance is reported at the following levels: ^∗^p < 0.05, ^∗∗^p < 0.01, ^∗∗∗^p < 0.001 in the figures, with precise p values given in the text.

### Accession Numbers

The accession number for the transcriptomic datasets reported in this paper is GEO: GSE149598.

## Author Contributions

Designed research: A.H., S.E.N., C.W., and C.J.A. designed the research. A.H. and J.M.-S. performed the research. E.S.W. and S.E.N. contributed unpublished reagents/analytical tools. A.H. and J.M.-S. analyzed the data. A.H., J.M.-S., and C.J.A. wrote the paper. The manuscript was approved by all of the authors.

## Supplemental Information

Document S1. Supplemental Experimental Procedures and Figures S1–S6

Document S2. Article plus Supplemental Information
