# Supplementary material for: Pro-maturational Effects of Human iPSC-Derived Cortical Astrocytes upon iPSC-Derived Cortical Neurons
Source: Stem Cell Reports. 2020 Jun 4;15(1):38–51. doi: 10.1016/j.stemcr.2020.05.003 (PMC7363746; doi:10.1016/j.stemcr.2020.05.003)
Supplement: Document S1. Supplemental Experimental Procedures and Figures S1–S6 [file mmc1.pdf]

Stem Cell Reports, Volume *15*

Supplemental Information

Pro-maturational Effects of Human iPSC-Derived Cortical Astrocytes upon iPSC-Derived Cortical Neurons

Anne Hedegaard, Jimena Monzón-Sandoval, Sarah E. Newey, Emma S. Whiteley, Caleb Webber, and Colin J. Akerman

Supplemental experimental procedures

Maintenance of human iPSC lines

iPSC lines were reprogrammed using the CytoTune-iPS 2.0 Sendai Reprogramming Kit (A16517, Thermo Fisher Scientific; (Volpato et al., 2018)). The vast majority of data was collected from healthy control cell lines: SBAd03, Clones# 01 and 05, female aged 31 years; SBAd02, Clone# 01, male aged 51 years; SFC841-03, Clone# 01, male aged 36 years. In assessing the potential for our protocol to differentiate iPSCs to astrocytes, some differentiations were also performed using a cell line from a patient diagnosed with sporadic Alzheimer's disease (AD): SFC042-03, Clone# 01, female aged 67 years. Whilst we had limited statistical power to compare across cell lines, we did not observe any difference in the ability of this cell line to differentiate into astrocytes. This line contributed 4 data points to the plots in Figure 2C, 2 data points in Figure 2G, 3 data points in Figure S3 and one field of view in Figure 3A. The human iPSCs were maintained under feeder-free conditions on geltrex-coated (Gibco) six-well plates (Corning) in mTeSR1 (STEMCELL Technologies) and passaged at a rate of 1:3 with enzymatic dissociation using Versene-EDTA when they reached 90% confluency.

Differentiation of iPSCs to cortical neurons

In preparation for neural induction, following a modified Shi et al. (2012) protocol (Shi et al., 2012), the iPSCs were passaged using Versene-EDTA at a ratio of 2:1 to ensure 100% confluency. The next day (Induction day 0), the culture medium was changed to neural induction medium (NIM) consisting of neural maintenance media (NMM: 50% vol/vol Neurobasal, 50% vol/vol DMEM/F12 Glutamax medium with 1x N2, 1x B27 + Vitamin A, 2.5 µg/mL insulin (Sigma), 1 mM L-glutamine, 0.5x non-essential amino acids (NEAAs), 0.5 mM Sodium Pyruvate (Sigma), 55 µM β-mercaptoethanol, 50 U/mL penicillin and 50 mg/mL streptomycin), supplemented with dual SMAD inhibiting factors: 10 µM SB431542 (Tocris) and 1 µM dorsomorphin (Tocris). NIM was changed daily for 12 days as the cells were forming a dense neuroepithelial sheet. To lift the sheet on day 13, it was scored into a grid using a sterile needle and incubated at 37°C with 200 µL freshly filtered 10 mg/mL Dispase (Gibco) added to the media. Lifting was encouraged by frequently tapping the plates over 10-15 min of incubation and cells were collected using a stripette to gently transfer them without breaking up the sheet too much into 10 mL of NMM. To wash out the Dispase, cells were allowed to pellet by gravity, media removed and this wash-step repeated. Cell clumps were plated in NIM supplemented with 10 µM Y-27632 (Tocris) onto plates pre-coated for >4 hours with 15 µg/mL laminin (Life Technologies) and left to adhere overnight. The medium was changed 24 hours later to NMM supplemented with 20 ng/mL FGF2 (Peprotech) and changed every day for 4 days to promote/encourage rosette formation.

Rosettes were then maintained in NMM over the next 10-15 days, during which they were expanded onto laminin-coated wells and cleaned up 2-5 times by selective lifting with Dispase as described above, but without the needle-scoring. Onset of neurogenesis occurred around day 20-25, at which point cultures were dissociated to single cells using Accutase (Sigma) and plated onto laminin-coated wells at a ratio of 1:2 in the following manner: cells were washed with PBS and subsequently incubated with 500 µL Accutase solution at 37°C for 5-10 min. To loosen all cells and split up clumps, gentle trituration and collection of cells into 9 mL NMM was performed using a P1000. This cell-suspension was centrifuged at 300 g for 5 min (Heraeus Megafuge 8 centrifuge (Thermo Scientific)), the supernatant removed and the pellet re-suspended in NMM with 10 µM Y-27632 for plating. The next day, cells were given fresh NMM. The resulting neural progenitor cells (NPCs) were generally expanded a second time using Accutase, before being frozen down between days 28-32 post initiation of neural induction. Freezing involved lifting with Accutase, and re-suspending the centrifuged cell-pellet in ice-cold freezing media consisting of: 90% vol/vol NMM, 10% vol/vol dimethylsulfoxide (DMSO) (Sigma) and 20 ng/mL FGF2 (Peprotech), quickly aliquoting in cryovials at $1.5x10^6$ cells/vial and storing them overnight in a CoolCell freezing container (Corning) at -80°C to facilitate slow freezing, and moving them to liquid nitrogen (LiN_2) storage the following day.

Differentiation of iPSCs to cortical astrocytes

The protocol accompanying the astrocyte differentiation and maturation kits from STEMCELL technologies was adapted to start from a neural rosette stage. Neural rosettes were generated as described above and grown on laminin and the AstroDiff media was applied around day 20-25 of neural induction. Astrocyte progenitors were kept in AstroDiff for 20 days (until day 40-45) and underwent 3 expansions, every time when cells reached ~90% confluency. Cells were lifted with Accutase (Sigma), washed in AMM, centrifuged at 300 g for 5 min and re-suspended in AstroDiff for re-plating at $1x10^5$ cells/cm^2. At the 3rd passage, the majority of cells were frozen down as progenitor stock in 90% vol/vol AstroDiff media with 10% vol/vol DMSO (Sigma). Media was then changed to AstroMature for a further 15 days (until day 55-60), during which astrocyte precursors also underwent 3 passages with Accutase.

Similarly, at the 6th passage, cells could be frozen down as astrocyte precursor stock in 90% vol/vol AstroMature media with 10% vol/vol DMSO. Maintaining the astrocytes beyond the manufacturer's protocol was done in astrocyte maintenance (AMM: 45% vol/vol Neurobasal, 45% vol/vol DMEM/F12 Glutamax containing 1 mM L-glutamine, 50 U/mL penicillin and 50 mg/mL streptomycin and 10% filtered FBS***)***. Final maturation was thus conducted in AMM media for 20-30 days (until day 75-90) and if cells were still proliferating, further passages were conducted, but using Trypsin/EDTA (Gibco) and washing in serum-containing AMM. Media was changed every 2-3 days throughout the differentiation protocol. Cryo-stored stocks of astrocyte progenitors and precursors could be thawed and the protocol resumed from the point they were frozen down.

Generation and maintenance of cortical rat astrocytes

Cortical rat astrocytes were either obtained from Gibco and grown in glial medium (DMEM with 15% vol/vol filtered FBS), or generated from P1 rat cortices (Wistar rats; Charles River Laboratories) according to (Kaech and Banker, 2006), and in accordance with the Animals (Scientific Procedures) Act, 1986 (United Kingdom). For experimentation, rat astrocytes were seeded onto 13 mm acid-washed and sterilized glass coverslips, coated with 0.1 mg/mL poly-D-lysine (Sigma), and grown until confluent.

Lentiviral vectors

Vector	Virus	Source	Identifier	Reference
**pLenti-CAG-hChR2(H134R)-EYFP-WPRE**	LV-CAG-ChR2-YFP	A gift from Karl Deisseroth	Addgene plasmid # 20945<br>RRID: Addgene_20945	(Zhang et al., 2007)
**pLenti-CaMKIIa-mKate2-WPRE**	LV-CamKIIa-mKate2	A gift from Ricardo Dolmetsch and Oleksandr Shcheglovitov	Addgene plasmid # 96942<br>RRID: Addgene_96942	(Shcheglovitov et al., 2013)

HEK293T cells for transfection were plated in a T75 flask (Corning) at a density of $6.5x10^6$ cells/flask in growth medium consisting of: 90% vol/vol DMEM (Gibco) and 10% vol/vol FBS (Gibco). Cells were grown to 60% confluency and media supplemented with 1x NEAAs (Gibco) prior to transfection. Cells were transfected according to the protocol by (Tashiro et al., 2015) with 100 µL Lipofectamine 2000 (Invitrogen), 9.375 µg vector DNA, 3.125 µg VSVg envelope DNA and 8.75 µg Δ8.91 packaging DNA made up in Opti-MEM (Gibco) in the following way: Lipofectamine was added to 1 mL Opti-MEM and incubated at room temperature for 5 min. Separately, DNA was added to 1 mL Opti-MEM and then transferred to the Lipofectamine solution, inverting to mix solutions. The mixture was incubated for a further 25-30 minutes before addition to HEK cells. Transfection medium was replaced with fresh growth medium 5 hours later.

Media containing the viral particles was harvested after 48 hours, centrifuged at 1000 g for 3 minutes to remove cell debris, and then passed through a 0.45 µm filter before aliquoting. If cells had not detached significantly, a second harvest could be obtained by adding media and collecting it 48 hours later. Supernatant was either stored at -80 ^oC or further processed by ultracentrifugation to concentrate the viral particles. For ultracentrifugation, 8 mL supernatant was transferred into conical bottomed, heat sealed tubes (Beckman Coulter) and ultracentrifuged at 30,000 rpm in a SW41TI swinging bucket rotor at 4°C for 3 hours (Beckman L8-70M Ultracentrifuge). The supernatant was subsequently extracted and each viral pellet was re-suspended in

20 µL Opti-MEM with 1:100 DNase I (1 U/µL stock concentration, Sigma), left overnight at 4 ^oC, and lastly aliquoted and stored at -80 ^oC.

Immunocytochemistry

Cells were pre-fixed in 2% PFA for 10 min, followed by fixation in 4% PFA for 10 min, washed three times in PBS, then permeabilized with 0.3% vol/vol Triton-X (TX) for 15 min, and blocked in PBS with 1.5% vol/vol Triton X-100 and 10% normal goat serum (NGS, Sigma) for 1h. Primary antibodies (see table below) were diluted in PBS with 0.15% Triton X-100 and 2% NGS and incubated with cells overnight at 4°C (long incubation of 48h used for synaptic antibodies). The next day, cells were washed three times in PBS and incubated with the relevant Alexa Fluor conjugated secondary antibodies (see table below) for 1h at room temperature (or overnight at 4°C for synaptic antibodies). Cells were washed three times in PBS, including one wash containing 1:5000 DAPI (D1306 ThermoFisher Scientific; RRID:AB_2629482), and finally mounted with ProLong Gold (ThermoFisher Scientific). Images were collected on an epifluorescence microscope (BX40, Olympus) and quantification of GFAP, S100β and Tuj1 positive cells was performed using the Cell-counter plug-in for Image J (Fiji).

Primary antibodies

Antibody	Raised in	Concen-tration	Supplier	Catalogue #	RRID
**GFAP**	Mouse IgG1	1:1000	Millipore	MAB360	AB_11212597
**Ki67**	Mouse IgG1	1:600	BD bioscience	550609	AB_393778
**MAP2A**	Mouse IgG1	1:1000	Millipore	MAB378	AB_11214935
**Otx2**	Rabbit	1:500	Millipore	ab9566	AB_2157186
**Pax6**	Rabbit	1:500	Covance	PRB-278P	AB_291612
**Synapto-physin**	Guinea Pig	1:1000	Synaptic Systems	101004	AB_1210382
**S100β**	Rabbit	1:800	Abcam	ab868	AB_306716
**Tuj1**	Mouse IgG2a	1:2000	Biolegend	B199846	AB_2313773

Secondary antibodies

Antibody	Conjugate	Raised in	Concen-tration	Supplier	Catalogue #	RRID
**Anti-mouse**	Alexa Fluor 488	Goat	1:1000	Life Tech.	A11029	AB_2534088
**Anti-mouse IgG1**	Alexa Fluor 488	Goat	1:500	Life Tech.	A21121	AB_2535764
**Anti-mouse**	Alexa Fluor 568	Goat	1:1000	Life Tech.	A11031	AB_144696
**Anti-mouse IgG2a**	Alexa Fluor 568	Goat	1:500	Life Tech.	A21134	AB_2535773
**Anti-mouse**	Alexa Fluor 633	Goat	1:500	Life Tech.	A21052	AB_2535719
**Anti-mouse IgG1**	Alexa Fluor 680	Goat	1:500	Life Tech.	A31562	AB_2536176
**Anti-rabbit**	Alexa Fluor 488	Goat	1:1000	Life Tech.	A11034	AB_2576217
**Anti-rabbit**	Alexa Fluor 568	Goat	1:1000	Life Tech.	A11036	AB_10563566
**Anti-guinea pig**	Alexa Fluor 488	Goat	1:500	Life Tech.	A11073	AB_2534117
**Anti-rat**	Alexa Fluor 488	Goat	1:1000	Life Tech.	A11006	AB_2534074

Measuring glutamate uptake currents

Experiments for detecting glutamate uptake with whole-cell patch clamp recordings followed previous work, and used the fact that the net ionic flux of astrocytic glutamate transporters is positive, and so can be detected as an inward current (Dallas et al., 2007). L-glutamate was first diluted in NaOH to 100 mM, then further diluted to a stock concentration of 10 mM in dH_2O, and used at a final concentration of 100 µM by dilution in ACSF. The biophysical properties of cells were first confirmed to correspond to putative astrocytes in terms of low $VGNa^+$ currents and lack of action potential initiation. Astrocytes were held in voltage clamp mode at -70 mV throughout a baseline period in normal ACSF, and then during wash-in of glutamate-containing ACSF for 1-

1.5 min. To determine if an astrocyte exhibited an inward current associated with glutamate uptake, 10 equally-spaced current measurements during the baseline period were compared to 10 equally-spaced current measurements in the glutamate-containing ACSF (Wilcoxon signed-rank test, $p<0.05$). The amplitude of the glutamate-associated uptake current was defined as the peak current shift in glutamate-ACSF, compared to the mean current during a 1 min baseline period in normal ACSF.

Glutamate uptake assay

An enzymatic glutamate detection kit (Sigma, MAK004) was used to assess glutamate uptake by iPSC-astrocytes. As astrocytic glutamate uptake is a Na^+-dependent process, the degree of glutamate uptake was determined by comparing cultures in Na^+-containing media, to those in Na^+-free media. Cells were first grown to confluency in 6-well plates and equilibrated with HBSS for 10 min, before the addition of 50 µM L-glutamate diluted in either normal HBSS or Na^+-free HBSS. Media samples were collected after 60 minutes and kept on ice. Cells were lifted with trypsin/EDTA, washed, pelleted and subsequently lysed in 500 µL ice-cold RIPA buffer (Thermo Fisher Scientific) containing phosphatase and proteinase inhibitors (Sigma). Cell debris was removed by centrifuging the lysate at 13,500 RPM for 5 min at 4°C. The enzymatic assay was conducted in 96-well plate format, by adding 100 µL reaction mix to each 50 µL media sample or diluted glutamate standard. The plate was incubated for 30 min at 37°C, protected from light and then the absorbance was measured at 450 nm on a Sunrise F039300 plate-reader (Tecan). All values were normalized to protein content, determined by running an accompanying BCA protein detection assay on the cell lysates (Thermo Scientific, 23227). The amount of glutamate uptake by the iPSC-astrocytes was calculated by subtracting the glutamate concentration remaining in the Na^+-containing media, from the glutamate concentration remaining in the Na^+-free media. Results are expressed as glutamate uptake by astrocytes above the Na^+-free condition, per amount of protein, per minute.

Calcium imaging

Cells were viewed using a BX51WI microscope equipped with a 20x/1.00NA XLUMPlanFL-N objective (Olympus) and iXon camera (Andor Technologies), using a 480/40 nm excitation filter, 505 nm dichroic and 520 nm emission filter. Astrocytes were loaded with 10 µM OGB-1 AM ester (Life Technologies, 06807), mixed from equal amounts of dye and Pluronic F-127 (20% solution in DMSO, Invitrogen), before being diluted and vortexed in AMM. The final mixture of dye, pluronic and media was added to the astrocytes and incubated for 60 min at 37°C. Following loading, dye-containing media was removed and cells incubated in normal media for 30 min and subsequently transferred to the recording chamber and continuously perfused with external solution (in mM: 140 NaCl, 5 KCl, 2 CaCl2, 10 HEPES and 10 glucose). The size of Ca^{2+} events was calculated as ΔF/F according to: $\frac{\Delta F}{F} = \frac{F_t - F_{t=0}}{F_{t=0}}$, where $F_{t=0}$ was the mean of at least the first 5 frames of a movie. Events were detected with a ΔF/F threshold of 1.05. Spontaneous events were detected from 1-2 min movies (2-4 Hz frame rate) and classified as synchronous if they initiated within 1 s of one another. Neurotransmitter-evoked events were studied by delivering focal puffs of agonist via a patch-pipette (containing 100 µM glutamate or 1 mM ATP in external solution), controlled by a micro-manipulator (Sutter Instrument Company) and attached to Picospritzer II (General Valve Corporation; 1-2 psi). Brightness and contrast was adjusted on all imaging files in Image J, along with histogram correction via the plugin from EMBL tools, Bleach correction. ROIs were placed over cell bodies or cell-free background areas. Data from all ROIs was extracted as mean grey values from every image via the Multi-measure function.

Data analysis of electrophysiological recordings

Electrophysiological data was acquired with a Multiclamp 700B amplifier and the accompanying software Clampex (version 10.5, Molecular Devices). Signals were digitized with an Axon Digidata 1550 (Molecular devices), filtered at 2 kHz (Bessel filter) and sampled at 20 kHz. Data analysis was conducted with Clampfit software (version 10.5, Molecular Devices). Automated measurements of access resistance (Ra), membrane capacitance (Cm) and membrane resistance (Rm) were logged through the Clampex membrane test function. To be included for analysis, cells required Ra values below 50 MΩ, and the Ra, Cm and Rm values before and after each recording could not deviate by more than +/- 20% from the mean. Voltage gated currents: The leak subtracted trace was used for analysis of sodium (Na^+) currents, for which the largest negative VG current was reported. Potassium (K^+) current behaviours were assessed from the non-leak subtracted traces and were classified based on their current-voltage (I-V) profiles. Automated sEPSC event detection was performed with the template fitting function in Clampfit. Subsequent post-hoc filtering of putative sEPSC events used criteria for amplitude, half width and rise time (>7 pA, <4 ms and <1.2 ms, respectively), designed to exclude false-positive events and validated through comparison against manual detection. Cells with a sEPSC frequency of <0.017 Hz (i.e. less than 1 event per min) were regarded as having a sEPSC frequency of 0.

RNA sequencing and quantification

Briefly, a custom transcriptome index was created based on cDNA and ncRNA fasta sequences downloaded from Ensembl release 91 (Aken et al., 2016), keeping sequences only from chromosomes 1, 2, …, 22, X and Y. Estimated counts at the transcript level could be obtained using kallisto and summarized to gene level using the tximport R package (Soneson et al., 2015). High mapping rates (~92%) across all iPSC-astrocyte samples were observed and, on average, 13,057 protein coding genes were detected per sample (with at least one transcript per million (TPM)). For accurate comparison, transcript abundances were quantified in the same way as described above for GSE73721 (Zhang et al., 2016), GSE102956 (Lin et al., 2018), GSE97619 (Santos et al., 2017), GSE97904 (Tcw et al., 2017), and fastq files kindly provided by Lischka (Lischka et al., 2018). For comparison across datasets, counts for protein coding genes were summarized and genes with zero counts and genes with zero variance across all samples were removed. Next, counts were transformed using the logarithm Log2 (counts + 1) before applying a quantile normalization using the preprocessCore R library (Bolstad, 2018). Primary fetal and adult astrocyte samples from human tissue (Zhang et al., 2016) were selected to perform gene expression principal component analysis (R prcomp). Then, the gene expression profiles of iPSC-astrocytes were projected onto the principle component space by multiplying the centered data to the principal component loadings.

Differential expression analysis

Comparison of the expression profiles of our iPSC-astrocytes and those published by Lischka (Lischka et al., 2018) was performed by differential expression analysis using DESeq2 (Love et al., 2014). We acknowledge that possible technical confounds could contribute to the gene expression differences observed between datasets of iPSC-astrocytes (library preparation, number of reads detected, mapping rate, etc.). Yet, we considered only those expression differences that were also different when comparing fetal and mature astrocytes from other data (see **Results**) and believe these confounds are unlikely to contribute to both sets of differences. Lowly expressed genes (with less than one TPM across the samples of both datasets) were removed through pre-filtering. DESeq2 default filtering removed gene outliers, after which 14,629 genes remained; this set was considered as the gene background population for further gene functional analyses. A list of genes that were up and down regulated in primary fetal versus primary adult cortical astrocytes (reported in Table S6 by Zhang et al. (2016)) were used for comparison. HGNC symbols were converted into Ensemble gene IDs through biomaRt (Durinck et al., 2009). Genes were considered differentially expressed when the false detection rate (FDR) was < 0.05. Hypergeometric testing was employed to evaluate the significance of the overlap between two lists of genes.

To examine astrocyte reactive genes, we made use of two overlapping signatures of reactive astrocytes, which have been reported in mice (Zamanian et al., 2012), one in response to ischemic insult by occlusion of the middle cerebral artery (MCAO) and the other in response to inflammation upon administration of bacterial lipopolysaccharide (LPS). The top 50 differentially expressed genes found by Zamanian et al. (2012) in each condition and their corresponding controls reported in Table 1 and Table 2 (Zamanian et al., 2012) were included in the comparative analysis.

Gene Ontology analysis

Gene Ontology (GO) annotations were obtained from Ensembl through biomaRt (Durinck et al., 2009). A subset of GO annotations that contained at least 50 genes in our background gene population (large enough to be informative and powerful) was selected and tested for enrichment using a hypergeometric test. The corresponding p-values were adjusted to account for multiple testing (Benjamini-Hotchberg FDR), and a GO term was considered as overrepresented when the FDR < 0.05. For visualization purposes, an intelligible list of overrepresented GO terms was sought and thus REVIGO was used to identify redundant terms (Supek et al., 2011).

Protein-protein interaction analysis

To identify protein-protein interactions between extracellular astrocytic and synaptic genes, a range of sources were integrated: BioGRID (Stark et al., 2006) (retrieved on 09/04/2019), HitPredict (Lopez et al., 2015) (09/04/2019), IntAct (Orchard et al., 2014) (09/04/2019), STRING (Szklarczyk et al., 2019) (09/04/2019), CORUM (Giurgiu et al., 2019) (09/04/2019), Reactome (Fabregat et al., 2018) (09/04/2019), BioPlex2.3 (Huttlin et al., 2017) (09/04/2019), MINT (Licata et al., 2012) (15/09/2017), InBioMap (Li et al., 2017) (15/09/2017). Interactions at the gene level were summarized (Ensembl gene IDs), while removing duplicated interactions

across datasets and self-interacting pairs. Note that only protein links with an experimental score > 0 from the STRING database were considered. Among the set of genes with both higher expression in our iPSC-astrocytes compared to those from Lischka, and higher expression in adult compared to fetal astrocytes from Zhang, the subset of extracellular genes were identified as those annotated to extracellular Gene Ontology terms (GO:0070062, GO:0005576 or GO:0005615). Synaptic genes were identified by using the Obo-Edit software to retrieve all children terms of synapse parts (GO:0044456). Terms were grouped into "pre-synaptic" or "post-synaptic" if these words were found in the term description. Finally, all genes were grouped separately according to their annotation to either the 33 "pre-synaptic" or 47 "post-synaptic" terms.

We tested whether more protein-protein interactions were observed than expected by chance between two sets of genes (i.e. extracellular genes identified in iPSC-astrocytes and genes annotated as either pre- or post-synaptic). First, the number of protein-protein interactions between the two gene sets was counted, then compared to the number of interactions found in each of 10,000 random samples of genes of equal size as the first list (matched for both network node degree and coding sequence length) (Honti et al., 2014). A p-value could then be estimated by counting the number of random samples with an equal or higher number of protein-protein interactions in the real data. Finally, Cytoscape was used for visualization of the protein-protein interaction network (Shannon et al., 2003). To relate these data to different brain cell types, single cell RNA seq expression from diverse brain cell types (Darmanis et al., 2015) were normalized before averaging by cell type (62 astrocytes, 20 endothelial cells, 16 microglia, 131 neurons, 38 oligodendrocytes, and 18 oligodendrocyte precursor cells), and finally the expression was scaled across cell types to create a heatmap.

Supplemental references:

Dallas, M., Boycott, H.E., Atkinson, L., Miller, A., Boyle, J.P., Pearson, H.A., and Peers, C. (2007). Hypoxia suppresses glutamate transport in astrocytes. J. Neurosci. *27*, 3946–3955.

Durinck, S., Spellman, P.T., Birney, E., and Huber, W. (2009). Mapping identifiers for the integration of genomic datasets with the R/ Bioconductor package biomaRt. Nat. Protoc. *4*, 1184–1191.

Fabregat, A., Jupe, S., Matthews, L., Sidiropoulos, K., Gillespie, M., Garapati, P., Haw, R., Jassal, B., Korninger, F., May, B., et al. (2018). The Reactome Pathway Knowledgebase. Nucleic Acids Res. *46*, D649–D655.

Giurgiu, M., Reinhard, J., Brauner, B., Dunger-Kaltenbach, I., Fobo, G., Frishman, G., Montrone, C., and Ruepp, A. (2019). CORUM: the comprehensive resource of mammalian protein complexes-2019. Nucleic Acids Res *47*, D559–D563.

Honti, F., Meader, S., and Webber, C. (2014). Unbiased functional clustering of gene variants with a phenotypic-linkage network. PLoS Comput Biol *10*, e1003815.

Huttlin, E.L., Bruckner, R.J., Paulo, J.A., Cannon, J.R., Ting, L., Baltier, K., Colby, G., Gebreab, F., Gygi, M.P., Parzen, H., et al. (2017). Architecture of the human interactome defines protein communities and disease networks. Nature *545*, 505–509.

Kaech, S., and Banker, G. (2006). Culturing hippocampal neurons. Nat. Protoc. *1*, 2406–2415.

Li, T., Wernersson, R., Hansen, R.B., Horn, H., Mercer, J., Slodkowicz, G., Workman, C.T., Rigina, O., Rapacki, K., Staerfeldt, H.H., et al. (2017). A scored human protein-protein interaction network to catalyze genomic interpretation. Nat Methods *14*, 61–64.

Licata, L., Briganti, L., Peluso, D., Perfetto, L., Iannuccelli, M., Galeota, E., Sacco, F., Palma, A., Nardozza, A.P., Santonico, E., et al. (2012). MINT, the molecular interaction database: 2012 update. Nucleic Acids Res *40*, D857-61.

Lopez, Y., Nakai, K., and Patil, A. (2015). HitPredict version 4: comprehensive reliability scoring of physical protein-protein interactions from more than 100 species. Database (Oxford) *2015*.

Love, M.I., Huber, W., and Anders, S. (2014). Moderated estimation of fold change and dispersion for RNA-seq data with DESeq2. Genome Biol *15*, 550.

Orchard, S., Ammari, M., Aranda, B., Breuza, L., Briganti, L., Broackes-Carter, F., Campbell, N.H., Chavali, G., Chen, C., del-Toro, N., et al. (2014). The MIntAct project--IntAct as a common curation platform for 11

molecular interaction databases. Nucleic Acids Res *42*, D358-63.

Shannon, P., Markiel, A., Ozier, O., Baliga, N.S., Wang, J.T., Ramage, D., Amin, N., Schwikowski, B., and Ideker, T. (2003). Cytoscape: a software environment for integrated models of biomolecular interaction networks. Genome Res *13*, 2498–2504.

Shcheglovitov, A., Shcheglovitova, O., Yazawa, M., Portmann, T., Shu, R., Sebastiano, V., Krawisz, A., Froehlich, W., Bernstein, J.A., Hallmayer, J.F., et al. (2013). SHANK3 and IGF1 restore synaptic deficits in neurons from 22q13 deletion syndrome patients. Nature *503*, 267–271.

Stark, C., Breitkreutz, B.J., Reguly, T., Boucher, L., Breitkreutz, A., and Tyers, M. (2006). BioGRID: a general repository for interaction datasets. Nucleic Acids Res *34*, D535-9.

Supek, F., Bosnjak, M., Skunca, N., and Smuc, T. (2011). REVIGO summarizes and visualizes long lists of gene ontology terms. PLoS One *6*, e21800.

Szklarczyk, D., Gable, A.L., Lyon, D., Junge, A., Wyder, S., Huerta-Cepas, J., Simonovic, M., Doncheva, N.T., Morris, J.H., Bork, P., et al. (2019). STRING v11: protein-protein association networks with increased coverage, supporting functional discovery in genome-wide experimental datasets. Nucleic Acids Res *47*, D607–D613.

Tashiro, A., Zhao, C., Suh, H., and Gage, F.H. (2015). Preparation and Use of Retroviral Vectors for Labeling, Imaging, and Genetically Manipulating Cells. Cold Spring Harb. Protoc. *2015*, 883–888.

Zamanian, J.L., Xu, L., Foo, L.C., Nouri, N., Zhou, L., Giffard, R.G., and Barres, B.A. (2012). Genomic Analysis of Reactive Astrogliosis. J. Neurosci. *32*, 6391–6410.

Zhang, F., Wang, L.P., Brauner, M., Liewald, J.F., Kay, K., Watzke, N., Wood, P.G., Bamberg, E., Nagel, G., Gottschalk, A., et al. (2007). Multimodal fast optical interrogation of neural circuitry. Nature *446*, 633–639.

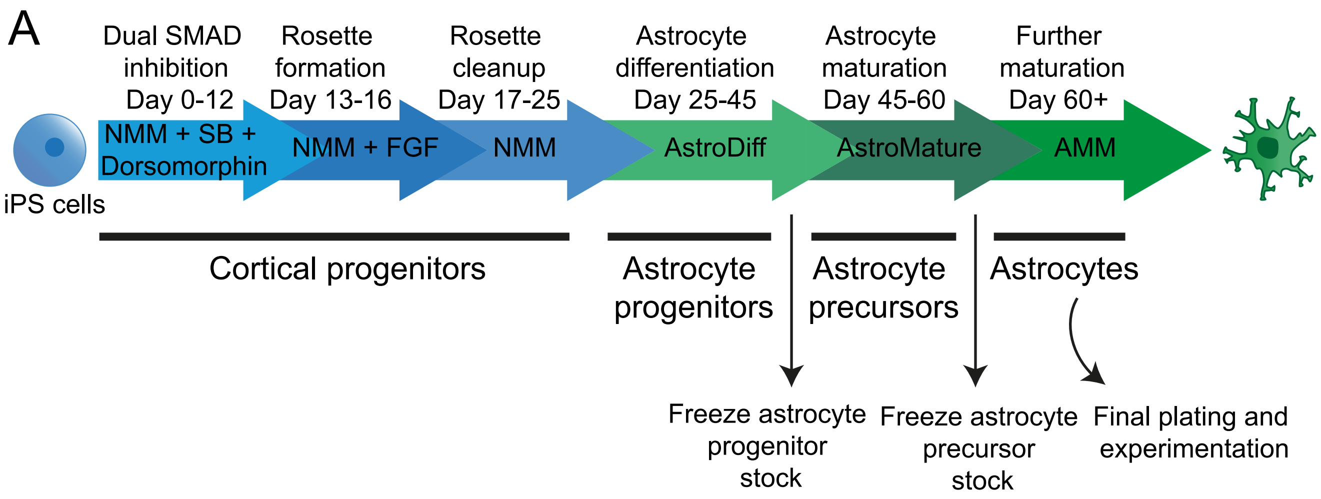

Figure S1. Outline of the astrocyte differentiation protocol. Related to Figure 1. **(A)** Up to day 17-25, the iPSCs follow the protocol for cortical neural induction via dual SMAD inhibition, specified by the (Shi et al., 2012). Around day 25, clean rosettes are switched from a neural maintenance media (NMM) to astrocyte differentiation media (AstroDiff), and subsequently dissociated to single cells once per week. Stocks of proliferative astrocyte progenitors can be frozen and cryostored in liquid nitrogen before switching to astrocyte maturation media around day 45. Weekly dissociations and seeding at lower density yield a population of less proliferative astrocyte precursors, which can also be frozen and cryostored. Further maturation is achieved by switching the media to astrocyte maintenance media (AMM) around day 60, in which astrocytes can be finally plated and maintained for experimentation.

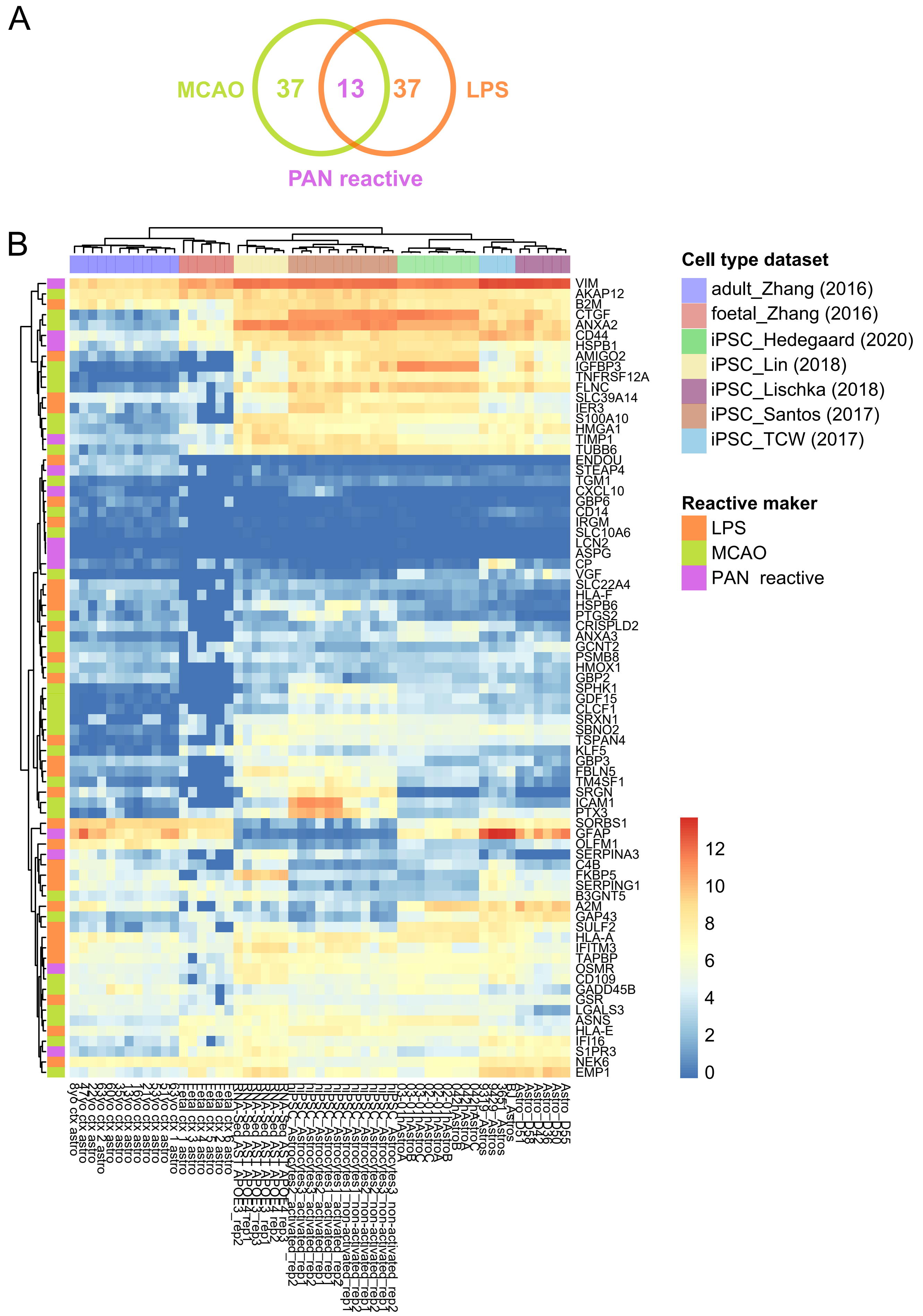

Figure S2. iPSC-astrocytes exhibit similar reactive states across datasets. Related to Figure 1. **(A)** Venn-diagram shows the number of genes associated with an ischemic state after middle cerebral artery occlusion (MCAO), an inflammatory state following stimulation with lipopolysaccharide (LPS), or both (PAN reactive), identified in (Zamanian et al., 2012). **(B)** Comparison of transcriptomic datasets from primary human astrocytes and multiple iPSC-astrocytes (color-coded by cell type and publication). Gene expression is given in log transformed counts per million (log2 (CPM + 1)), scaled by gene.

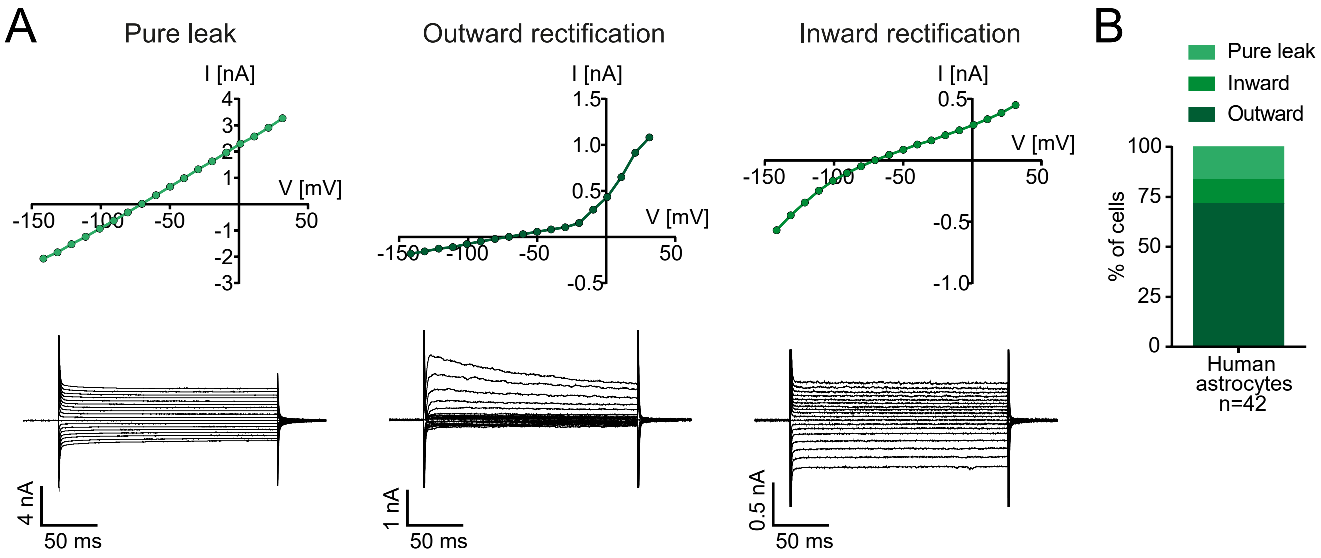

Figure S3. K⁺-current profiles of cortical iPSC-astrocytes. Related to Figure 2. (A) Examples of different K^+ currents exhibited by iPSC-astrocytes. I-V plots (top) and accompanying non-leak subtracted family of current traces elicited by subjecting an astrocyte to a series of voltage steps (bottom). Astrocytes, aged 97 ± 3.5 days showed either linear 'pure leak' (left), outward rectifying (middle), or inward rectifying (right) K^+ currents. **(B)** The majority of astrocytes showed outward rectifying K^+ currents (71%), but a subset displayed purely leak K^+ currents (17%) or inward rectifying K^+ currents (12%). Of the five astrocytes displaying inward rectification, two were also tested for glutamate uptake currents and both exhibited this property.

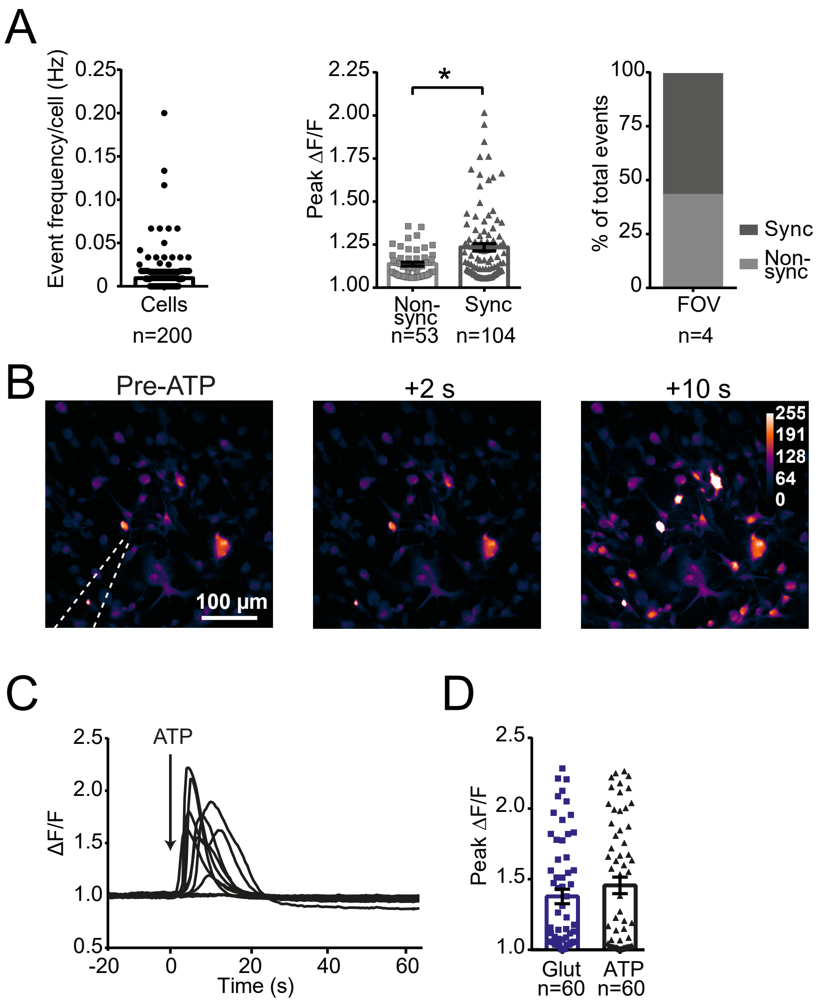

Figure S4. Spontaneous and evoked Ca^{2+} responses in iPSC-astrocytes. Related to Figure 3. **(A)** Quantifications of spontaneous Ca^{2+} events from the same 4 FOV's as shown in Figure 3, representing 4 cultures across 3 cell lines aged 101 ± 3.9 days. 50 random ROIs were drawn on each FOV, so n=200 cells. The frequency of Ca^{2+} events per cell (left), the peak ΔF/F amplitude of non-synchronous versus synchronous events (middle) and the proportion of event types (43% non-synchronous, 57% synchronous, right). **(B)** Ca^{2+} events elicited by a 500 ms puff of 1 mM ATP, delivered via a patch-pipette. **(C)** ΔF/F traces from 10 representative ROIs from the FOV shown in 'B'. **(D)** Comparison of Ca^{2+} event amplitudes between events evoked by glutamate and ATP in a 96 days old culture (2 FOVs per transmitter, each with 30 ROIs, so n=60). FOV: fields of view. * $p < 0.05$

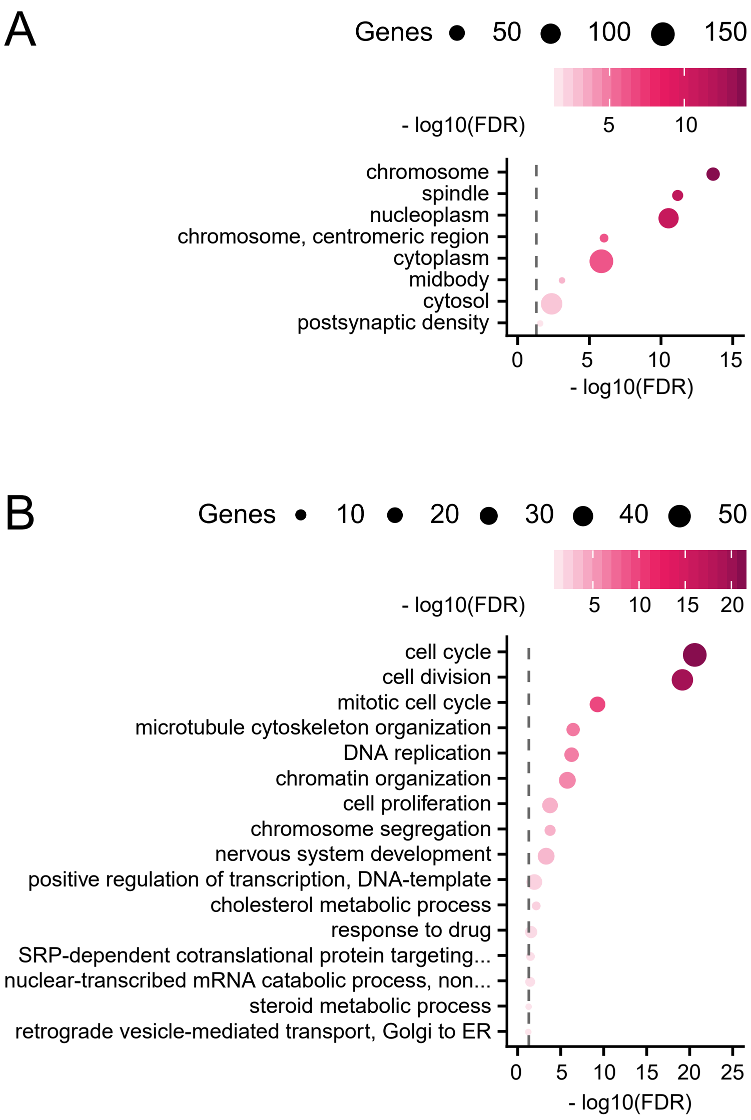

Figure S5. Relative astrocyte immaturity is associated with nuclear components and proliferative processes in iPSC-astrocytes. Related to Figure 6. **(A)** Overrepresented cellular components and **(B)** biological processes among the 279 genes with higher expression in both primary fetal astrocytes (compared to adult) and the 'iPSC Lischka' astrocytes, compared to 'iPSC Hedegaard'. Size of the circles indicate the number of genes annotated to each GO terms while colours reflect the log10 transformed FDR. GO: gene ontology, FDR: false discovery rate.

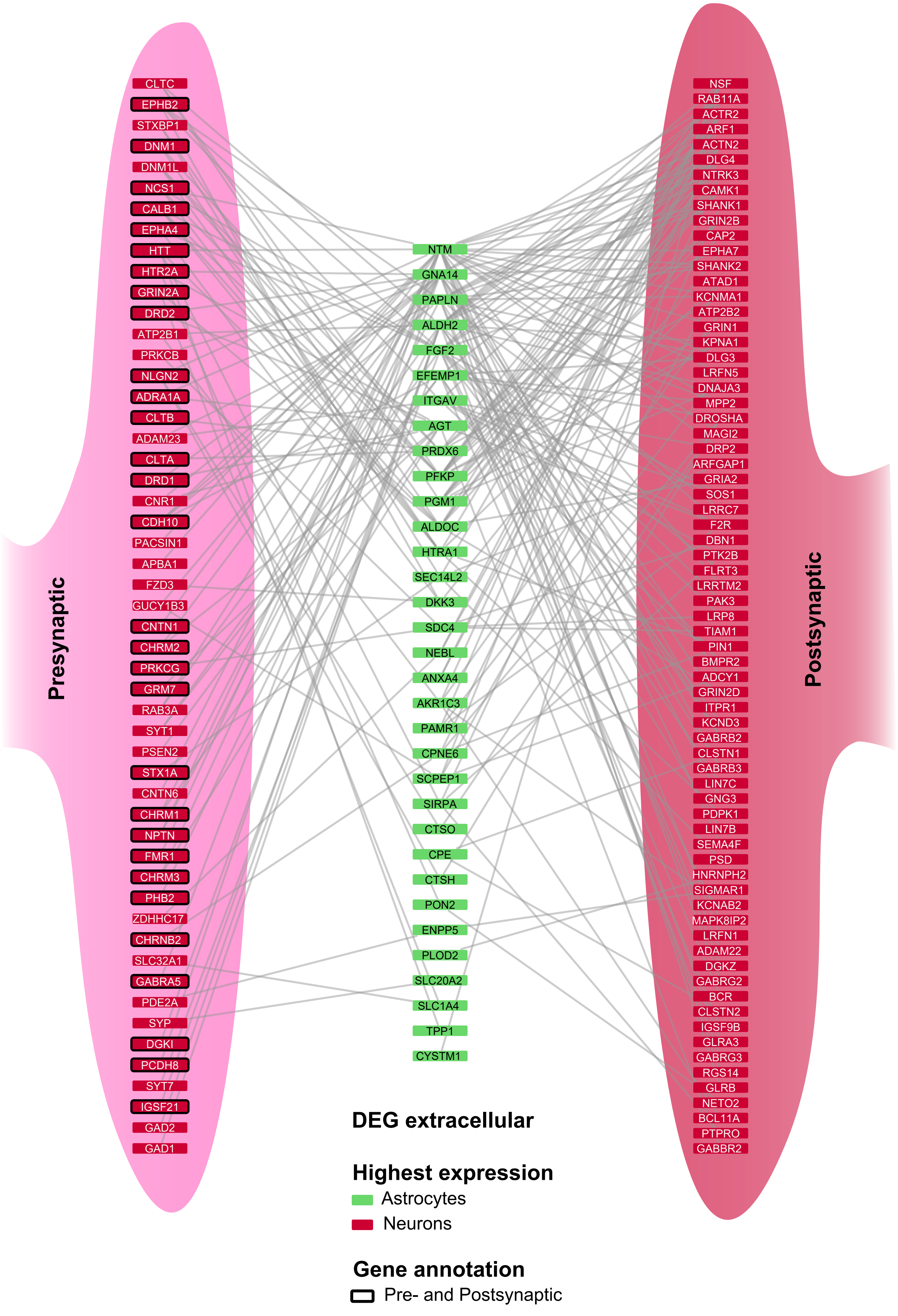

Figure S6. Expanded protein-protein interaction network. Related to Figure 7. Enlarged view of a subset of the protein-protein interaction network shown in Figure 7B. Protein interactions are highlighted between extracellular genes that are most highly expressed in astrocytes (green), and synaptic genes that are most highly expressed in neurons (red). The astrocyte extracellular genes are ranked from top to bottom based on the number of interactions that their proteins show with pre-synaptic (left) and post-synaptic (right) proteins. A black border highlights genes annotated to both pre-and post-synaptic sets.
