# Supplementary material for: Pro-maturational Effects of Human iPSC-Derived Cortical Astrocytes upon iPSC-Derived Cortical Neurons
Source: Stem Cell Reports. 2020 Jun 4;15(1):38–51. doi: 10.1016/j.stemcr.2020.05.003 (PMC7363746; doi:10.1016/j.stemcr.2020.05.003)
Supplement: Document S2. Article plus Supplemental Information [file mmc2.pdf]

Stem Cell Reports
Article

OPEN ACCESS

Pro-maturational Effects of Human iPSC-Derived Cortical Astrocytes upon iPSC-Derived Cortical Neurons

Anne Hedegaard,[1] Jimena Monzón-Sandoval,[2] Sarah E. Newey,[1] Emma S. Whiteley,[1] Caleb Webber,[2,3] and Colin J. Akerman[1,*]

[1]Department of Pharmacology, University of Oxford, Mansfield Road, Oxford OX1 3QT, UK
[2]UK Dementia Research Institute, Cardiff University, Maindy Road, Cardiff CF24 4HQ, UK
[3]Department of Physiology, Anatomy and Genetics, University of Oxford, South Parks Road, Oxford OX1 3QX, UK
*Correspondence: colin.akerman@pharm.ox.ac.uk
https://doi.org/10.1016/j.stemcr.2020.05.003

SUMMARY

Astrocytes influence neuronal maturation and function by providing trophic support, regulating the extracellular environment, and modulating signaling at synapses. The emergence of induced pluripotent stem cell (iPSC) technology offers a human system with which to validate and re-evaluate insights from animal studies. Here, we set out to examine interactions between human astrocytes and neurons derived from a common cortical progenitor pool, thereby recapitulating aspects of *in vivo* cortical development. We show that the cortical iPSC-derived astrocytes exhibit many of the molecular and functional hallmarks of astrocytes. Furthermore, optogenetic and electrophysiological co-culture experiments reveal that the iPSC-astrocytes can actively modulate ongoing synaptic transmission and exert pro-maturational effects upon developing networks of iPSC-derived cortical neurons. Finally, transcriptomic analyses implicate synapse-associated extracellular signaling in the astrocytes' pro-maturational effects upon the iPSC-derived neurons. This work helps lay the foundation for future investigations into astrocyte-to-neuron interactions in human health and disease.

INTRODUCTION

Most of our knowledge regarding astrocyte-neuron interactions has been gained from animal models, which have been particularly important in advancing understanding at a molecular and cellular level. From such studies, we know that during development and in the mature brain, astrocytes provide important trophic and homeostatic support to neurons (Verkhratsky and Nedergaard, 2018). Astrocyte-to-neuron signaling is thought to promote many aspects of neuronal development, including the emergence of electrical excitability and the formation and maturation of synapses (Allen and Eroglu, 2017). Through their close association with synapses, astrocytes also support ongoing neurotransmission by regulating concentration changes in extracellular ions and neurotransmitters (Perea and Araque, 2010; Verkhratsky and Nedergaard, 2014) and function as a glio-neuronal processing unit that can monitor and modulate ongoing synaptic transmission on rapid timescales (Fellin and Carmignoto, 2004; Panatier et al., 2011).

The development of induced pluripotent stem cell (iPSC) technology offers the potential to test established knowledge gained from rodent models and, importantly, provides opportunities to test hypotheses in a human disease context. By adapting culture conditions, iPSCs can give rise to specific cell types in a process that replicates key aspects of *in vivo* development. To generate human astrocytes, iPSC differentiation protocols have generally targeted the gliogenic JAK-STAT pathway via manipulations of culture media. This strategy enables re-capitulation of stages of *in vivo* development leading to astrocyte production over different timescales (Krencik et al., 2011; Serio et al., 2013; Shaltouki et al., 2013; Sloan et al., 2017), and with the potential for regional patterning during the preceding neurogenic stage (Liu and Zhang, 2011; Roybon et al., 2013). Another strategy has been to directly convert fibroblasts into astrocytes via overexpression of transcription factors associated with the JAK-STAT pathway (Canals et al., 2018; Tchieu et al., 2019). Such approaches have led to recent co-culture studies in which iPSC-derived astrocytes are reported to exhibit pro-maturational effects upon co-cultured neurons, such as enhancing the intrinsic excitability of the neurons (Kayama et al., 2018; Klapper et al., 2019; VanderWall et al., 2019). Meanwhile, co-culturing with iPSC-derived astrocytes has had mixed results in terms of influencing synaptic transmission and synaptic maturation. Some reports have shown that iPSC-derived astrocytes can enhance the synaptic signaling between iPSC-derived retinal ganglion cells (VanderWall et al., 2019) and so-called induced neurons (Canals et al., 2018; Tchieu et al., 2019), whereas others have not observed such effects, perhaps due to the level of astrocyte maturity (Lischka et al., 2018). Meanwhile, to our knowledge, rapid astrocyte-mediated modulation of ongoing synaptic transmission has not previously been demonstrated in an iPSC-derived human co-culture.

During *in vivo* cortical development, neurons and astrocytes are thought to derive from a common progenitor pool (Rowitch and Kriegstein, 2010). Radial glial cells, the

This is an open access article under the CC BY license (http://creativecommons.org/licenses/by/4.0/).

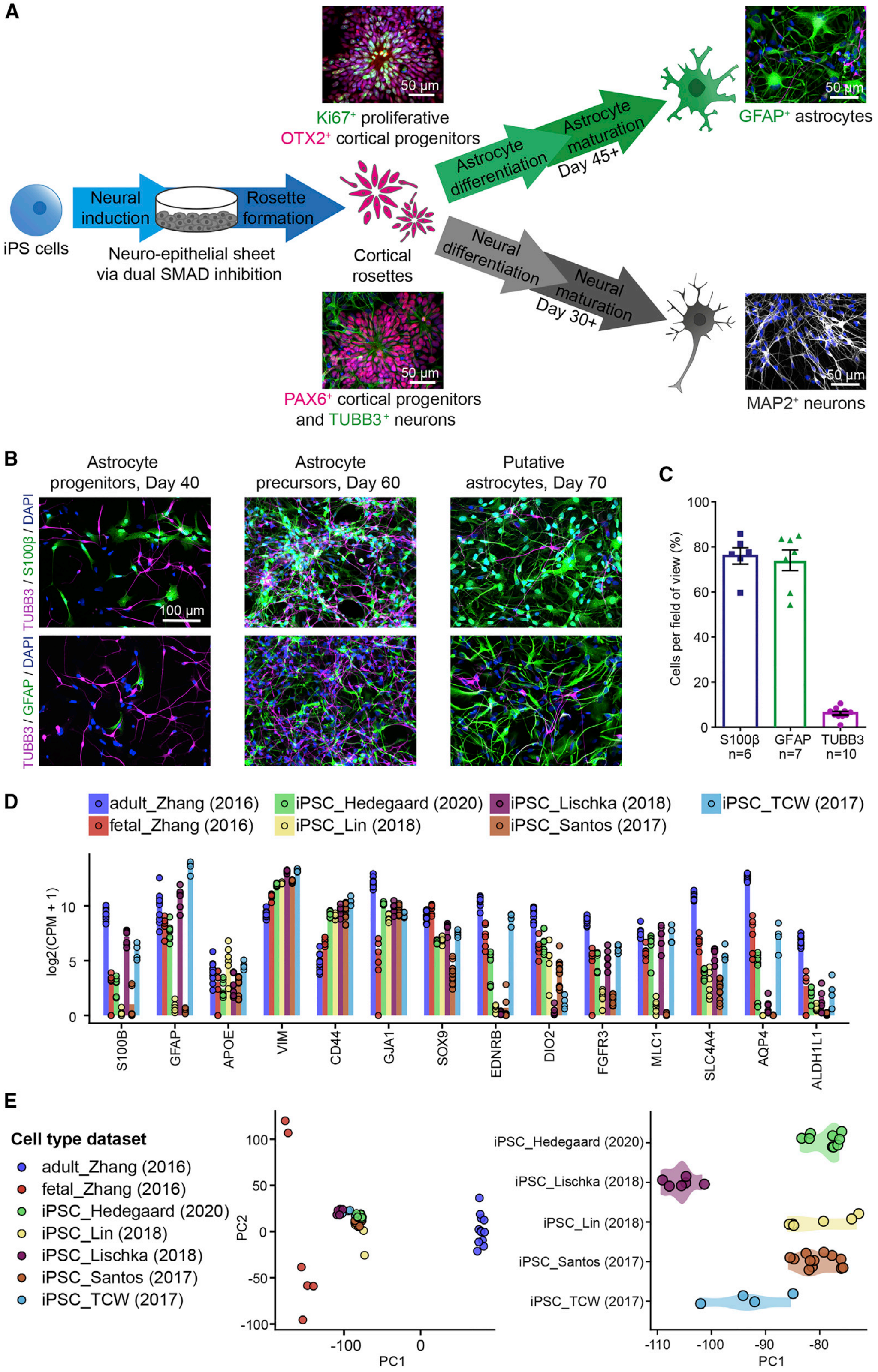

(legend on next page)

principal progenitor cell type in embryonic cortex, initially undergo asymmetrical divisions to produce neurons or neurogenic intermediate progenitor cells. As the period of neurogenesis finishes, there is a switch to gliogenesis, and radial glial cells can give rise to astrocytes (Rowitch and Kriegstein, 2010). Here, we set out to study the interactions between human astrocytes and neurons independently generated from a common cortical progenitor pool. The molecular identity and functional properties of the astrocytes are determined by immunocytochemistry, transcriptomics, and targeted recordings. Co-culture studies then demonstrate that the cortically derived astrocytes exhibit key interactions with iPSC-derived cortical neurons, including the ability to rapidly modulate ongoing synaptic signaling and to exert pro-maturational effects on synaptic networks. Consistent with this, transcriptomic analyses identify astrocytic extracellular signaling at neuronal pre- and post-synaptic sites.

RESULTS

Deriving Human Astrocytes and Neurons from a Common Cortical Progenitor Pool

Since cortical neurons and astrocytes can originate from the same progenitors *in vivo* (Rowitch and Kriegstein, 2010), we set out to generate iPSC-derived neurons and astrocytes from a common cortical progenitor pool. The protocol involves initiation of the cortical differentiation pathway via dual SMAD inhibition (Chambers et al., 2009), as part of a well-established cortical neuron induction protocol (Shi et al., 2012). This approach produced self-organizing rosette structures composed of PAX6^{+} radial glia progenitors, the cortical identity of which was verified by widespread OTX2 expression. These radial glia progenitors were subsequently directed down one of two differentiation pathways to generate either MAP2^{+} neurons or cells of an astrocytic fate (Figure 1A, further details in Figure S1). We refer to these as cortical iPSC-neurons and cortical iPSC-astrocytes, respectively.

We first tracked the commitment to astrocytic fate by immunofluorescence staining (described in Supplemental Experimental Procedures) for the classic astrocyte markers, S100β and GFAP (Ludwin et al., 1976). At the putative astrocyte progenitor stage (<40 days), S100β and GFAP were sparsely expressed (Figure 1B). By ~60 days, staining was consistent with an astrocyte precursor stage at which S100β is more abundant than GFAP (Barnabé-Heider et al., 2005). After a further 10–20 days, all cell lines were enriched for putative astrocytes. Quantification showed that as a proportion of all DAPI^{+} cells, 76% were S100β^{+} and 74% were GFAP^{+}, while only 6% were expressing the neuronal marker TUBB3^{+} (Figure 1C).

We next used RNA sequencing (RNA-seq) analysis to gain greater insight into the expression profile of our iPSC-astrocytes. We compared our data with other published transcriptomic datasets on human iPSC-derived astrocytes, as well as RNA-seq profiles of primary human fetal and adult cortical astrocytes (Zhang et al., 2016; Figure 1D). Our iPSC-astrocytes expressed a set of well-known astrocyte-specific genes (Zhang et al., 2016) at either similar levels (e.g., *APOE, VIM*, *CD44*, and *GJA1*) or higher levels (e.g., *AQP4*, *EDNRB*, and *DIO2*), compared with other iPSC-astrocyte differentiation protocols (Figure 1D). All iPSC-astrocyte populations were also similar in terms of the expression of reactive markers, consistent with the idea that *in vitro* cell culture tends to lead to a more reactive astrocytic state (Cahoy et al., 2008; Liddelow and Barres, 2017) (Figure S2). To assess the relative maturity of our iPSC-astrocytes, we projected the RNA-seq profiles of iPSC-astrocytes onto the principal component (PC) space created from the gene expression profiles of the primary fetal and adult astrocytes (Figure 1E). iPSC-derived astrocytes generally clustered together and were closer to primary fetal astrocytes than to adult astrocytes, although modest differences along PC1 were apparent between the

Figure 1. Human Cortically Derived iPSC-Astrocytes Express Canonical Markers and Are Comparable with Other iPSC-Astrocytes
(A) Schematic of the differentiation from iPSCs toward a common pool of PAX6^{+} cortical progenitor cells, which can then be driven toward generating either GFAP^{+} astrocytes or MAP2^{+} cortical neurons. See also Figure S1.
(B) Examples of increasing expression of astrocytic markers, S100β (top) and GFAP (bottom), tracked throughout the astrocyte differentiation and maturation process.
(C) Proportion of S100β^{+}, GFAP^{+}, and TUBB3^{+} cells quantified from four cultures across three cell lines, at the mature stage of the astrocyte protocol (sample sizes represent fields of view per marker, 70 ±5.6 days at imaging).
(D) Gene expression levels for astrocyte-specific markers in iPSC-astrocytes generated from the present study (iPSC_Hedegaard; n = 9 cultures, comprising three cultures from each of three cell lines, 96 ± 3.3 days). For comparison, transcript abundances of fetal and adult human cortical astrocytes (Zhang et al., 2016) and previously published profiles of iPSC-derived astrocytes (Lin et al., 2018; Lischka et al., 2018; Santos et al., 2017; Tcw et al., 2017) are included. Gene expression levels are logarithm scaled counts per million (log(CPM +1)). See also Figure S2.
(E) Principal component analysis separated fetal and adult astrocytes from Zhang et al. (2016) along the first component. Gene expression profiles from published iPSC-astrocytes datasets were projected onto the space created by the first two principal components (left). PC1 discriminates iPSC-astrocytes by dataset (right).

different iPSC-astrocyte populations (Figure 1E). These molecular marker and gene expression data suggest that astrocytes derived from a cortical progenitor population are comparable with other iPSC-derived astrocytes.

Functional Characterization of Cortically Derived Human iPSC-Astrocytes

In contrast to neurons, astrocytes are regarded as non-excitable under physiological conditions. In keeping with this, none of our iPSC-astrocytes could generate action potentials (Figures 2A and 2B), and while all neurons displayed large voltage-gated Na^+ ($VGNa^+$) currents, the iPSC-astrocytes lacked Na^+ currents of comparable size (astrocytes, -0.18 ± 0.03 nA; neurons, -1.7 ± 0.2 nA; $p < 0.0001$, unpaired Mann-Whitney test; Figures 2B and 2C). The iPSC-astrocytes also exhibited lower membrane resistances, consistent with more permeable membranes (astrocytes, 0.39 ± 0.04 GΩ; neurons, 1.2 ± 0.1 GΩ; $p < 0.0001$, unpaired Mann-Whitney test; Figure 2C). *In vivo*, syncytia of gap-junction-coupled astrocytes are considered vital for regulating the levels of substances in the extracellular space, with a primary function to clear and disperse excess glutamate and K^+ (Theis et al., 2005; Verkhratsky and Nedergaard, 2018). Firstly, the presence of gap junctions was revealed by patching and filling individual iPSC-astrocytes with biocytin (Figure 2D), which spread to an average of 61 ± 18 cells (Figure 2E) and overlapped with GFAP staining. Secondly, the activity of astrocytic glutamate transporters was detected both as a net inward current in individual astrocytes following glutamate application (37% of recorded cells; Figures 2F and 2G) and at a population level using an enzymatic glutamate uptake assay (astrocytic uptake, 19.8 ± 5.6 μM/mg protein/min; $p = 0.031$, one-sample Wilcoxon signed-rank test; Figure 2H; see details on both glutamate uptake assays in Supplemental Experimental Procedures). Thirdly, a subset of the iPSC-astrocytes displayed inward rectifying K^+ currents (Figure S3), consistent with a contribution of $K_{ir}4.1$ channels, which are implicated in K^+ and glutamate buffering (Djukic et al., 2007). Together these results demonstrate that the cortically derived iPSC-astrocytes exhibit non-excitable biophysical properties, gap-junction coupling, plus the capacity for K^+ and glutamate buffering, which are key functional properties associated with astrocytes.

Cortically Derived iPSC-Astrocytes Respond to Neurotransmitters and Engage in Rapid Astrocyte-to-Neuron Signaling

Astrocytes are fine-tuned to sense local neuronal activity and respond by exhibiting both spontaneous and neurotransmitter-evoked intracellular Ca^{2+} increases (Aguado et al., 2002; Porter and McCarthy, 1996). To investigate Ca^{2+} signaling in the cortically derived iPSC-astrocytes, cultures were loaded with a membrane-permeable Ca^{2+} dye (OGB-1; Figure 3A; see details on Ca^{2+} imaging in Supplemental Experimental Procedures). In the absence of external stimuli, spontaneous Ca^{2+} events occurred in all astrocyte cultures, with an average of 41.5% of cells exhibiting events. These Ca^{2+} events could be classified as occurring either in an individual astrocyte (non-synchronous events, with a frequency of 0.13 ± 0.01 Hz) or simultaneously across multiple astrocytes (synchronous events, 0.34 ± 0.2 Hz; Figures 3A and S4). Furthermore, astrocytic Ca^{2+} events could be evoked by local application of glutamate (Figure 3B) or ATP (Figure S4) delivered via a patch pipette. Both neurotransmitters elicited robust and rapid Ca^{2+} elevations in the astrocytes.

Such neurotransmitter-evoked Ca^{2+} responses are often viewed as part of a reciprocal communication system between astrocytes and neurons, commonly referred to as gliotransmission (Di Castro et al., 2011; Navarrete et al., 2013; Panatier et al., 2011). We therefore investigated the potential of our cortically derived iPSC-astrocytes to signal in the opposite direction, from astrocyte to neuron. We performed co-culture experiments using iPSC-derived cortical neurons and adopted an optogenetic strategy used in rodent cortex, where astrocytic Ca^{2+} events have been shown to initiate glutamatergic gliotransmission that enhances synaptic transmission between nearby neurons (Perea et al., 2014; Sasaki et al., 2012). To achieve this, we recorded spontaneous excitatory post-synaptic currents (sEPSCs) from the iPSC-derived neurons, while selectively stimulating nearby iPSC-astrocytes expressing the depolarizing and Ca^{2+}-permeable opsin, Channelrhodopsin-2 (ChR2; Nagel et al., 2003) (Figures 3C and 3D). Upon stimulation of the $ChR2^+$ astrocytes with blue light pulses, a significant increase in the frequency of sEPSCs was observed in the neurons (1.9 ± 0.3-fold increase compared with baseline; $p = 0.02$, Wilcoxon signed-rank test; Figures 3D and 3E). Furthermore, this astrocyte-mediated enhancement in sEPSC frequency was dependent on metabotropic glutamate receptor (mGluR) activation, as has also been reported for rodent cortical astrocytes (Perea et al., 2014). In a subset of neurons confirmed to exhibit astrocyte-enhanced sEPSCs, the general mGluR blocker, α-methyl-4-carboxyphenylglycine (MCPG), abolished the effects of astrocyte stimulation ($p = 0.5$, Wilcoxon signed-rank test; Figures 3E and 3F). Taken together, these experiments demonstrate that the cortically derived iPSC-astrocytes are capable of engaging in bidirectional signaling with neurons, as they can both respond to neurotransmitters and influence ongoing synaptic transmission.

Co-culture with Cortically Derived iPSC-Astrocytes Enhances Neuronal and Synaptic Network Maturation

In addition to regulating ongoing synaptic transmission, astrocytes also influence neuronal and synaptic maturity

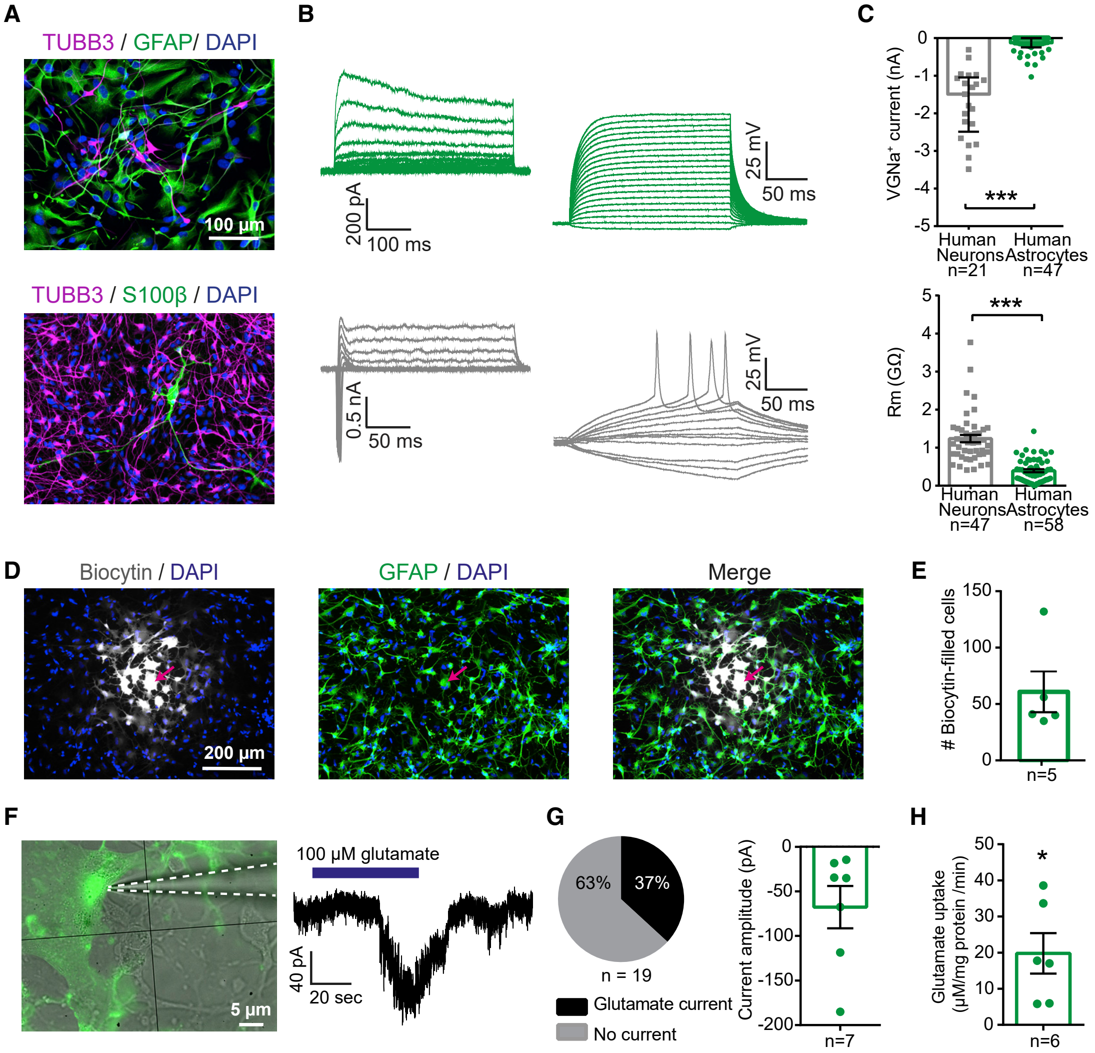

Figure 2. Functional Characteristics of Cortically Derived iPSC-Astrocytes

(A) Example GFAP$^+$ astrocyte culture (55 days; top) and TUBB3$^+$ neuronal culture (100 days; bottom). Cultures were highly enriched for their respective cell type, but low numbers of TUBB3$^+$ cells were observed in astrocyte cultures, and low numbers of S100β$^+$ astrocytes were observed in long-term neuronal cultures.

(B) Example leak-subtracted current responses (left) and voltage responses (right) from an iPSC-astrocyte (99 days; top) and an iPSC-neuron (91 days; bottom).

(C) Comparison of peak voltage-gated sodium (VGNa$^+$) currents (top) and membrane resistance (Rm; bottom) between astrocytes and neurons. Sample sizes represent individual cells recorded from 14 cultures across four cell lines (astrocytes 97 ± 3.5 days) and 13 cultures across three cell lines (neurons 92 ± 3.3 days).

(D) Example gap-junction connected iPSC-astrocytes revealed by filling a single astrocyte with biocytin (arrow; 103 days).

(E) Number of gap-junction connected cells quantified from immunolabeling with streptavidin (n = 5 patched astrocytes from a 103-day-old culture).

(F) Example whole-cell patch-clamp recording from an iPSC-astrocyte (123 days; left) showing an inward current in response to bath application of glutamate (right), consistent with uptake (Dallas et al., 2007).

(G) Proportion of iPSC-astrocytes that showed a significant glutamate-uptake current (left; n = 19 astrocytes recorded from eight cultures across four cell lines, 95 ± 5.3 days; see Supplemental Experimental Procedures). Current amplitudes are shown for the astrocytes that exhibited glutamate uptake (right).

(H) Glutamate uptake capacity of iPSC-astrocytes assessed by an enzymatic absorbance assay (n = 6 cultures comprising two cultures from each of three cell lines, 73 ± 6.7 days; see Supplemental Experimental Procedures). $^*p < 0.05$, $^{***}p < 0.001$.

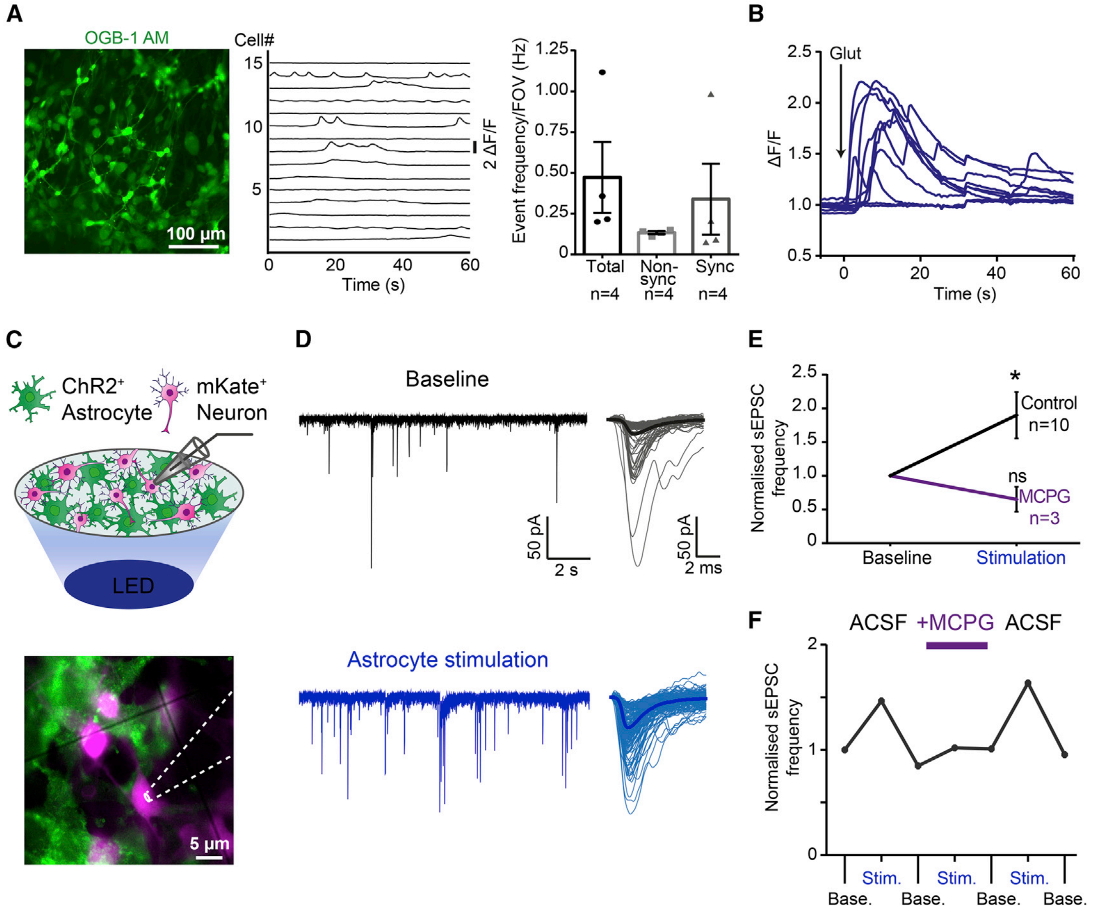

Figure 3. Cortically Derived iPSC-Astrocytes Engage in Ca^{2+} Signaling and Astrocyte-to-Neuron Gliotransmission

(A) Human iPSC-astrocyte culture loaded with OGB-1 AM dye (left; 96 days) and ΔF/F traces from 15 astrocytes in the same culture (middle). Population data on spontaneous Ca^{2+} event frequency (right) for either all Ca^{2+} events in a recording (Total), Ca^{2+} events occurring in individual astrocytes (Non-sync), or Ca^{2+} events occurring simultaneously across multiple astrocytes (Sync) (n = 4 fields of view [FOV] from four cultures across three cell lines, 101 ± 3.9 days).

(B) Time-locked Ca^{2+} events were elicited by focal delivery of 100 μM glutamate via a patch pipette (n = 10 astrocytes from a 96-day-old culture).

(C) Optogenetic experimental design for assessing astrocyte-to-neuron gliotransmission (top). $mKate^+$ iPSC-neurons (magenta) were targeted for recordings (bottom), and co-cultured $ChR2^+$ iPSC-astrocytes (green) were optically stimulated via a blue LED.

(D) Example spontaneous excitatory synaptic currents (sEPSCs) recorded from an iPSC-neuron (neurons 60 days at recording, astrocytes 89 days, co-cultured for 20 days) under baseline conditions (top) and during astrocyte stimulation (450 nm LED; 250 ms pulse duration at 2–4 Hz; bottom). An individual trace (left) and overlays of detected sEPSCs (right) are shown, with the average sEPSC in bold.

(E) Optical stimulation of astrocytes generates a significant increase in sEPSC frequency under control conditions, which is blocked by the mGluR antagonist, MCPG (0.8 mM). All values normalized to baseline frequency. Sample sizes represent individual neurons recorded from two cultures, one cell line (neurons 58 ± 2 days at recording, astrocytes 103 ± 0.5 days, co-cultured for 21 ± 2.5 days).

(F) Individual experiment in which astrocyte stimulation increased neuronal sEPSCs before application of MCPG, and after washout of MCPG, but not in the presence of MCPG. $^*p < 0.05$.

during development. When co-cultured with immature iPSC-neurons, human iPSC-astrocytes have been shown to enhance neuronal intrinsic excitability (Kayama et al., 2018; Klapper et al., 2019; VanderWall et al., 2019) and increase the number of synaptic puncta (Canals et al., 2018; Klapper et al., 2019; Krencik et al., 2011; Lischka et al., 2018; Serio et al., 2013; Shaltouki et al., 2013; Sloan et al., 2017; Tchieu et al., 2019; VanderWall et al., 2019). However, a limited number of studies have demonstrated functional maturation of synaptic networks (Canals et al., 2018; Tchieu et al., 2019; VanderWall et al., 2019), with one study reporting no maturational effects upon neuronal excitability or synaptic networks (Lischka et al., 2018). To examine whether our iPSC-astrocytes promote neuronal excitability

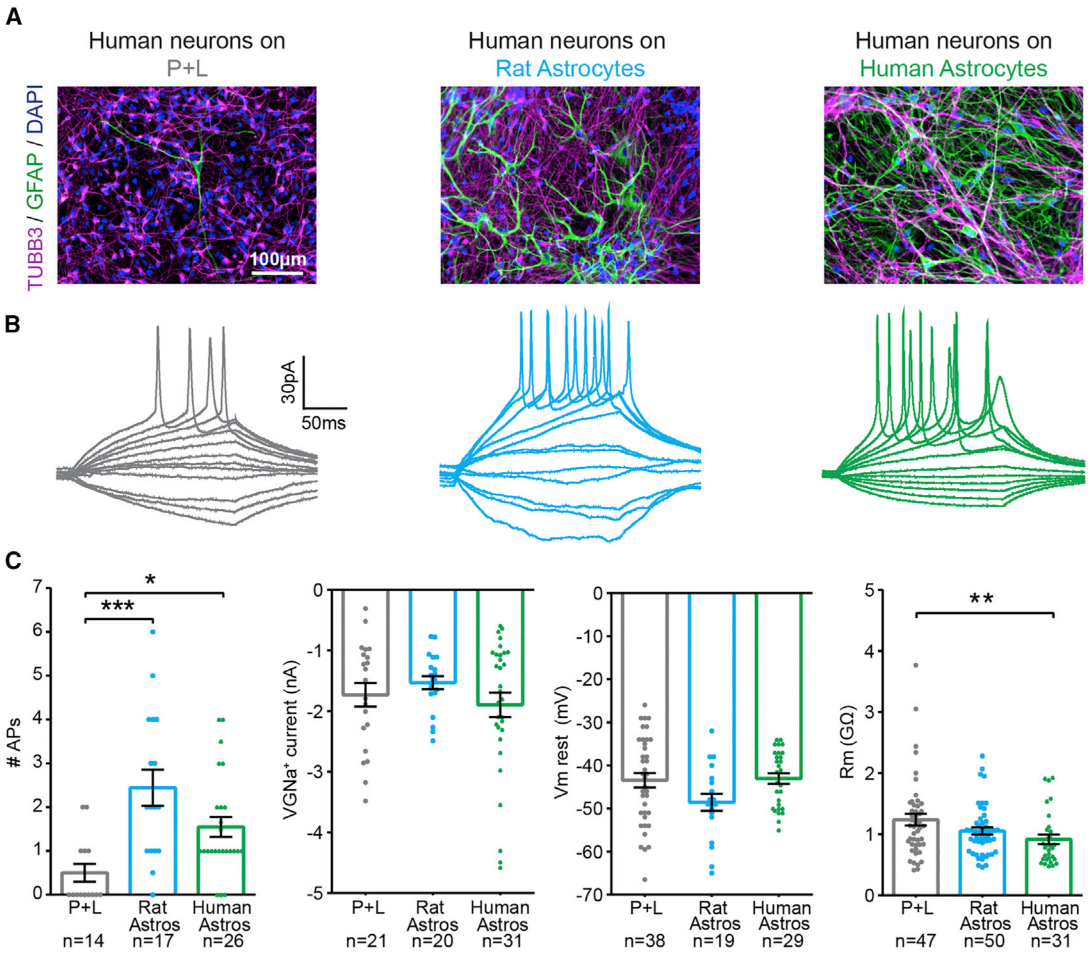

Figure 4. Co-culture with iPSC-Astrocytes Enhances Cortical iPSC-Neuron Excitability

(A) Example immunofluorescence images of human iPSC-neurons grown in three different culture conditions: on polyornithine and laminin-coated coverslips (P + L, neurons 127 days; left), as co-cultures with rat astrocytes (neurons 79 days, co-cultured for 36 days; middle), or as co-cultures with human iPSC-astrocytes (neurons 88 days, astrocytes 117 days, co-cultured for 47 days; right).

(B) Example action potentials evoked in response to square current steps across the three culture conditions.

(C) Population data on the number of action potentials, amplitude of peak $VGNa^+$ currents, resting membrane potential (Vm rest), and membrane resistance (Rm) across the three culture conditions. Sample sizes represent individual neurons recorded from 13 cultures across three cell lines (P + L neurons: 92 ± 3.3 days), 12 cultures across two cell lines (on rat astrocytes: neurons 93 ± 4.9 days, co-cultured for 51 ± 2.9 days), and five cultures across two cell lines (on human astrocytes: neurons 84 ± 7.6 days, astrocytes 120 ± 9.5 days, co-cultured for 44 ± 7.7 days). *$p < 0.05$, **$p < 0.01$, ***$p < 0.001$.

and synaptic maturation, $mKate^+$ iPSC-neurons were grown under one of the following three conditions: (1) on polyornithine and laminin (P + L), (2) as co-cultures on rat cortical astrocytes, or (3) as co-cultures on human iPSC-derived cortical astrocytes of the same donor origin (Figure 4A). Whole-cell patch-clamp recordings revealed that co-culture with either rat astrocytes or human iPSC-astrocytes significantly enhanced the propensity of the neurons to fire action potentials (0.5 ± 0.2 action potentials on P + L, 2.4 ± 0.4 action potentials on rat astrocytes; $p = 0.0002$, and 1.5 ± 0.2 action potentials on human astrocytes; $p = 0.013$, unpaired Kruskal-Wallis with Dunn's multiple comparisons test; Figures 4B and 4C). This increase in neuronal excitability was not associated with differences in the amplitude of $VGNa^+$ currents ($p = 0.877$, unpaired Kruskall-Wallis test, Figure 4C), or resting membrane potentials ($p = 0.121$, unpaired Kruskall-Wallis test), between the three conditions. However, a decrease in membrane resistance was observed between the control (P + L) and the human iPSC-astrocyte co-culture condition (1.2 ± 0.1 GΩ on P + L, 1.1 ± 0.06 GΩ on rat astrocytes, and 0.92 ± 0.08 GΩ on human iPSC-astrocytes; $p = 0.008$, unpaired Kruskall-Wallis with Dunn's multiple comparisons test; Figure 4C), consistent with enhanced iPSC-neuron maturity (Bardy et al., 2016).

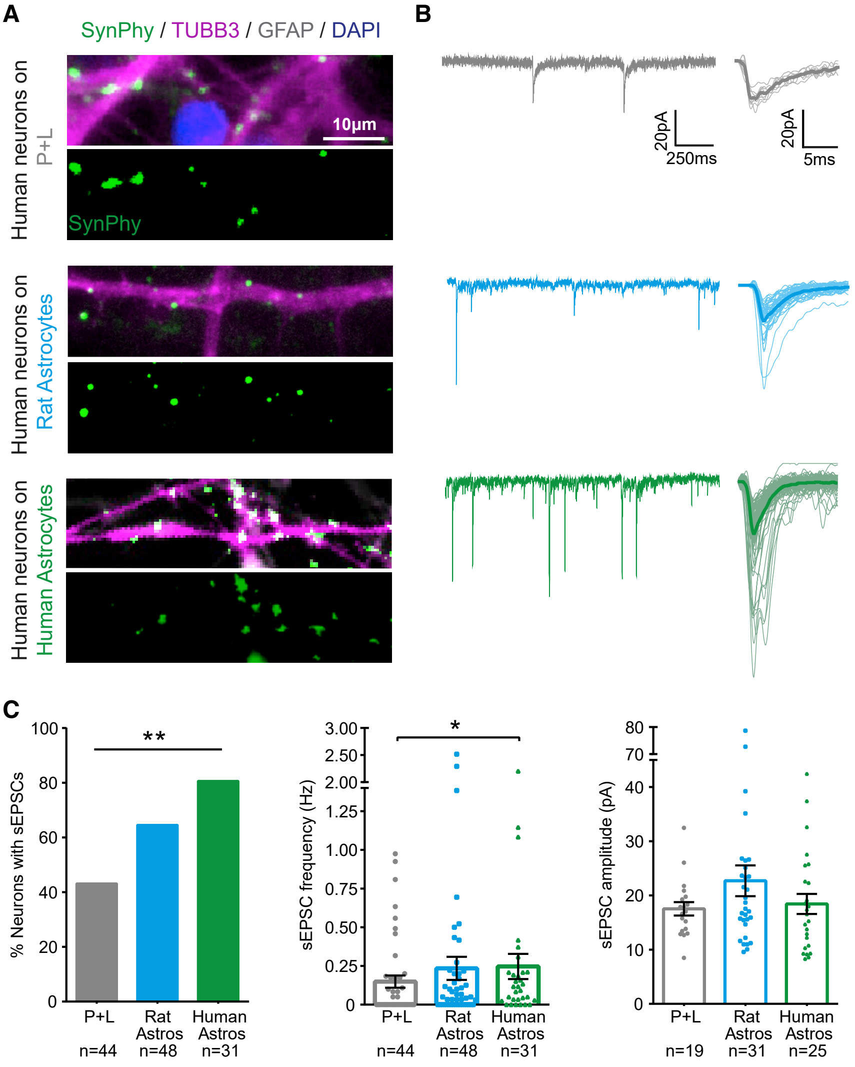

Figure 5. Co-culture with iPSC-Astrocytes Enhances Synaptic Networks among Cortical iPSC-Neurons

(A) Example immunofluorescence images showing putative synaptophysin-positive ($SynPhy^{+}$) pre-synaptic connections overlapping with $TUBB3^{+}$ neuronal processes in the three culture conditions: on poly-ornithine and laminin-coated coverslips (P + L, neurons 73 days; top), as co-cultures with rat astrocytes (neurons 62 days, co-cultured for 22 days; middle), or as co-cultures with human iPSC-astrocytes (neurons 80 days, astrocytes 104 days, co-cultured for 37 days; bottom).

(B) Example whole-cell patch-clamp recordings of sEPSCs across the three culture conditions. An individual trace is shown (left) and overlays of detected sEPSCs (right), with the average sEPSC in bold.

(C) Population data showing the proportion of neurons receiving sEPSCs (left), the frequency of sEPSCs (middle), and amplitude of sEPSCs (right) across the three culture conditions. Sample sizes represent individual neurons recorded from 13 cultures across three cell lines (P + L neurons: 92 ± 3.3 days), 12 cultures across two cell lines (on rat astrocytes: neurons 93 ± 4.9 days, co-cultured for 51 ± 2.9 days), and five cultures across two cell lines (on human astrocytes: neurons 84 ± 7.6 days, astrocytes 120 ± 9.5 days, co-cultured for 44 ± 7.7 days). $^{*}p < 0.05$, $^{**}p < 0.01$.

To evaluate whether the iPSC-astrocytes could also influence synaptic network maturation, the presence of structural pre-synaptic sites was confirmed in each of the three culture conditions (Figure 5A), whereupon functional synaptic transmission was assessed by recording sEPSCs (Figure 5B). The proportion of neurons receiving synaptic inputs was significantly enhanced by co-culture with either rat astrocytes or human iPSC-astrocytes (43% on P + L, 65% on rat astrocytes, and 81% on human astrocytes; p = 0.004, chi-square contingency test; Figure 5C). The frequency of sEPSCs was enhanced by co-culture with human astrocytes (0.15 ± 0.04 Hz on P + L, 0.23 ± 0.07 Hz on rat astrocytes, and 0.25 ± 0.08 Hz on human astrocytes; p = 0.050, unpaired Kruskal-Wallis with Dunn's multiple comparisons test; Figure 5C). The amplitude of sEPSCs was similar between conditions (p = 0.470, unpaired Kruskall-Wallis test). Together, these data demonstrate that our cortically derived iPSC-astrocytes are capable of exhibiting pro-maturational effects upon cortically derived iPSC-neurons.

Pro-maturational iPSC-Astrocytes Express Extracellular Proteins that Interact with Neuronal Synaptic Proteins

To investigate the biological processes underlying these pro-maturational effects upon neurons, we compared the differences in gene expression between our iPSC-astrocytes and iPSC-astrocytes previously shown to be unable to promote synaptic network maturation (Lischka et al., 2018) with those genes differently expressed between adult and fetal astrocytes from human brain tissue (Zhang et al., 2016; Figure 6A; DESeq2, false discovery rate [FDR] <0.05). Consistent with their pro-maturational effects, a statistically significant concordance was only found

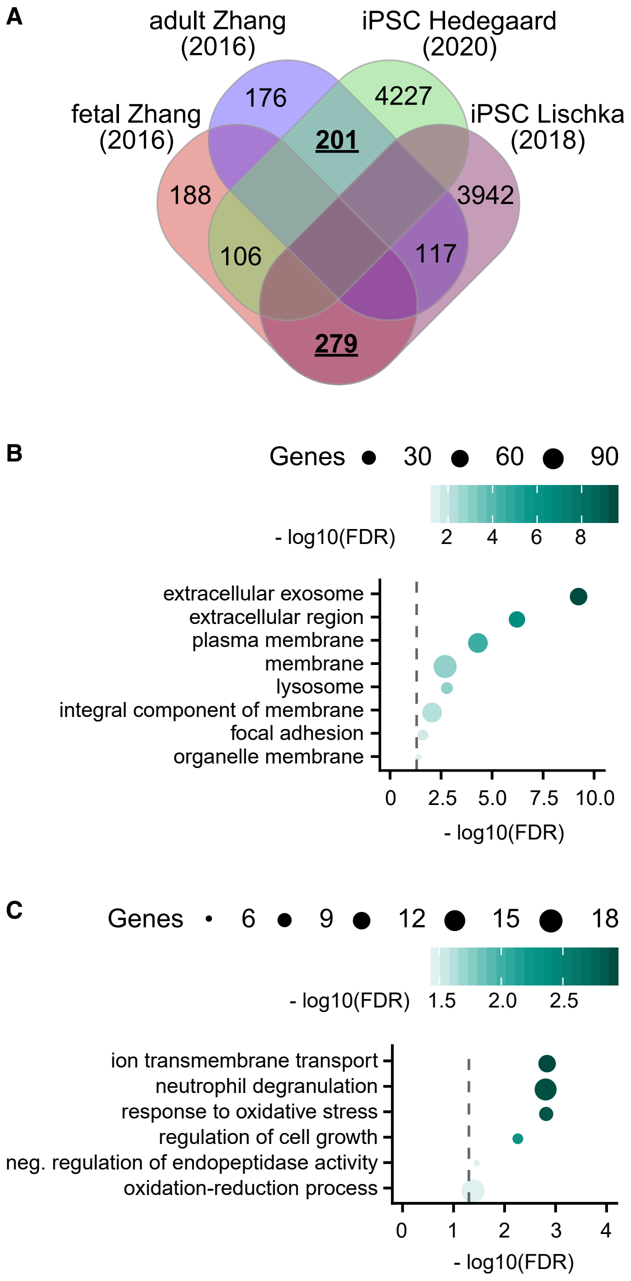

Figure 6. Transcriptomic Features of iPSC-Astrocytes that Mediate Pro-maturational Effects upon Cortical Neurons

(A) Venn diagram showing overlap between genes that are highly expressed in either fetal and adult human astrocytes, identified in Zhang et al. (2016) and genes that were differentially expressed in the iPSC-astrocytes presented in this study (iPSC Hedegaard) or iPSC-astrocytes known to not promote neuronal maturation (iPSC Lischka). Overlapping gene sets that are larger than expected by chance are underlined and in bold ($p < 0.05$).

(B and C) Overrepresented cellular components (B) and biological processes (C) among the set of 201 genes with higher expression in both primary adult astrocytes (compared with fetal) and iPSC Hedegaard astrocytes (compared with iPSC Lischka). Circle size indicates the number of genes annotated to each Gene Ontology (GO) term; color reflects the log10 transformed false discovery rate (FDR) and dashed line indicates a FDR of 0.05.

between (1) genes whose expression was higher in both the primary adult astrocytes and higher in our iPSC-astrocytes (n = 201 genes, $p = 1.28 \times 10^{-9}$, hypergeometric test), and (2) genes whose expression was higher in both the fetal astrocytes and higher in the Lischka et al. (2018) iPSC-astrocytes (n = 279 genes, $p = 6.85 \times 10^{-30}$, hypergeometric test). We did not find more differentially expressed genes than expected by chance in any other gene set overlaps ($p > 0.05$; Figure 6A). Among the 201 genes that were higher in both the primary adult astrocytes and our iPSC-astrocytes, we found an overrepresentation of proteins located extracellularly, in membranes and/or in lysosomal compartments (Figure 6B), with overrepresented roles including ion transmembrane transport, neutrophil degranulation, and response to oxidative stress (Figure 6C, hypergeometric test, FDR <0.05). Meanwhile, among the 279 genes higher in both the primary fetal astrocytes and the Lischka et al. (2018) iPSC-astrocytes, we found an overrepresentation of proteins associated with chromosomes, mitotic spindles, and the nucleoplasm, with overrepresented roles including cell cycle and cell proliferation processes (Figure S5, hypergeometric test, FDR <0.05).

Given the overrepresentation of genes associated with membrane and extracellular components in both the primary adult astrocytes and our iPSC-astrocytes, we hypothesized that these genes might be important for interactions with neurons and synapses. To explore their potential role, we extracted the subset of the 201 genes annotated as encoding extracellular proteins (n = 84 genes) and confirmed, using a human atlas of gene expression patterns, that these exhibit highest expression in mature astrocytes (Darmanis et al., 2015; Figure 7A). Using Gene Ontology (GO) annotations, we then identified all genes whose proteins are annotated as “pre-synaptic” and “post-synaptic” and corroborated their overall neuron-specific expression (Darmanis et al., 2015; Figure 7A). Finally, we asked if the identified subset of astrocytic extracellular proteins are predicted to engage in protein-protein interactions with pre- and post-synaptic neuronal proteins (see Supplemental Experimental Procedures). This revealed a statistical overrepresentation of interactions between the astrocytic extracellular proteins and both the neuronal pre-synaptic and post-synaptic proteins ($p = 0.0027$ and $p = 0.0055$, respectively; via randomizations; Figure 7B; expanded subset of the protein-protein interaction network is shown in Figure S6). Taken together, these transcriptomic analyses support the idea that the iPSC-astrocytes can engage in synapse-associated extracellular signaling to mediate pro-maturational effects upon neurons.

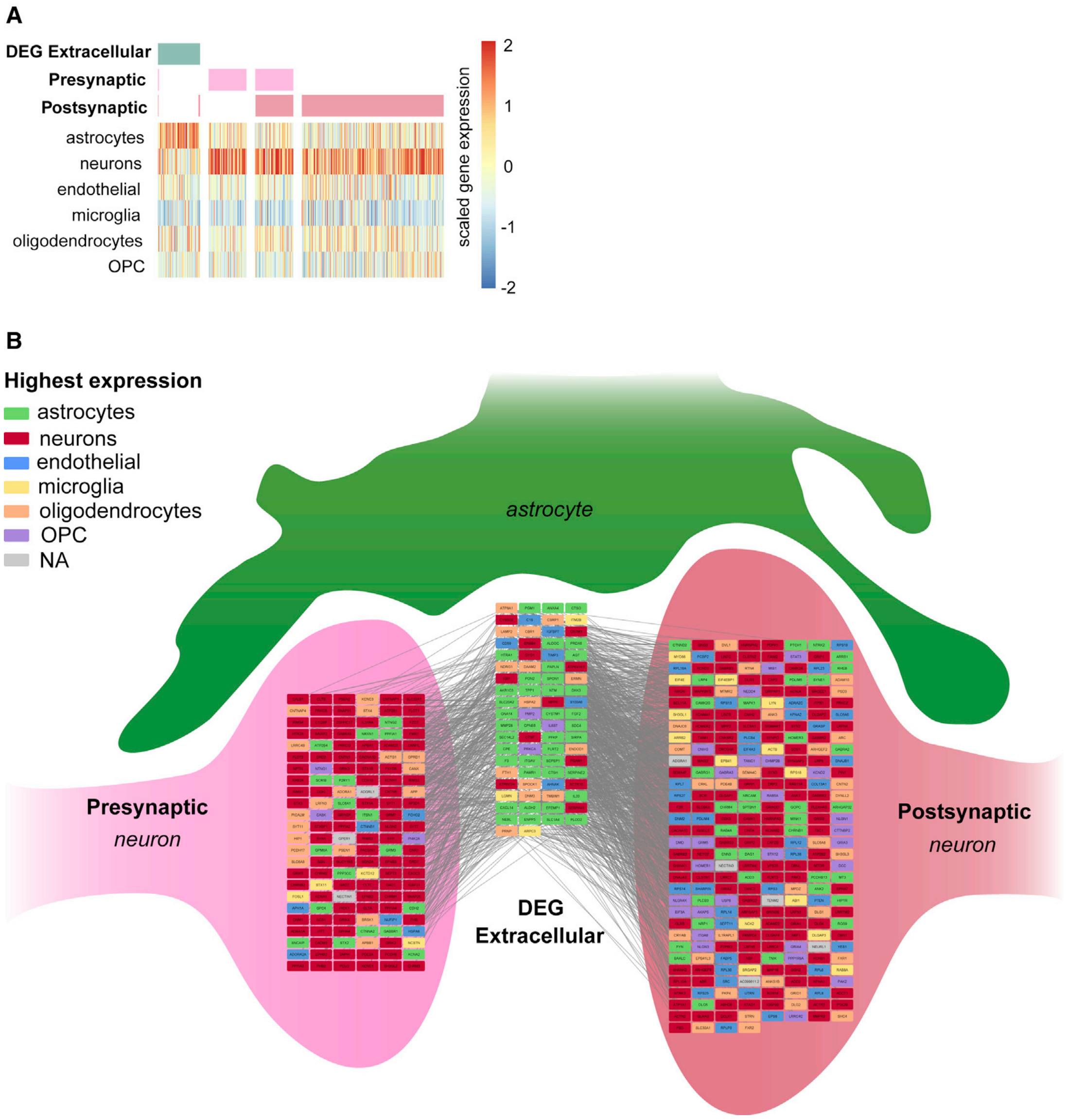

Figure 7. Pro-maturational Effects of iPSC-Astrocytes Are Associated with the Expression of Synapse-Interacting Extracellular Proteins

(A) Of the 201 differentially expressed genes that overlap between primary mature astrocytes and the iPSC-astrocytes generated in this study, 84 encode extracellular proteins (annotated to GO:0070062, GO:0005576, or GO:0005615, shown in green). Genes encoding pre- and post-synaptic proteins extracted from annotated datasets (shown in light and dark pink, respectively). The expression patterns of extracellular differentially expressed genes (DEGs) and pre- and post-synaptic genes were corroborated across different brain cell types according to Darmanis et al. (2015).

(B) Protein-protein interaction network for the differentially expressed extracellular astrocyte genes, and pre- and post-synaptic genes. Nodes represent genes and are color-coded according to the cell type with highest expression. Each line denotes a known protein-protein interaction between the protein products of these genes. See Figure S6 for a more detailed representation of the network. OPC, oligodendrocyte precursor cells.

DISCUSSION

Here, we establish a human cortical iPSC system for investigating astrocyte-to-neuron interactions. Our approach uses dual SMAD inhibition to generate a common pool of OTX2^{+} cortical radial glia progenitors, from which both astrocytes and neurons could then be independently generated. This strategy reflects *in vivo* cortical development,

where neurons and astrocytes derive from a common progenitor pool (Rowitch and Kriegstein, 2010), and aligns with the idea that the regional identity of a progenitor influences the identity of its progeny (Nadadhur et al., 2018). The resulting method afforded the opportunity to examine astrocyte-neuron interactions in cells from a defined, common lineage. As with iPSC-astrocytes generated via other protocols, transcriptomic analyses indicated that our cortical iPSC-astrocytes are relatively immature compared with adult cortical astrocytes from human brain (Lin et al., 2018; Lischka et al., 2018; Santos et al., 2017; Tcw et al., 2017; Zhang et al., 2016). Nevertheless, at the stages examined, the cortical iPSC-astrocytes exhibited key molecular and functional features, including the expression of well-known astrocyte-specific genes, relevant intrinsic membrane properties, gap-junction coupling, and mechanisms for regulating extracellular molecules.

A major focus was to examine the capacity of the iPSC-astrocytes to engage in intercellular signaling with iPSC-derived neurons, because the importance of these interactions is being increasingly recognized in development and in disorders such as epilepsy and Alzheimer's disease (Dossi et al., 2018; Wetherington et al., 2008). Our cortically derived iPSC-astrocytes exhibited rapid increases in intracellular Ca^{2+} in response to neurotransmitters such as glutamate and ATP, as has been shown for other populations of iPSC-astrocytes (Canals et al., 2018; Krencik et al., 2011; Santos et al., 2017; Tchieu et al., 2019; Tcw et al., 2017). We have demonstrated that the iPSC-astrocytes are also able to rapidly signal back to neurons. Brief and selective optogenetic stimulation of the iPSC-astrocytes resulted in the modulation of ongoing synaptic transmission between nearby neurons, in a mGluR-dependent manner. This is analogous to forms of activity-dependent gliotransmission described in rodent cortex and in biopsies from human cortex (Navarrete et al., 2013; Perea et al., 2014; Sasaki et al., 2012). Although it is widely accepted that astrocytes engage in activity-dependent signaling, there is debate regarding the conditions under which gliotransmission occurs and the underlying cellular mechanisms (Fiacco and McCarthy, 2018; Savtchouk and Volterra, 2018). The current study therefore provides a human system in which to examine these processes, plus the opportunity to explore this in a disease context. Our data derived primarily from three healthy donors, but also included a small number of astrocyte cultures from a cell line derived from a donor diagnosed with sporadic Alzheimer's disease (see Supplemental Experimental Procedures). In the future, it would be interesting to examine how astrocyte-to-neuron signaling relates to the state of the human astrocyte, the developmental stage of the network, and to neurological disorders such as Alzheimer's disease.

Astrocyte-to-neuron interactions were also captured over longer timescales. Our long-term co-culture experiments revealed pro-maturational effects of cortical iPSC-astrocytes upon cortical iPSC-neurons. The neurons exhibited enhanced electrical excitability and synaptic network activity, equivalent to the effects observed following co-culture with rodent cortical astrocytes. These findings extend recent reports that iPSC-astrocytes of different origin can exhibit certain pro-maturational effects upon co-cultured neurons (Canals et al., 2018; Kayama et al., 2018; Klapper et al., 2019; Tchieu et al., 2019; VanderWall et al., 2019), but can fail to exhibit such effects (Lischka et al., 2018). By combining our observations with transcriptomic analyses, we linked the pro-maturational effects on neuron to astrocyte maturity and the expression of astrocytic genes encoding extracellular proteins. More specifically, our approach identified candidate genes and pathways relevant to extracellular interactions with neuronal pre- and post-synaptic proteins. These included: ITGAV, an essential protein for neuron-glial attachment during cortical development, which influences synapse structure and maturation (Anton et al., 1999; Park and Goda, 2016); SLC1A4, a Na^{+}-dependent amino acid transporter that plays a role in modulating glutamatergic transmission (Kaplan et al., 2018); NTM, ENPP5, EFEMP1, and SIRPA, all glycoproteins involved in cell adhesion, cell-to-cell recognition, and EFEMP1 and SIRPA have been associated with neurite outgrowth and synaptic development (Vukovic et al., 2009; Wang and Pfenninger, 2006). Future work could use targeted manipulations to investigate the relevance of these astrocytic genes in terms of the formation and maintenance of human cortical neuronal networks.

In conclusion, we provide a detailed description of human cortical iPSC-derived astrocytes, which can reproduce many of the key astrocyte-to-neuron signaling processes observed in animal systems. The iPSC-astrocytes are able to signal bidirectionally with iPSC-neurons, by rapidly responding to neurotransmitters and actively modulating ongoing neuronal activity. Interactions over longer timescales result in pro-maturational effects upon cortical neuronal networks, which are associated with synapse-related signaling between the astrocytes and neurons. This work provides a foundation for further investigations into astrocyte-neuron interactions in human health and disease.

EXPERIMENTAL PROCEDURES

Human iPSC Lines

The iPSC lines were derived from human skin biopsy fibroblasts following signed informed consent, with approval from the UK NHS Research Ethics Committee (REC, 13/SC/0179) and were derived as part of the IMI-EU sponsored StemBANCC consortium. Further information in Supplemental Experimental Procedures.

Differentiation of iPSCs to Cortical Neurons and Astrocytes

iPSCs were differentiated into cortical neurons using the protocol detailed in Volpato et al. (2018) ("Standard operating procedure for cortical differentiation of hiPSCs") and based on Shi et al. (2012). Human astrocytes were differentiated from cortical progenitors using the Astrocyte Differentiation and Maturation kits available from STEMCELL Technologies (nos. 08540 and 08550, respectively). In brief, cortical rosettes at day 20–25 of neural induction were treated with the Astrocyte Differentiation medium for 20 days (until day 40–45), during which cells were expanded three times using Accutase (Sigma), and re-plated at a density of 1×10^5 cells/cm^2 on Matrigel-coated six-well plates. Subsequently the medium was switched to the Astrocyte Maturation medium for a further 15 days (until day 55–60), and cells were expanded three times as described above. At the transition stage between different media, cultures were cryopreserved in their current medium + 10% DMSO. To maintain astrocytes beyond day 60, cells were cultured in an astrocyte maintenance medium, which was replaced every 2–3 days. Further details in Supplemental Experimental Procedures.

Establishment of Co-cultures

For co-cultures of human iPSC-neurons with either human or rat astrocytes, the astrocytes were seeded onto Matrigel-coated (Scientific Lab Supplies) glass coverslips and allowed to reach confluence, before 100,000–150,000 cortical neurons/coverslip were seeded on top. To facilitate selective patching of neurons in co-cultures, iPSC-neurons were transduced with a lentivirus expressing mKate2 under the control of the CamKIIα promoter (see Supplemental Experimental Procedures) prior to co-culture. At the time of co-culture, human astrocytes were typically ~70–80 days old and neurons were ~35–45 days old, and co-cultures were always generated with cells from the same donor. For optogenetic experiments, astrocytes were transduced with a lentivirus expressing humanized ChR2 (hChR2) under the control of a CAG promoter (see Supplemental Experimental Procedures) before the addition of neurons. Co-cultures were maintained in a modified neuronal maintenance medium, containing (50% vol/vol Neurobasal-A, 50% vol/vol DMEM/F12 Glutamax medium with 1× N2, 1× B27 + vitamin A, 2.5 μg/mL insulin, 1 mM L-glutamine, 0.5× non-essential amino acids, 0.5 mM sodium pyruvate, 55 μM β-mercaptoethanol, 50 U/mL penicillin, and 50 mg/mL streptomycin), with medium changes every 2–3 days and supplemented with 2% fetal bovine serum and laminin at 10 μg/mL once a week.

Electrophysiological Recordings

Whole-cell patch-clamp recordings were performed with thin-wall borosilicate glass pipettes (resistances of 5–8 MΩ for neurons and 7–12 MΩ for astrocytes), back-filled with intracellular solution (140 mM K^+ gluconate, 6 mM NaCl, 1 mM EGTA, 10 mM HEPES, 4 mM MgATP, and 0.4 mM Na_3GTP). For biocytin filling, 5 μg/mL biocytin (Sigma) was dissolved in the internal solution and left to disperse for at least 15 min. During recordings, the cultures were constantly perfused with external solution (140 mM NaCl, 5 mM KCl, 2 mM $CaCl_2$, 10 mM HEPES, and 10 mM glucose) at a rate of 2 mL/min, heated to 33–35°C. Voltage-gated currents were elicited from cells clamped at −70 mV in voltage-clamp mode, using 10 mV voltage step protocols from −90 to +20 mV (neurons) or −140 to +30 mV (astrocytes). Action potentials were elicited in current-clamp mode by the injection of 200 ms square current pulses (5 or 10 pA steps), from a baseline Vm of −70 mV and were counted if their peak was greater than −10 mV. sEPSCs were recorded in voltage-clamp mode at the reversal potential of $GABA_A$ receptors (E_{GABA}; −70 mV after +14mV junction potential correction). For further details of electrophysiological data analysis, see Supplemental Experimental Procedures.

Optogenetic Stimulation

Stimulation of ChR2-expressing astrocytes and simultaneous patch-clamp recordings of sEPSCs from co-cultured neurons were performed using a Rebel Star Royal Blue LED (447.5 nm wavelength, Luxeon). These neurons were clamped at −70 mV in the presence of picrotoxin (PTX). A 2 min baseline was recorded, then a 50 s train of 245 ms LED pulses was delivered at 2–4 Hz from beneath the culture. LED intensities ranged from 0.3 to 2.7 mW/mm^2.

RNA Sequencing and Quantification

Nine samples of iPSC-astrocytes (three cultures from each of three cell lines; mean age, 96 ± 3.3 days) were prepared for sequencing using the HiSeq 3000/4000 SBS Kit. Approximately 30 million 75 bp paired-end reads were obtained per sample. For initial quality control, FASTQC (Andrews, 2010) and MultiQC were used to summarize the results (Ewels et al., 2016). Kallisto for Linux version 0.43.1 (Bray et al., 2016) was used to quantify transcript abundances. For further details on differential expression analysis, GO analysis and protein-protein interaction analysis, see Supplemental Experimental Procedures.

Experimental Design and Statistical Analysis

All data were collected and analyzed by the same person (A.H.). All statistical analysis was performed using Prism software, version 6 (GraphPad). Bars on graphs represent means and error bars indicate ± standard error of the mean. Non-parametric statistical tests were used on all datasets, because ranks and medians are more robust to outliers. The number of cells, fields of view, and regions of interest evaluated in each experiment are reported in the figures and the legends. Datasets were regarded as unpaired, and two-tailed tests were performed. The relevant statistical tests, as well as corrections for multiple comparisons, are indicated after each reported p value. Statistical significance is reported at the following levels: *$p < 0.05$, **$p < 0.01$, ***$p < 0.001$ in the figures, with precise p values given in the text.

Accession Numbers

The accession number for the transcriptomic datasets reported in this paper is GEO: GSE149598.

SUPPLEMENTAL INFORMATION

Supplemental Information can be found online at https://doi.org/10.1016/j.stemcr.2020.05.003.

AUTHOR CONTRIBUTIONS

Designed research: A.H., S.E.N., C.W., and C.J.A. designed the research. A.H. and J.M.-S. performed the research. E.S.W. and S.E.N. contributed unpublished reagents/analytical tools. A.H.

and J.M.-S. analyzed the data. A.H., J.M.-S., and C.J.A. wrote the paper. The manuscript was approved by all of the authors.

ACKNOWLEDGMENTS

The research leading to these results received support from ERC grant agreement no. 617670 and the Innovative Medicines Initiative Joint Undertaking under grant agreement no. 115439, resources of which were composed of financial contribution from the European Union's Seventh Framework Program (FP7/2007-2013) and EFPIA companies' in kind contribution. The project was supported by funding from BBSRC project BB/S007938/1 and Alzheimer's Research UK (ARUK-PhD2014-25 and ARUK-PPG2018B-014). We thank Dr. Martin Doughty and Clifton Dalgard for sharing their published transcriptomic dataset.

Received: March 6, 2020
Revised: May 4, 2020
Accepted: May 4, 2020
Published: June 4, 2020

REFERENCES

Aguado, F., Espinosa-Parrilla, J.F., Carmona, M.A., and Soriano, E. (2002). Neuronal activity regulates correlated network properties of spontaneous calcium transients in astrocytes in situ. J. Neurosci. *22*, 9430–9444.

Allen, N.J., and Eroglu, C. (2017). Cell biology of astrocyte-synapse interactions. Neuron *96*, 697–708.

Andrews, S. (2010). FastQC: A Quality Control Tool for High Throughput Sequence Data. http://www.bioinformatics.babraham.ac.uk/projects/fastqc.

Anton, E.S., Kreidberg, J.A., and Rakic, P. (1999). Distinct functions of α3 and α(v) integrin receptors in neuronal migration and laminar organization of the cerebral cortex. Neuron *22*, 277–289.

Bardy, C., Van Den Hurk, M., Kakaradov, B., Erwin, J., Jaeger, B., Hernandez, R., Eames, T., Paucar, A., Gorris, M., Marchand, C., et al. (2016). Predicting the functional states of human iPSC-derived neurons with single-cell RNA-seq and electrophysiology. Mol. Psychiatry *21*, 1573–1588.

Barnabé-Heider, F., Wasylnka, J.A., Fernandes, K.J.L., Porsche, C., Sendtner, M., Kaplan, D.R., and Miller, F.D. (2005). Evidence that embryonic neurons regulate the onset of cortical gliogenesis via cardiotrophin-1. Neuron *48*, 253–265.

Bray, N.L., Pimentel, H., Melsted, P., and Pachter, L. (2016). Near-optimal probabilistic RNA-seq quantification. Nat. Biotechnol. *34*, 525–527.

Cahoy, J., Emery, B., Kaushal, A., Foo, L., Zamanian, J., Christopherson, K., Xing, Y., Lubischer, J., Krieg, P., Krupenko, S., et al. (2008). A transcriptome database for astrocytes, neurons, and oligodendrocytes: a new resource for understanding brain development and function. J. Neurosci. *28*, 264–278.

Canals, I., Ginisty, A., Quist, E., Timmerman, R., Fritze, J., Miskinyte, G., Monni, E., Hansen, M.G., Hidalgo, I., Bryder, D., et al. (2018). Rapid and efficient induction of functional astrocytes from human pluripotent stem cells. Nat. Methods *15*, 693–696.

Di Castro, M.A., Chuquet, J., Liaudet, N., Bhaukaurally, K., Santello, M., Bouvier, D., Tiret, P., and Volterra, A. (2011). Local Ca^{2+} detection and modulation of synaptic release by astrocytes. Nat. Neurosci. *14*, 1276–1284.

Chambers, S.M., Fasano, C.A., Papapetrou, E.P., Tomishima, M., Sadelain, M., and Studer, L. (2009). Highly efficient neural conversion of human ES and iPS cells by dual inhibition of SMAD signaling. Nat. Biotechnol. *27*, 275–280.

Dallas, M., Boycott, H.E., Atkinson, L., Miller, A., Boyle, J.P., Pearson, H.A., and Peers, C. (2007). Hypoxia suppresses glutamate transport in astrocytes. J. Neurosci. *27*, 3946–3955.

Darmanis, S., Sloan, S.A., Zhang, Y., Enge, M., Caneda, C., Shuer, L.M., Gephart, M.G.H., Barres, B.A., and Quake, S.R. (2015). A survey of human brain transcriptome diversity at the single cell level. Proc. Natl. Acad. Sci. U S A *112*, 7285–7290.

Djukic, B., Casper, K.B., Philpot, B.D., Chin, L.-S., and McCarthy, K.D. (2007). Conditional knock-out of Kir4.1 leads to glial membrane depolarization, inhibition of potassium and glutamate uptake, and enhanced short-term synaptic potentiation. J. Neurosci. *27*, 11354–11365.

Dossi, E., Vasile, F., and Rouach, N. (2018). Human astrocytes in the diseased brain. Brain Res. Bull. *136*, 139–156.

Ewels, P., Magnusson, M., Lundin, S., and Käller, M. (2016). MultiQC: summarize analysis results for multiple tools and samples in a single report. Bioinformatics *32*, 3047–3048.

Fellin, T., and Carmignoto, G. (2004). Neurone-to-astrocyte signalling in the brain represents a distinct multifunctional unit. J. Physiol. *559*, 3–15.

Fiacco, T.A., and McCarthy, K.D. (2018). Multiple lines of evidence indicate that gliotransmission does not occur under physiological conditions. J. Neurosci. *38*, 3–13.

Kaplan, E., Zubedat, S., Radzishevsky, I., Valenta, A.C., Rechnitz, O., Sason, H., Sajrawi, C., Bodner, O., Konno, K., Esaki, K., et al. (2018). ASCT1 (Slc1a4) transporter is a physiologic regulator of brain D-serine and neurodevelopment. Proc. Natl. Acad. Sci. U S A *115*, 9628–9633.

Kayama, T., Suzuki, I., Odawara, A., Sasaki, T., and Ikegaya, Y. (2018). Temporally coordinated spiking activity of human induced pluripotent stem cell-derived neurons co-cultured with astrocytes. Biochem. Biophys. Res. Commun. *495*, 1028–1033.

Klapper, S.D., Garg, P., Dagar, S., Lenk, K., Gottmann, K., and Nieweg, K. (2019). Astrocyte lineage cells are essential for functional neuronal differentiation and synapse maturation in human iPSC-derived neural networks. Glia *67*, 1893–1909.

Krencik, R., Weick, J.P., Liu, Y., Zhang, Z.-J., and Zhang, S.-C. (2011). Specification of transplantable astroglial subtypes from human pluripotent stem cells. Nat. Biotechnol. *29*, 528–534.

Liddelow, S.A., and Barres, B.A. (2017). Reactive astrocytes: production, function, and therapeutic potential. Immunity *46*, 957–967.

Lin, Y.T., Seo, J., Gao, F., Feldman, H.M., Wen, H.L., Penney, J., Cam, H.P., Gjoneska, E., Raja, W.K., Cheng, J., et al. (2018). APOE4 causes widespread molecular and cellular alterations associated with Alzheimer's disease phenotypes in human iPSC-derived brain cell types. Neuron *98*, 1141–1154.e7.

Lischka, F.W., Efthymiou, A., Zhou, Q., Nieves, M.D., McCormack, N.M., Wilkerson, M.D., Sukumar, G., Dalgard, C.L., and Doughty, M.L. (2018). Neonatal mouse cortical but not isogenic human astrocyte feeder layers enhance the functional maturation of induced pluripotent stem cell-derived neurons in culture. Glia *66*, 725–748.

Liu, H., and Zhang, S.-C. (2011). Specification of neuronal and glial subtypes from human pluripotent stem cells. Cell. Mol. Life Sci. *68*, 3995–4008.

Ludwin, S.K., Kosek, J.C., and Eng, L.F. (1976). The topographical distribution of S-100 and GFA proteins in the adult rat brain: an immunohistochemical study using horseradish peroxidase-labelled antibodies. J. Comp. Neurol. *165*, 197–207.

Nadadhur, A.G., Leferink, P.S., Holmes, D., Hinz, L., Cornelissen-Steijger, P., Gasparotto, L., and Heine, V.M. (2018). Patterning factors during neural progenitor induction determine regional identity and differentiation potential in vitro. Stem Cell Res. *32*, 25–34.

Nagel, G., Szellas, T., Huhn, W., Kateriya, S., Adeishvili, N., Berthold, P., Ollig, D., Hegemann, P., and Bamberg, E. (2003). Channelrhodopsin-2, a directly light-gated cation-selective membrane channel. Proc. Natl. Acad. Sci. U S A *100*, 13940–13945.

Navarrete, M., Perea, G., Maglio, L., Pastor, J., García de Sola, R., and Araque, A. (2013). Astrocyte calcium signal and gliotransmission in human brain tissue. Cereb. Cortex *23*, 1240–1246.

Panatier, A., Vallée, J., Haber, M., Murai, K.K., Lacaille, J.C., and Robitaille, R. (2011). Astrocytes are endogenous regulators of basal transmission at central synapses. Cell *146*, 785–798.

Park, Y.K., and Goda, Y. (2016). Integrins in synapse regulation. Nat. Rev. Neurosci. *17*, 745–756.

Perea, G., and Araque, A. (2010). GLIA modulates synaptic transmission. Brain Res. Rev. *63*, 93–102.

Perea, G., Yang, A., Boyden, E.S., and Sur, M. (2014). Optogenetic astrocyte activation modulates response selectivity of visual cortex neurons in vivo. Nat. Commun. *5*, 3262.

Porter, J.T., and McCarthy, K.D. (1996). Hippocampal astrocytes in situ respond to glutamate released from synaptic terminals. J. Neurosci. *16*, 5073–5081.

Rowitch, D.H., and Kriegstein, A.R. (2010). Developmental genetics of vertebrate glial-cell specification. Nature *468*, 214–222.

Roybon, L., Lamas, N.J., Garcia-Diaz, A., Yang, E.J., Sattler, R., Jackson-Lewis, V., Kim, Y.A., Kachel, C.A., Rothstein, J.D., Przedborski, S., et al. (2013). Human stem cell-derived spinal cord astrocytes with defined mature or reactive phenotypes. Cell Rep. *4*, 1035–1048.

Santos, R., Vadodaria, K.C., Jaeger, B.N., Mei, A., Lefcochilos-Fogelquist, S., Mendes, A.P.D., Erikson, G., Shokhirev, M., Randolph-Moore, L., Fredlender, C., et al. (2017). Differentiation of inflammation-responsive astrocytes from glial progenitors generated from human induced pluripotent stem cells. Stem Cell Reports *8*, 1757–1769.

Sasaki, T., Beppu, K., Tanaka, K.F., Fukazawa, Y., Shigemoto, R., and Matsui, K. (2012). Application of an optogenetic byway for perturbing neuronal activity via glial photostimulation. Proc. Natl. Acad. Sci. U S A *109*, 20720–20725.

Savtchouk, I., and Volterra, A. (2018). Gliotransmission: beyond black-and-white. J. Neurosci. *38*, 14–25.

Serio, A., Bilican, B., Barmada, S.J., Ando, D.M., Zhao, C., Siller, R., Burr, K., Haghi, G., Story, D., Nishimura, A.L., et al. (2013). Astrocyte pathology and the absence of non-cell autonomy in an induced pluripotent stem cell model of TDP-43 proteinopathy. Proc. Natl. Acad. Sci. U S A *110*, 4697–4702.

Shaltouki, A., Peng, J., Liu, Q., Rao, M.S., and Zeng, X. (2013). Efficient generation of astrocytes from human pluripotent stem cells in defined conditions. Stem Cells *31*, 941–952.

Shi, Y., Kirwan, P., and Livesey, F.J. (2012). Directed differentiation of human pluripotent stem cells to cerebral cortex neurons and neural networks. Nat. Protoc. *7*, 1836–1846.

Sloan, S.A., Darmanis, S., Huber, N., Khan, T.A., Birey, F., Caneda, C., Reimer, R., Quake, S.R., Barres, B.A., and Paşca, S.P. (2017). Human astrocyte maturation captured in 3D cerebral cortical spheroids derived from pluripotent stem cells. Neuron *95*, 779–790.e6.

Tchieu, J., Calder, E.L., Guttikonda, S.R., Gutzwiller, E.M., Aromolaran, K.A., Steinbeck, J.A., Goldstein, P.A., and Studer, L. (2019). NFIA is a gliogenic switch enabling rapid derivation of functional human astrocytes from pluripotent stem cells. Nat. Biotechnol. *37*, 267–275.

Tcw, J., Wang, M., Pimenova, A.A., Bowles, K.R., Hartley, B.J., Lacin, E., Machlovi, S., Abdelaal, R., Karch, C.M., Phetnani, H., et al. (2017). An efficient platform for astrocyte differentiation from human induced pluripotent stem cells. Stem Cell Reports *9*, 600–614.

Theis, M., Söhl, G., Eiberger, J., and Willecke, K. (2005). Emerging complexities in identity and function of glial connexins. Trends Neurosci. *28*, 188–195.

VanderWall, K.B., Vij, R., Ohlemacher, S.K., Sridhar, A., Fligor, C.M., Feder, E.M., Edler, M.C., Baucum, A.J., Cummins, T.R., and Meyer, J.S. (2019). Astrocytes regulate the development and maturation of retinal ganglion cells derived from human pluripotent stem cells. Stem Cell Reports *12*, 201–212.

Verkhratsky, A., and Nedergaard, M. (2014). Astroglial cradle in the life of the synapse. Philos. Trans. R. Soc. B Biol. Sci. *369*, 20130595.

Verkhratsky, A., and Nedergaard, M. (2018). Physiology of astroglia. Physiol. Rev. *98*, 239–389.

Volpato, V., Smith, J., Sandor, C., Ried, J.S., Baud, A., Handel, A., Newey, S.E., Wessely, F., Attar, M., Whiteley, E., et al. (2018). Reproducibility of molecular phenotypes after long-term differentiation to human iPSC-derived neurons: a multi-site omics study. Stem Cell Reports *11*, 897–911.

Vukovic, J., Ruitenberg, M.J., Roet, K., Franssen, E., Arulpragasam, A., Sasaki, T., Verhaagen, J., Harvey, A.R., Busfield, S.J., and Plant, G.W. (2009). The glycoprotein fibulin-3 regulates morphology and motility of olfactory ensheathing cells *in vitro*. Glia *57*, 424–443.

Wang, X.X., and Pfenninger, K.H. (2006). Functional analysis of SIRPalpha in the growth cone. J. Cell Sci. *119*, 172–183.

Wetherington, J., Serrano, G., and Dingledine, R. (2008). Astrocytes in the epileptic brain. Neuron *58*, 168–178.

Zhang, Y., Sloan, S.A., Clarke, L.E., Caneda, C., Plaza, C.A., Blumenthal, P.D., Vogel, H., Steinberg, G.K., Edwards, M.S.B., Li, G., et al. (2016). Purification and characterization of progenitor and mature human astrocytes reveals transcriptional and functional differences with mouse. Neuron *89*, 37–53.

Stem Cell Reports, Volume *15*

Supplemental Information

Pro-maturational Effects of Human iPSC-Derived Cortical Astrocytes upon iPSC-Derived Cortical Neurons

Anne Hedegaard, Jimena Monzón-Sandoval, Sarah E. Newey, Emma S. Whiteley, Caleb Webber, and Colin J. Akerman

Supplemental experimental procedures

Maintenance of human iPSC lines

iPSC lines were reprogrammed using the CytoTune-iPS 2.0 Sendai Reprogramming Kit (A16517, Thermo Fisher Scientific; (Volpato et al., 2018)). The vast majority of data was collected from healthy control cell lines: SBAd03, Clones# 01 and 05, female aged 31 years; SBAd02, Clone# 01, male aged 51 years; SFC841-03, Clone# 01, male aged 36 years. In assessing the potential for our protocol to differentiate iPSCs to astrocytes, some differentiations were also performed using a cell line from a patient diagnosed with sporadic Alzheimer's disease (AD): SFC042-03, Clone# 01, female aged 67 years. Whilst we had limited statistical power to compare across cell lines, we did not observe any difference in the ability of this cell line to differentiate into astrocytes. This line contributed 4 data points to the plots in Figure 2C, 2 data points in Figure 2G, 3 data points in Figure S3 and one field of view in Figure 3A. The human iPSCs were maintained under feeder-free conditions on geltrex-coated (Gibco) six-well plates (Corning) in mTeSR1 (STEMCELL Technologies) and passaged at a rate of 1:3 with enzymatic dissociation using Versene-EDTA when they reached 90% confluency.

Differentiation of iPSCs to cortical neurons

In preparation for neural induction, following a modified Shi et al. (2012) protocol (Shi et al., 2012), the iPSCs were passaged using Versene-EDTA at a ratio of 2:1 to ensure 100% confluency. The next day (Induction day 0), the culture medium was changed to neural induction medium (NIM) consisting of neural maintenance media (NMM: 50% vol/vol Neurobasal, 50% vol/vol DMEM/F12 Glutamax medium with 1x N2, 1x B27 + Vitamin A, 2.5 µg/mL insulin (Sigma), 1 mM L-glutamine, 0.5x non-essential amino acids (NEAAs), 0.5 mM Sodium Pyruvate (Sigma), 55 µM β-mercaptoethanol, 50 U/mL penicillin and 50 mg/mL streptomycin), supplemented with dual SMAD inhibiting factors: 10 µM SB431542 (Tocris) and 1 µM dorsomorphin (Tocris). NIM was changed daily for 12 days as the cells were forming a dense neuroepithelial sheet. To lift the sheet on day 13, it was scored into a grid using a sterile needle and incubated at 37°C with 200 µL freshly filtered 10 mg/mL Dispase (Gibco) added to the media. Lifting was encouraged by frequently tapping the plates over 10-15 min of incubation and cells were collected using a stripette to gently transfer them without breaking up the sheet too much into 10 mL of NMM. To wash out the Dispase, cells were allowed to pellet by gravity, media removed and this wash-step repeated. Cell clumps were plated in NIM supplemented with 10 µM Y-27632 (Tocris) onto plates pre-coated for >4 hours with 15 µg/mL laminin (Life Technologies) and left to adhere overnight. The medium was changed 24 hours later to NMM supplemented with 20 ng/mL FGF2 (Peprotech) and changed every day for 4 days to promote/encourage rosette formation.

Rosettes were then maintained in NMM over the next 10-15 days, during which they were expanded onto laminin-coated wells and cleaned up 2-5 times by selective lifting with Dispase as described above, but without the needle-scoring. Onset of neurogenesis occurred around day 20-25, at which point cultures were dissociated to single cells using Accutase (Sigma) and plated onto laminin-coated wells at a ratio of 1:2 in the following manner: cells were washed with PBS and subsequently incubated with 500 µL Accutase solution at 37°C for 5-10 min. To loosen all cells and split up clumps, gentle trituration and collection of cells into 9 mL NMM was performed using a P1000. This cell-suspension was centrifuged at 300 g for 5 min (Heraeus Megafuge 8 centrifuge (Thermo Scientific)), the supernatant removed and the pellet re-suspended in NMM with 10 µM Y-27632 for plating. The next day, cells were given fresh NMM. The resulting neural progenitor cells (NPCs) were generally expanded a second time using Accutase, before being frozen down between days 28-32 post initiation of neural induction. Freezing involved lifting with Accutase, and re-suspending the centrifuged cell-pellet in ice-cold freezing media consisting of: 90% vol/vol NMM, 10% vol/vol dimethylsulfoxide (DMSO) (Sigma) and 20 ng/mL FGF2 (Peprotech), quickly aliquoting in cryovials at 1.5×10^6 cells/vial and storing them overnight in a CoolCell freezing container (Corning) at -80°C to facilitate slow freezing, and moving them to liquid nitrogen (LiN_2) storage the following day.

Differentiation of iPSCs to cortical astrocytes

The protocol accompanying the astrocyte differentiation and maturation kits from STEMCELL technologies was adapted to start from a neural rosette stage. Neural rosettes were generated as described above and grown on laminin and the AstroDiff media was applied around day 20-25 of neural induction. Astrocyte progenitors were kept in AstroDiff for 20 days (until day 40-45) and underwent 3 expansions, every time when cells reached ~90% confluency. Cells were lifted with Accutase (Sigma), washed in AMM, centrifuged at 300 g for 5 min and re-suspended in AstroDiff for re-plating at $1x10^5$ cells/cm^2. At the 3rd passage, the majority of cells were frozen down as progenitor stock in 90% vol/vol AstroDiff media with 10% vol/vol DMSO (Sigma). Media was then changed to AstroMature for a further 15 days (until day 55-60), during which astrocyte precursors also underwent 3 passages with Accutase.

Similarly, at the 6th passage, cells could be frozen down as astrocyte precursor stock in 90% vol/vol AstroMature media with 10% vol/vol DMSO. Maintaining the astrocytes beyond the manufacturer's protocol was done in astrocyte maintenance (AMM: 45% vol/vol Neurobasal, 45% vol/vol DMEM/F12 Glutamax containing 1 mM L-glutamine, 50 U/mL penicillin and 50 mg/mL streptomycin and 10% filtered FBS***)***. Final maturation was thus conducted in AMM media for 20-30 days (until day 75-90) and if cells were still proliferating, further passages were conducted, but using Trypsin/EDTA (Gibco) and washing in serum-containing AMM. Media was changed every 2-3 days throughout the differentiation protocol. Cryo-stored stocks of astrocyte progenitors and precursors could be thawed and the protocol resumed from the point they were frozen down.

Generation and maintenance of cortical rat astrocytes

Cortical rat astrocytes were either obtained from Gibco and grown in glial medium (DMEM with 15% vol/vol filtered FBS), or generated from P1 rat cortices (Wistar rats; Charles River Laboratories) according to (Kaech and Banker, 2006), and in accordance with the Animals (Scientific Procedures) Act, 1986 (United Kingdom). For experimentation, rat astrocytes were seeded onto 13 mm acid-washed and sterilized glass coverslips, coated with 0.1 mg/mL poly-D-lysine (Sigma), and grown until confluent.

Lentiviral vectors

Vector	Virus	Source	Identifier	Reference
**pLenti-CAG-hChR2(H134R)-EYFP-WPRE**	LV-CAG-ChR2-YFP	A gift from Karl Deisseroth	Addgene plasmid # 20945<br><br>RRID: Addgene_20945	(Zhang et al., 2007)
**pLenti-CaMKIIa-mKate2-WPRE**	LV-CamKIIa-mKate2	A gift from Ricardo Dolmetsch and Oleksandr Shcheglovitov	Addgene plasmid # 96942<br><br>RRID: Addgene_96942	(Shcheglovitov et al., 2013)

HEK293T cells for transfection were plated in a T75 flask (Corning) at a density of $6.5x10^6$ cells/flask in growth medium consisting of: 90% vol/vol DMEM (Gibco) and 10% vol/vol FBS (Gibco). Cells were grown to 60% confluency and media supplemented with 1x NEAAs (Gibco) prior to transfection. Cells were transfected according to the protocol by (Tashiro et al., 2015) with 100 µL Lipofectamine 2000 (Invitrogen), 9.375 µg vector DNA, 3.125 µg VSVg envelope DNA and 8.75 µg Δ8.91 packaging DNA made up in Opti-MEM (Gibco) in the following way: Lipofectamine was added to 1 mL Opti-MEM and incubated at room temperature for 5 min. Separately, DNA was added to 1 mL Opti-MEM and then transferred to the Lipofectamine solution, inverting to mix solutions. The mixture was incubated for a further 25-30 minutes before addition to HEK cells. Transfection medium was replaced with fresh growth medium 5 hours later.

Media containing the viral particles was harvested after 48 hours, centrifuged at 1000 g for 3 minutes to remove cell debris, and then passed through a 0.45 µm filter before aliquoting. If cells had not detached significantly, a second harvest could be obtained by adding media and collecting it 48 hours later. Supernatant was either stored at -80 ^{o}C or further processed by ultracentrifugation to concentrate the viral particles. For ultracentrifugation, 8 mL supernatant was transferred into conical bottomed, heat sealed tubes (Beckman Coulter) and ultracentrifuged at 30,000 rpm in a SW41TI swinging bucket rotor at 4°C for 3 hours (Beckman L8-70M Ultracentrifuge). The supernatant was subsequently extracted and each viral pellet was re-suspended in

20 µL Opti-MEM with 1:100 DNase I (1 U/µL stock concentration, Sigma), left overnight at 4 ^{o}C, and lastly aliquoted and stored at -80 ^{o}C.

Immunocytochemistry

Cells were pre-fixed in 2% PFA for 10 min, followed by fixation in 4% PFA for 10 min, washed three times in PBS, then permeabilized with 0.3% vol/vol Triton-X (TX) for 15 min, and blocked in PBS with 1.5% vol/vol Triton X-100 and 10% normal goat serum (NGS, Sigma) for 1h. Primary antibodies (see table below) were diluted in PBS with 0.15% Triton X-100 and 2% NGS and incubated with cells overnight at 4°C (long incubation of 48h used for synaptic antibodies). The next day, cells were washed three times in PBS and incubated with the relevant Alexa Fluor conjugated secondary antibodies (see table below) for 1h at room temperature (or overnight at 4°C for synaptic antibodies). Cells were washed three times in PBS, including one wash containing 1:5000 DAPI (D1306 ThermoFisher Scientific; RRID:AB_2629482), and finally mounted with ProLong Gold (ThermoFisher Scientific). Images were collected on an epifluorescence microscope (BX40, Olympus) and quantification of GFAP, S100β and Tuj1 positive cells was performed using the Cell-counter plug-in for Image J (Fiji).

Primary antibodies

Antibody	Raised in	Concen-tration	Supplier	Catalogue #	RRID
**GFAP**	Mouse IgG1	1:1000	Millipore	MAB360	AB_11212597
**Ki67**	Mouse IgG1	1:600	BD bioscience	550609	AB_393778
**MAP2A**	Mouse IgG1	1:1000	Millipore	MAB378	AB_11214935
**Otx2**	Rabbit	1:500	Millipore	ab9566	AB_2157186
**Pax6**	Rabbit	1:500	Covance	PRB-278P	AB_291612
**Synapto-physin**	Guinea Pig	1:1000	Synaptic Systems	101004	AB_1210382
**S100β**	Rabbit	1:800	Abcam	ab868	AB_306716
**Tuj1**	Mouse IgG2a	1:2000	Biolegend	B199846	AB_2313773

Secondary antibodies

Antibody	Conjugate	Raised in	Concen-tration	Supplier	Catalogue #	RRID
**Anti-mouse**	Alexa Fluor 488	Goat	1:1000	Life Tech.	A11029	AB_2534088
**Anti-mouse IgG1**	Alexa Fluor 488	Goat	1:500	Life Tech.	A21121	AB_2535764
**Anti-mouse**	Alexa Fluor 568	Goat	1:1000	Life Tech.	A11031	AB_144696
**Anti-mouse IgG2a**	Alexa Fluor 568	Goat	1:500	Life Tech.	A21134	AB_2535773
**Anti-mouse**	Alexa Fluor 633	Goat	1:500	Life Tech.	A21052	AB_2535719
**Anti-mouse IgG1**	Alexa Fluor 680	Goat	1:500	Life Tech.	A31562	AB_2536176
**Anti-rabbit**	Alexa Fluor 488	Goat	1:1000	Life Tech.	A11034	AB_2576217
**Anti-rabbit**	Alexa Fluor 568	Goat	1:1000	Life Tech.	A11036	AB_10563566
**Anti-guinea pig**	Alexa Fluor 488	Goat	1:500	Life Tech.	A11073	AB_2534117
**Anti-rat**	Alexa Fluor 488	Goat	1:1000	Life Tech.	A11006	AB_2534074

Measuring glutamate uptake currents

Experiments for detecting glutamate uptake with whole-cell patch clamp recordings followed previous work, and used the fact that the net ionic flux of astrocytic glutamate transporters is positive, and so can be detected as an inward current (Dallas et al., 2007). L-glutamate was first diluted in NaOH to 100 mM, then further diluted to a stock concentration of 10 mM in dH_2O, and used at a final concentration of 100 µM by dilution in ACSF. The biophysical properties of cells were first confirmed to correspond to putative astrocytes in terms of low $VGNa^+$ currents and lack of action potential initiation. Astrocytes were held in voltage clamp mode at -70 mV throughout a baseline period in normal ACSF, and then during wash-in of glutamate-containing ACSF for 1-

1.5 min. To determine if an astrocyte exhibited an inward current associated with glutamate uptake, 10 equally-spaced current measurements during the baseline period were compared to 10 equally-spaced current measurements in the glutamate-containing ACSF (Wilcoxon signed-rank test, $p<0.05$). The amplitude of the glutamate-associated uptake current was defined as the peak current shift in glutamate-ACSF, compared to the mean current during a 1 min baseline period in normal ACSF.

Glutamate uptake assay

An enzymatic glutamate detection kit (Sigma, MAK004) was used to assess glutamate uptake by iPSC-astrocytes. As astrocytic glutamate uptake is a Na^+-dependent process, the degree of glutamate uptake was determined by comparing cultures in Na^+-containing media, to those in Na^+-free media. Cells were first grown to confluency in 6-well plates and equilibrated with HBSS for 10 min, before the addition of 50 µM L-glutamate diluted in either normal HBSS or Na^+-free HBSS. Media samples were collected after 60 minutes and kept on ice. Cells were lifted with trypsin/EDTA, washed, pelleted and subsequently lysed in 500 µL ice-cold RIPA buffer (Thermo Fisher Scientific) containing phosphatase and proteinase inhibitors (Sigma). Cell debris was removed by centrifuging the lysate at 13,500 RPM for 5 min at 4°C. The enzymatic assay was conducted in 96-well plate format, by adding 100 µL reaction mix to each 50 µL media sample or diluted glutamate standard. The plate was incubated for 30 min at 37°C, protected from light and then the absorbance was measured at 450 nm on a Sunrise F039300 plate-reader (Tecan). All values were normalized to protein content, determined by running an accompanying BCA protein detection assay on the cell lysates (Thermo Scientific, 23227). The amount of glutamate uptake by the iPSC-astrocytes was calculated by subtracting the glutamate concentration remaining in the Na^+-containing media, from the glutamate concentration remaining in the Na^+-free media. Results are expressed as glutamate uptake by astrocytes above the Na^+-free condition, per amount of protein, per minute.

Calcium imaging

Cells were viewed using a BX51WI microscope equipped with a 20x/1.00NA XLUMPlanFL-N objective (Olympus) and iXon camera (Andor Technologies), using a 480/40 nm excitation filter, 505 nm dichroic and 520 nm emission filter. Astrocytes were loaded with 10 µM OGB-1 AM ester (Life Technologies, 06807), mixed from equal amounts of dye and Pluronic F-127 (20% solution in DMSO, Invitrogen), before being diluted and vortexed in AMM. The final mixture of dye, pluronic and media was added to the astrocytes and incubated for 60 min at 37°C. Following loading, dye-containing media was removed and cells incubated in normal media for 30 min and subsequently transferred to the recording chamber and continuously perfused with external solution (in mM: 140 NaCl, 5 KCl, 2 CaCl2, 10 HEPES and 10 glucose). The size of Ca^{2+} events was calculated as ΔF/F according to: $\frac{\Delta F}{F} = \frac{F_t - F_{t=0}}{F_{t=0}}$, where $F_{t=0}$ was the mean of at least the first 5 frames of a movie. Events were detected with a ΔF/F threshold of 1.05. Spontaneous events were detected from 1-2 min movies (2-4 Hz frame rate) and classified as synchronous if they initiated within 1 s of one another. Neurotransmitter-evoked events were studied by delivering focal puffs of agonist via a patch-pipette (containing 100 µM glutamate or 1 mM ATP in external solution), controlled by a micro-manipulator (Sutter Instrument Company) and attached to Picospritzer II (General Valve Corporation; 1-2 psi). Brightness and contrast was adjusted on all imaging files in Image J, along with histogram correction via the plugin from EMBL tools, Bleach correction. ROIs were placed over cell bodies or cell-free background areas. Data from all ROIs was extracted as mean grey values from every image via the Multi-measure function.

Data analysis of electrophysiological recordings

Electrophysiological data was acquired with a Multiclamp 700B amplifier and the accompanying software Clampex (version 10.5, Molecular Devices). Signals were digitized with an Axon Digidata 1550 (Molecular devices), filtered at 2 kHz (Bessel filter) and sampled at 20 kHz. Data analysis was conducted with Clampfit software (version 10.5, Molecular Devices). Automated measurements of access resistance (Ra), membrane capacitance (Cm) and membrane resistance (Rm) were logged through the Clampex membrane test function. To be included for analysis, cells required Ra values below 50 MΩ, and the Ra, Cm and Rm values before and after each recording could not deviate by more than +/- 20% from the mean. Voltage gated currents: The leak subtracted trace was used for analysis of sodium (Na^+) currents, for which the largest negative VG current was reported. Potassium (K^+) current behaviours were assessed from the non-leak subtracted traces and were classified based on their current-voltage (I-V) profiles. Automated sEPSC event detection was performed with the template fitting function in Clampfit. Subsequent post-hoc filtering of putative sEPSC events used criteria for amplitude, half width and rise time (>7 pA, <4 ms and <1.2 ms, respectively), designed to exclude false-positive events and validated through comparison against manual detection. Cells with a sEPSC frequency of <0.017 Hz (i.e. less than 1 event per min) were regarded as having a sEPSC frequency of 0.

RNA sequencing and quantification

Briefly, a custom transcriptome index was created based on cDNA and ncRNA fasta sequences downloaded from Ensembl release 91 (Aken et al., 2016), keeping sequences only from chromosomes 1, 2, …, 22, X and Y. Estimated counts at the transcript level could be obtained using kallisto and summarized to gene level using the tximport R package (Soneson et al., 2015). High mapping rates (~92%) across all iPSC-astrocyte samples were observed and, on average, 13,057 protein coding genes were detected per sample (with at least one transcript per million (TPM)). For accurate comparison, transcript abundances were quantified in the same way as described above for GSE73721 (Zhang et al., 2016), GSE102956 (Lin et al., 2018), GSE97619 (Santos et al., 2017), GSE97904 (Tcw et al., 2017), and fastq files kindly provided by Lischka (Lischka et al., 2018). For comparison across datasets, counts for protein coding genes were summarized and genes with zero counts and genes with zero variance across all samples were removed. Next, counts were transformed using the logarithm Log2 (counts + 1) before applying a quantile normalization using the preprocessCore R library (Bolstad, 2018). Primary fetal and adult astrocyte samples from human tissue (Zhang et al., 2016) were selected to perform gene expression principal component analysis (R prcomp). Then, the gene expression profiles of iPSC-astrocytes were projected onto the principle component space by multiplying the centered data to the principal component loadings.

Differential expression analysis

Comparison of the expression profiles of our iPSC-astrocytes and those published by Lischka (Lischka et al., 2018) was performed by differential expression analysis using DESeq2 (Love et al., 2014). We acknowledge that possible technical confounds could contribute to the gene expression differences observed between datasets of iPSC-astrocytes (library preparation, number of reads detected, mapping rate, etc.). Yet, we considered only those expression differences that were also different when comparing fetal and mature astrocytes from other data (see **Results**) and believe these confounds are unlikely to contribute to both sets of differences. Lowly expressed genes (with less than one TPM across the samples of both datasets) were removed through pre-filtering. DESeq2 default filtering removed gene outliers, after which 14,629 genes remained; this set was considered as the gene background population for further gene functional analyses. A list of genes that were up and down regulated in primary fetal versus primary adult cortical astrocytes (reported in Table S6 by Zhang et al. (2016)) were used for comparison. HGNC symbols were converted into Ensemble gene IDs through biomaRt (Durinck et al., 2009). Genes were considered differentially expressed when the false detection rate (FDR) was < 0.05. Hypergeometric testing was employed to evaluate the significance of the overlap between two lists of genes.

To examine astrocyte reactive genes, we made use of two overlapping signatures of reactive astrocytes, which have been reported in mice (Zamanian et al., 2012), one in response to ischemic insult by occlusion of the middle cerebral artery (MCAO) and the other in response to inflammation upon administration of bacterial lipopolysaccharide (LPS). The top 50 differentially expressed genes found by Zamanian et al. (2012) in each condition and their corresponding controls reported in Table 1 and Table 2 (Zamanian et al., 2012) were included in the comparative analysis.

Gene Ontology analysis

Gene Ontology (GO) annotations were obtained from Ensembl through biomaRt (Durinck et al., 2009). A subset of GO annotations that contained at least 50 genes in our background gene population (large enough to be informative and powerful) was selected and tested for enrichment using a hypergeometric test. The corresponding p-values were adjusted to account for multiple testing (Benjamini-Hotchberg FDR), and a GO term was considered as overrepresented when the FDR < 0.05. For visualization purposes, an intelligible list of overrepresented GO terms was sought and thus REVIGO was used to identify redundant terms (Supek et al., 2011).

Protein-protein interaction analysis

To identify protein-protein interactions between extracellular astrocytic and synaptic genes, a range of sources were integrated: BioGRID (Stark et al., 2006) (retrieved on 09/04/2019), HitPredict (Lopez et al., 2015) (09/04/2019), IntAct (Orchard et al., 2014) (09/04/2019), STRING (Szklarczyk et al., 2019) (09/04/2019), CORUM (Giurgiu et al., 2019) (09/04/2019), Reactome (Fabregat et al., 2018) (09/04/2019), BioPlex2.3 (Huttlin et al., 2017) (09/04/2019), MINT (Licata et al., 2012) (15/09/2017), InBioMap (Li et al., 2017) (15/09/2017). Interactions at the gene level were summarized (Ensembl gene IDs), while removing duplicated interactions

across datasets and self-interacting pairs. Note that only protein links with an experimental score > 0 from the STRING database were considered. Among the set of genes with both higher expression in our iPSC-astrocytes compared to those from Lischka, and higher expression in adult compared to fetal astrocytes from Zhang, the subset of extracellular genes were identified as those annotated to extracellular Gene Ontology terms (GO:0070062, GO:0005576 or GO:0005615). Synaptic genes were identified by using the Obo-Edit software to retrieve all children terms of synapse parts (GO:0044456). Terms were grouped into "pre-synaptic" or "post-synaptic" if these words were found in the term description. Finally, all genes were grouped separately according to their annotation to either the 33 "pre-synaptic" or 47 "post-synaptic" terms.

We tested whether more protein-protein interactions were observed than expected by chance between two sets of genes (i.e. extracellular genes identified in iPSC-astrocytes and genes annotated as either pre- or post-synaptic). First, the number of protein-protein interactions between the two gene sets was counted, then compared to the number of interactions found in each of 10,000 random samples of genes of equal size as the first list (matched for both network node degree and coding sequence length) (Honti et al., 2014). A p-value could then be estimated by counting the number of random samples with an equal or higher number of protein-protein interactions in the real data. Finally, Cytoscape was used for visualization of the protein-protein interaction network (Shannon et al., 2003). To relate these data to different brain cell types, single cell RNA seq expression from diverse brain cell types (Darmanis et al., 2015) were normalized before averaging by cell type (62 astrocytes, 20 endothelial cells, 16 microglia, 131 neurons, 38 oligodendrocytes, and 18 oligodendrocyte precursor cells), and finally the expression was scaled across cell types to create a heatmap.

Supplemental references:

Dallas, M., Boycott, H.E., Atkinson, L., Miller, A., Boyle, J.P., Pearson, H.A., and Peers, C. (2007). Hypoxia suppresses glutamate transport in astrocytes. J. Neurosci. *27*, 3946–3955.

Durinck, S., Spellman, P.T., Birney, E., and Huber, W. (2009). Mapping identifiers for the integration of genomic datasets with the R/ Bioconductor package biomaRt. Nat. Protoc. *4*, 1184–1191.

Fabregat, A., Jupe, S., Matthews, L., Sidiropoulos, K., Gillespie, M., Garapati, P., Haw, R., Jassal, B., Korninger, F., May, B., et al. (2018). The Reactome Pathway Knowledgebase. Nucleic Acids Res. *46*, D649–D655.

Giurgiu, M., Reinhard, J., Brauner, B., Dunger-Kaltenbach, I., Fobo, G., Frishman, G., Montrone, C., and Ruepp, A. (2019). CORUM: the comprehensive resource of mammalian protein complexes-2019. Nucleic Acids Res *47*, D559–D563.

Honti, F., Meader, S., and Webber, C. (2014). Unbiased functional clustering of gene variants with a phenotypic-linkage network. PLoS Comput Biol *10*, e1003815.

Huttlin, E.L., Bruckner, R.J., Paulo, J.A., Cannon, J.R., Ting, L., Baltier, K., Colby, G., Gebreab, F., Gygi, M.P., Parzen, H., et al. (2017). Architecture of the human interactome defines protein communities and disease networks. Nature *545*, 505–509.

Kaech, S., and Banker, G. (2006). Culturing hippocampal neurons. Nat. Protoc. *1*, 2406–2415.

Li, T., Wernersson, R., Hansen, R.B., Horn, H., Mercer, J., Slodkowicz, G., Workman, C.T., Rigina, O., Rapacki, K., Staerfeldt, H.H., et al. (2017). A scored human protein-protein interaction network to catalyze genomic interpretation. Nat Methods *14*, 61–64.

Licata, L., Briganti, L., Peluso, D., Perfetto, L., Iannuccelli, M., Galeota, E., Sacco, F., Palma, A., Nardozza, A.P., Santonico, E., et al. (2012). MINT, the molecular interaction database: 2012 update. Nucleic Acids Res *40*, D857-61.

Lopez, Y., Nakai, K., and Patil, A. (2015). HitPredict version 4: comprehensive reliability scoring of physical protein-protein interactions from more than 100 species. Database (Oxford) *2015*.

Love, M.I., Huber, W., and Anders, S. (2014). Moderated estimation of fold change and dispersion for RNA-seq data with DESeq2. Genome Biol *15*, 550.

Orchard, S., Ammari, M., Aranda, B., Breuza, L., Briganti, L., Broackes-Carter, F., Campbell, N.H., Chavali, G., Chen, C., del-Toro, N., et al. (2014). The MIntAct project--IntAct as a common curation platform for 11

molecular interaction databases. Nucleic Acids Res *42*, D358-63.

Shannon, P., Markiel, A., Ozier, O., Baliga, N.S., Wang, J.T., Ramage, D., Amin, N., Schwikowski, B., and Ideker, T. (2003). Cytoscape: a software environment for integrated models of biomolecular interaction networks. Genome Res *13*, 2498–2504.

Shcheglovitov, A., Shcheglovitova, O., Yazawa, M., Portmann, T., Shu, R., Sebastiano, V., Krawisz, A., Froehlich, W., Bernstein, J.A., Hallmayer, J.F., et al. (2013). SHANK3 and IGF1 restore synaptic deficits in neurons from 22q13 deletion syndrome patients. Nature *503*, 267–271.

Stark, C., Breitkreutz, B.J., Reguly, T., Boucher, L., Breitkreutz, A., and Tyers, M. (2006). BioGRID: a general repository for interaction datasets. Nucleic Acids Res *34*, D535-9.

Supek, F., Bosnjak, M., Skunca, N., and Smuc, T. (2011). REVIGO summarizes and visualizes long lists of gene ontology terms. PLoS One *6*, e21800.

Szklarczyk, D., Gable, A.L., Lyon, D., Junge, A., Wyder, S., Huerta-Cepas, J., Simonovic, M., Doncheva, N.T., Morris, J.H., Bork, P., et al. (2019). STRING v11: protein-protein association networks with increased coverage, supporting functional discovery in genome-wide experimental datasets. Nucleic Acids Res *47*, D607–D613.

Tashiro, A., Zhao, C., Suh, H., and Gage, F.H. (2015). Preparation and Use of Retroviral Vectors for Labeling, Imaging, and Genetically Manipulating Cells. Cold Spring Harb. Protoc. *2015*, 883–888.

Zamanian, J.L., Xu, L., Foo, L.C., Nouri, N., Zhou, L., Giffard, R.G., and Barres, B.A. (2012). Genomic Analysis of Reactive Astrogliosis. J. Neurosci. *32*, 6391–6410.

Zhang, F., Wang, L.P., Brauner, M., Liewald, J.F., Kay, K., Watzke, N., Wood, P.G., Bamberg, E., Nagel, G., Gottschalk, A., et al. (2007). Multimodal fast optical interrogation of neural circuitry. Nature *446*, 633–639.

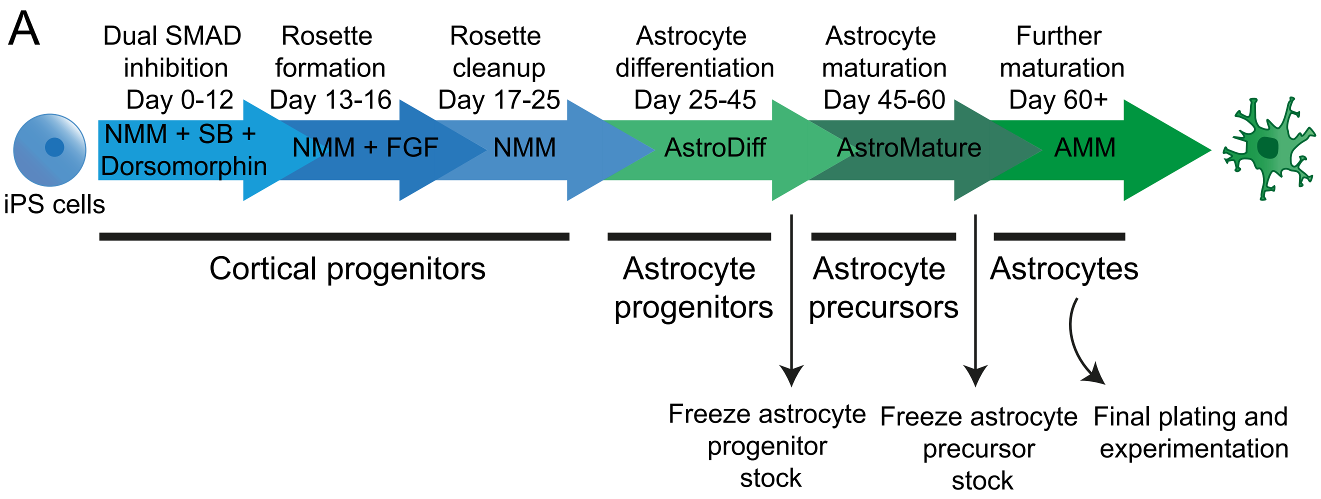

Figure S1. Outline of the astrocyte differentiation protocol. Related to Figure 1. **(A)** Up to day 17-25, the iPSCs follow the protocol for cortical neural induction via dual SMAD inhibition, specified by the (Shi et al., 2012). Around day 25, clean rosettes are switched from a neural maintenance media (NMM) to astrocyte differentiation media (AstroDiff), and subsequently dissociated to single cells once per week. Stocks of proliferative astrocyte progenitors can be frozen and cryostored in liquid nitrogen before switching to astrocyte maturation media around day 45. Weekly dissociations and seeding at lower density yield a population of less proliferative astrocyte precursors, which can also be frozen and cryostored. Further maturation is achieved by switching the media to astrocyte maintenance media (AMM) around day 60, in which astrocytes can be finally plated and maintained for experimentation.

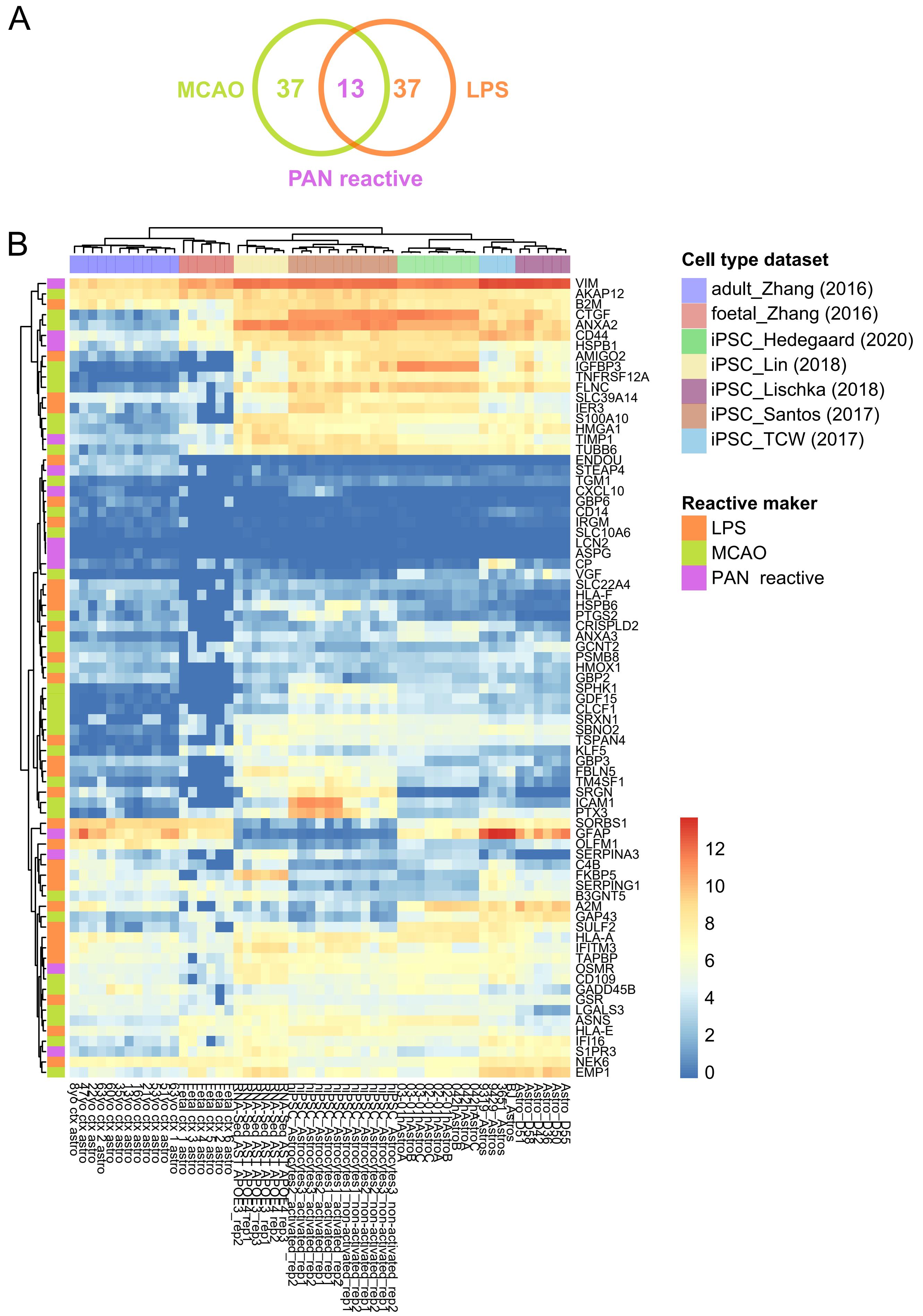

Figure S2. iPSC-astrocytes exhibit similar reactive states across datasets. Related to Figure 1. **(A)** Venn-diagram shows the number of genes associated with an ischemic state after middle cerebral artery occlusion (MCAO), an inflammatory state following stimulation with lipopolysaccharide (LPS), or both (PAN reactive), identified in (Zamanian et al., 2012). **(B)** Comparison of transcriptomic datasets from primary human astrocytes and multiple iPSC-astrocytes (color-coded by cell type and publication). Gene expression is given in log transformed counts per million (log2 (CPM + 1)), scaled by gene.

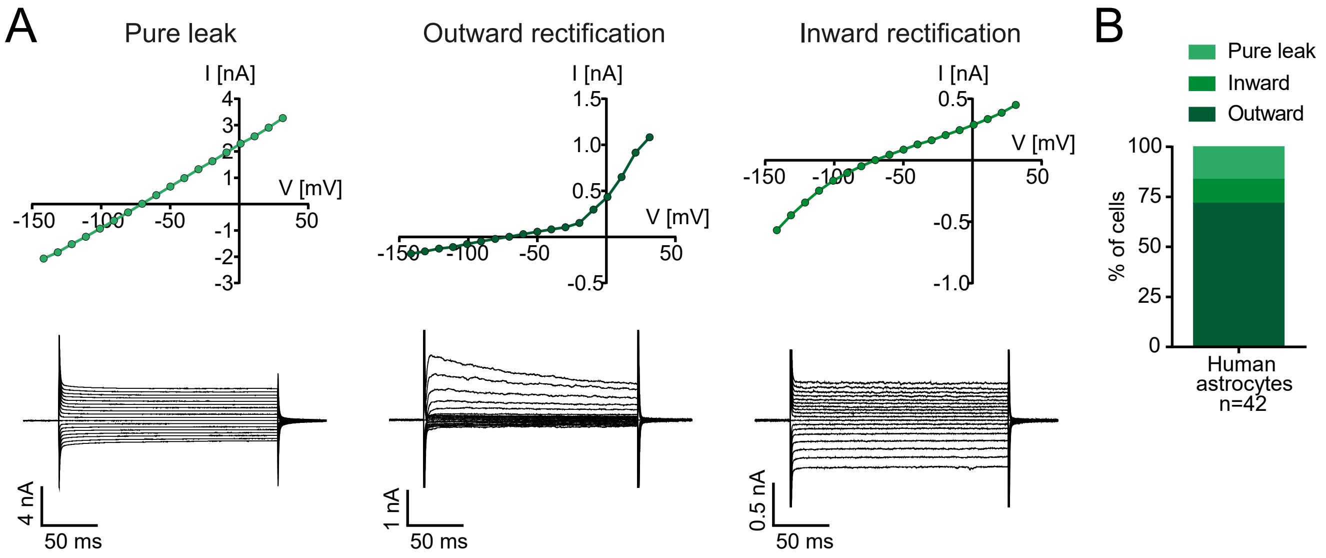

Figure S3. K⁺-current profiles of cortical iPSC-astrocytes. Related to Figure 2. (A) Examples of different K^+ currents exhibited by iPSC-astrocytes. I-V plots (top) and accompanying non-leak subtracted family of current traces elicited by subjecting an astrocyte to a series of voltage steps (bottom). Astrocytes, aged 97 ± 3.5 days showed either linear 'pure leak' (left), outward rectifying (middle), or inward rectifying (right) K^+ currents. **(B)** The majority of astrocytes showed outward rectifying K^+ currents (71%), but a subset displayed purely leak K^+ currents (17%) or inward rectifying K^+ currents (12%). Of the five astrocytes displaying inward rectification, two were also tested for glutamate uptake currents and both exhibited this property.

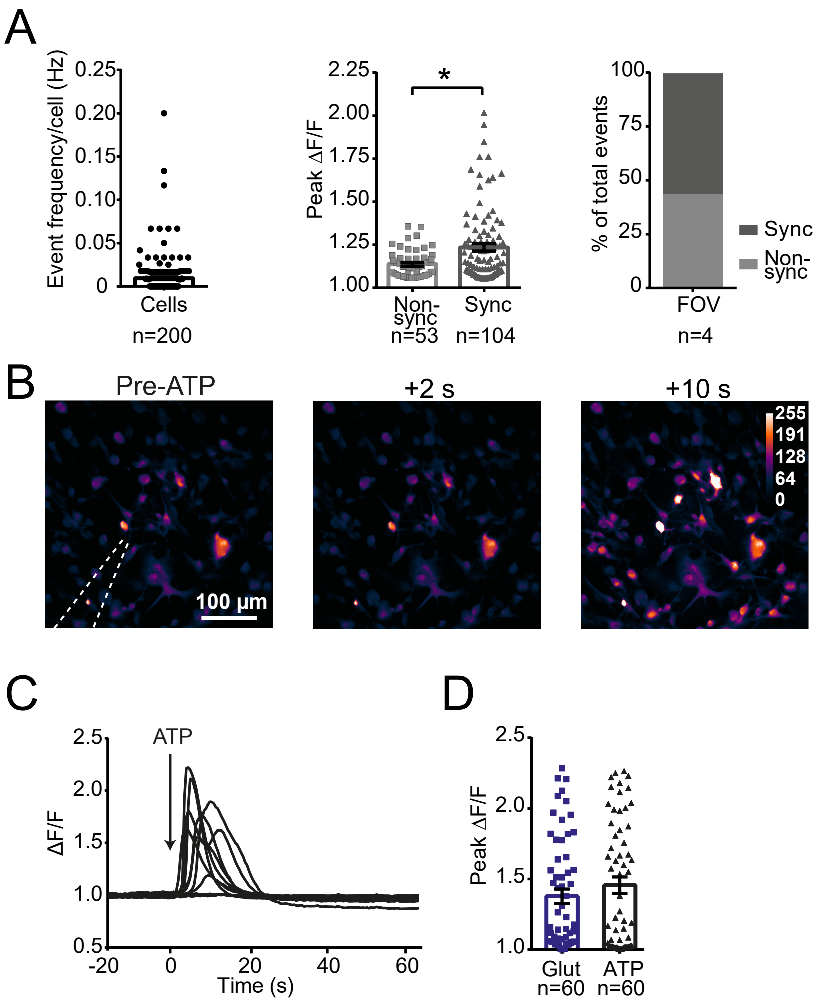

Figure S4. Spontaneous and evoked Ca^{2+} responses in iPSC-astrocytes. Related to Figure 3. **(A)** Quantifications of spontaneous Ca^{2+} events from the same 4 FOV's as shown in Figure 3, representing 4 cultures across 3 cell lines aged 101 ± 3.9 days. 50 random ROIs were drawn on each FOV, so n=200 cells. The frequency of Ca^{2+} events per cell (left), the peak ΔF/F amplitude of non-synchronous versus synchronous events (middle) and the proportion of event types (43% non-synchronous, 57% synchronous, right). **(B)** Ca^{2+} events elicited by a 500 ms puff of 1 mM ATP, delivered via a patch-pipette. **(C)** ΔF/F traces from 10 representative ROIs from the FOV shown in 'B'. **(D)** Comparison of Ca^{2+} event amplitudes between events evoked by glutamate and ATP in a 96 days old culture (2 FOVs per transmitter, each with 30 ROIs, so n=60). FOV: fields of view. * $p < 0.05$

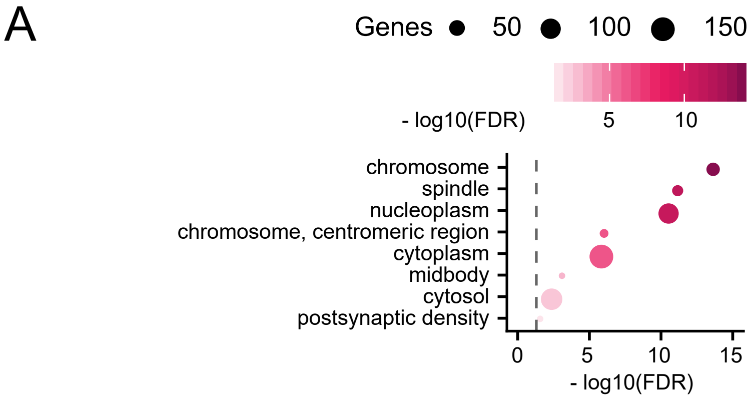

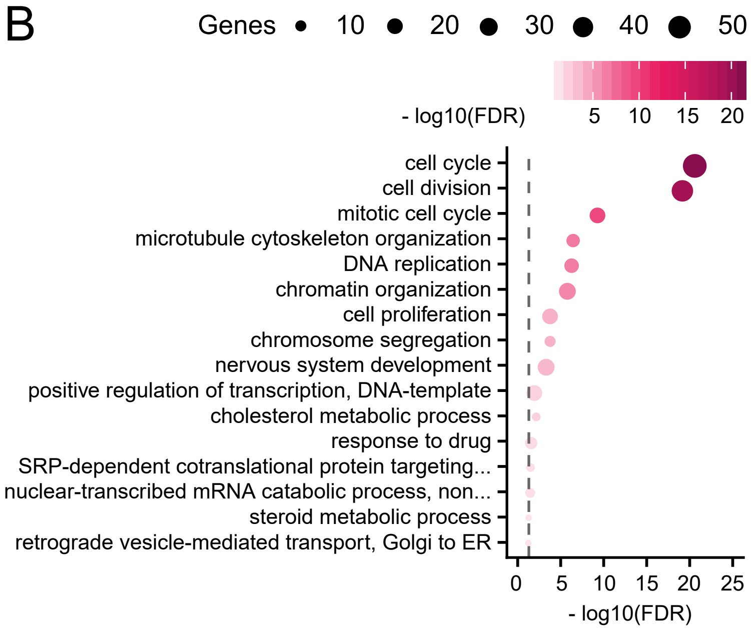

Figure S5. Relative astrocyte immaturity is associated with nuclear components and proliferative processes in iPSC-astrocytes. Related to Figure 6. **(A)** Overrepresented cellular components and **(B)** biological processes among the 279 genes with higher expression in both primary fetal astrocytes (compared to adult) and the ‘iPSC Lischka’ astrocytes, compared to ‘iPSC Hedegaard’. Size of the circles indicate the number of genes annotated to each GO terms while colours reflect the log10 transformed FDR. GO: gene ontology, FDR: false discovery rate.

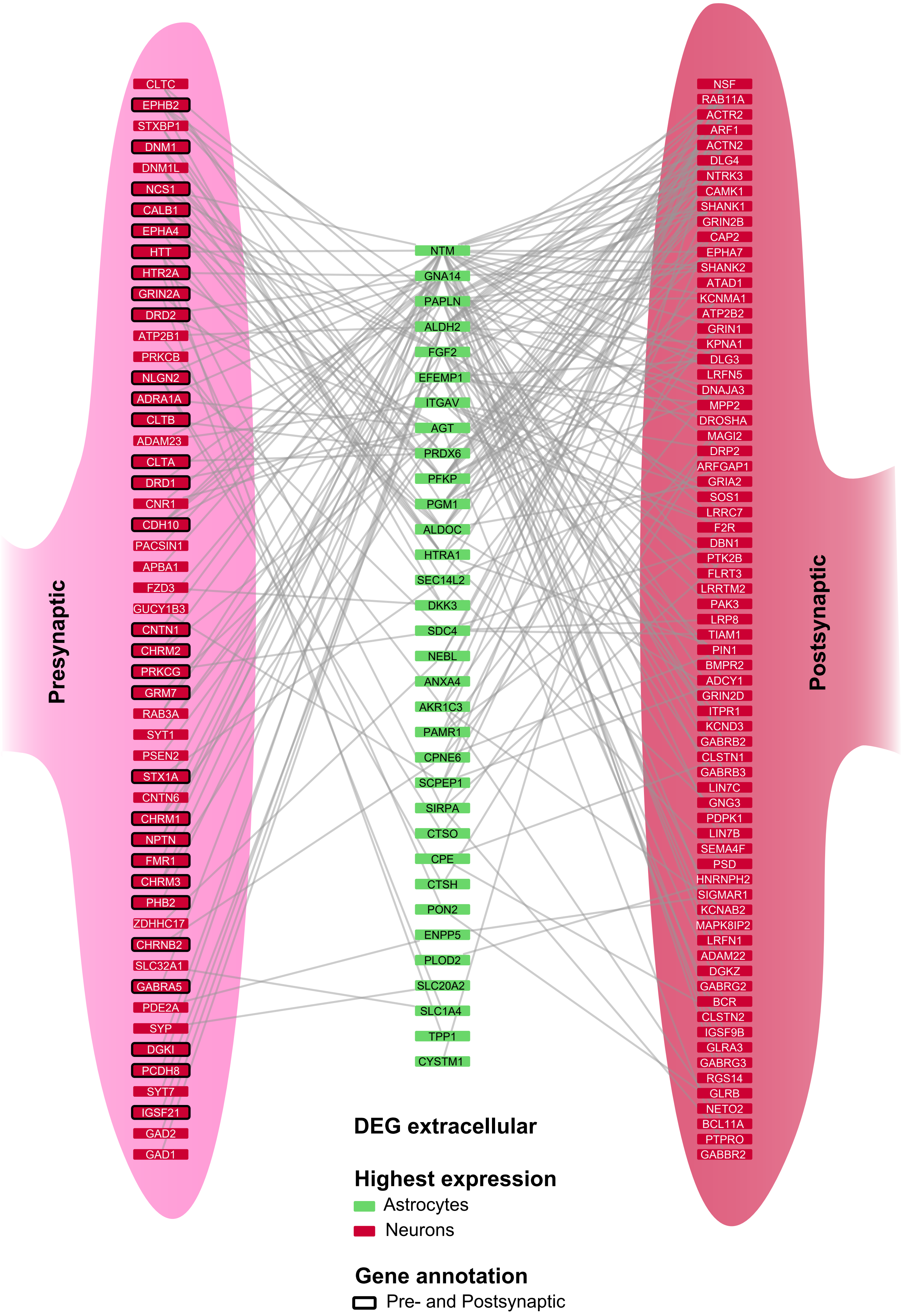

Figure S6. Expanded protein-protein interaction network. Related to Figure 7. Enlarged view of a subset of the protein-protein interaction network shown in Figure 7B. Protein interactions are highlighted between extracellular genes that are most highly expressed in astrocytes (green), and synaptic genes that are most highly expressed in neurons (red). The astrocyte extracellular genes are ranked from top to bottom based on the number of interactions that their proteins show with pre-synaptic (left) and post-synaptic (right) proteins. A black border highlights genes annotated to both pre-and post-synaptic sets.
